# Evaluating and comparing biomarkers with respect to the area under the receiver operating characteristics curve in two-phase case–control studies

**DOI:** 10.1093/biostatistics/kxw003

**Published:** 2016-02-16

**Authors:** Ying Huang

**Affiliations:** Fred Hutchinson Cancer Research Center, Seattle, WA 98109, USA and Department of Biostatistics, University of Washington, Seattle, WA 98109, USA

**Keywords:** AUC, Biased sampling, Frequency match, Inverse probability weighting, ROC curve, Two-phase studies

## Abstract

Two-phase sampling design, where biomarkers are subsampled from a phase-one cohort sample representative of the target population, has become the gold standard in biomarker evaluation. Many two-phase case–control studies involve biased sampling of cases and/or controls in the second phase. For example, controls are often frequency-matched to cases with respect to other covariates. Ignoring biased sampling of cases and/or controls can lead to biased inference regarding biomarkers' classification accuracy. Considering the problems of estimating and comparing the area under the receiver operating characteristics curve (AUC) for a binary disease outcome, the impact of biased sampling of cases and/or controls on inference and the strategy to efficiently account for the sampling scheme have not been well studied. In this project, we investigate the inverse-probability-weighted method to adjust for biased sampling in estimating and comparing AUC. Asymptotic properties of the estimator and its inference procedure are developed for both Bernoulli sampling and finite-population stratified sampling. In simulation studies, the weighted estimators provide valid inference for estimation and hypothesis testing, while the standard empirical estimators can generate invalid inference. We demonstrate the use of the analytical variance formula for optimizing sampling schemes in biomarker study design and the application of the proposed AUC estimators to examples in HIV vaccine research and prostate cancer research.

## 1. Introduction

Recent advances in lab techniques have provided researchers with a rich resource of biomarkers potentially useful for disease diagnosis and risk prediction. It is essential to use proper statistical methods to rigorously evaluate these biomarkers. The receiver operating characteristics curve (ROC) is a standard graphic tool to characterize a biomarker's classification accuracy. The area under the ROC curve (AUC) has been commonly used to gauge and compare biomarker's performance. In this paper, we consider the evaluation and comparison of biomarkers with respect to AUC for a binary disease outcome using data from two-phase sampling designs. In the first phase, a cohort sample representative of the target population relevant to clinical application is drawn, from which participants' disease status and easy to measure covariates are obtained; in the second phase, a subsample is drawn randomly, without replacement from the phase-one cohort sample for biomarker measurement, where sampling probability of each individual can depend on other covariates available. In particular, we consider studies where cases and controls in the second phase are separately sampled from the phase-one cohort. This is different from, for example, a case–cohort design for failure time, where cases and a random cohort are separately sampled. These types of designs prospectively collect bio-specimens before outcome ascertainment to minimize systematic difference in specimen collection, and retrospectively sample cases and controls for measuring biomarkers from the stored specimens to save costs. They have been proposed as gold standards for biomarker evaluation (Pepe *and others*, 2008).

In the second phase of a two-phase study, oftentimes cases and/or controls are not simple random samples from their respective distributions. For example, controls are often frequency-matched to cases with respect to other covariates, such as an individual's demographic characteristics (i.e., gender, age group, etc.); or cases and controls can be randomly sampled within some covariate strata. The effect of biased sampling on biomarker evaluation varies with the parameter of interest. Janes and Pepe (2009) showed that frequency matching does not invalidate inference on a biomarker's classification accuracy within matching covariate stratum; however, when the parameter of interest is the biomarker's classification accuracy in the general population, ignoring biased sampling can lead to invalid inference (Pepe *and others*, 2012). Inverse probability weighting provides a natural solution to account for biased sampling of cases and/or controls when evaluating marker classification performance in the population, e.g., in Pepe *and others* (2012) for binary disease outcome, and in Cai and Zheng (2011) for failure time outcome. Similar strategies of weighting to estimate classification accuracy under biased sampling have been adopted by other authors in a different problem setting: for correcting verification bias when true disease status (instead of the biomarker) is ascertained only from a subset of subjects (He *and others*, 2009).

However, asymptotic theories for estimators of AUC or difference in AUC for binary disease outcome in two-phase studies where expensive biomarkers are only measured in a subset of the study cohort, are lacking, despite the commonality of AUC in characterizing and comparing diagnostic tests in biomarker research. Our research in this paper aims to fill in this gap. In particular, we will develop inverse-probability-weighted (IPW) AUC estimator in two-phase study designs and develop inference procedures for comparing two diagnostic biomarkers. The closed-form expressions of asymptotic variances we develop for the proposed estimators will be valuable for understanding the implication of frequency matching on efficiency of biomarker evaluation.

This paper will consider two types of two-phase sampling designs: the finite-population stratified sampling (Neyman, 1938) and the Bernoulli sampling (Manski and Lerman, 1977). The former design is commonly used in biomarker research and is the major focus of this paper. The latter design has the advantage of simplicity and will be introduced as a pathway for studying the results in finite-population stratified sampling design. The two designs differ in how individuals are sampled for biomarker measurement in phase two. Finite-population stratified sampling requires pre-specification of a finite number of covariate strata: fixed number of cases and/or controls are then sampled from each stratum. It has the advantage that the number of cases and controls sampled from each stratum in phase two can be fixed at the outset. In Bernoulli sampling, each individual is selected with a known sampling probability that can depend on one's disease status and covariate value, independently of other individuals. It works in more general settings without the need to pre-specify a finite number of strata, e.g., when outcome and/or auxiliary covariates observed in phase one are continuous. The number of cases and/or controls sampled in phase two are random in Bernoulli sampling design. Despite the differences between the two designs, theoretical results of their estimators are closely connected.

In Section 2, we will start with the problem setting in Bernoulli sampling design and propose IPW estimators of AUC and difference in AUC using *estimated* sampling weights. We will then investigate estimation under finite-population stratified sampling design and show the connection in theoretical results of AUC estimators between the two designs. In Section 3, we conduct simulation studies to demonstrate performance of our estimators and subsequent inference procedures, compared with the empirical AUC estimator and hypothesis test ignoring biased sampling. In Section 4, we demonstrate, using a numerical example, the use of the analytical variance formula to guide the optimization of biomarker sampling scheme. The application of our proposed AUC estimators will be demonstrated by data examples in HIV vaccine research and prostate cancer research in Section 5. Concluding remarks are made in Section 6.

## 2. Methods

Let $D$ be a binary outcome of interest to differentiate. In this paper, we consider $D$ to be a binary disease outcome, with value 0 and 1 indicating non-diseased and diseased, respectively. We use subscript $_D$ and $_{\!\bar {D}}$ to indicate case and control, respectively. Let $X$ be a continuous biomarker that is expensive to measure such as a lab assay, assuming that increase in $X$ is associated with increased likelihood of disease. Suppose we have data collected from a two-phase study to evaluate the marker's classification accuracy. In the first phase, subjects' disease status and covariates that are easy to measure such as demographics are collected from a random sample of size $N$ from the target population, with $N_D$ and $N_{\!\bar {D}}$ the number of cases and controls, respectively. In the second phase, the phase-one cohort is further subsampled to measure the biomarker value. Our first objective is to estimate AUC for marker $X$: ${\rm AUC}_x=P(X_D>X_{\!\bar {D}})$. In addition, suppose there is another continuous marker $Y$ measured together with marker $X$. Let ${\rm AUC}_y=P(Y_D>Y_{\!\bar {D}})$. Our second objective is to make inference about the difference in AUC between markers $Y$ and $X$, i.e., $\Delta \,{\rm AUC}={\rm AUC}_y - {\rm AUC}_x$.

### 2.1. Bernoulli sampling

#### 2.1.1. Evaluation of a single marker.

First, consider the standard Bernoulli sampling where in phase two individuals are selected independently of others with pre-specified probabilities. For case $i$ in the phase-one cohort, let $\delta _{Di}$ be the indicator that one's biomarker value is collected in the second phase, with $p_{Di}$ the corresponding sampling probability. Similarly, for control $j$ in the phase-one cohort, let $\delta _{\!\bar {D}_j}$ and $p_{\!\bar {D}j}$ indicate whether he/she is sampled in the second phase and the corresponding sampling probability. Note that $p_{Di}$ and $p_{\!\bar {D}j}$ are individual-specific, whose values can depend on covariate value for case $i$ and control $j$. For example, suppose phase-two sampling probabilities of cases and controls depend on discrete covariate strata: among phase-one samples, cases are allocated into $K_{\!D}$ strata with $N_{Dk_{\!D}}$ cases in stratum $k_{\!D}\in \{1,\ldots , K_{\!D}\}$, and controls are allocated into $K_{\!\bar {D}}$ strata with $N_{\!\bar {D}k_{\!\bar {D}}}$ controls in stratum $k_{\!\bar {D}}\in \{1,\ldots , K_{\!\bar {D}}\}$; in the second phase, cases in stratum $k_{\!D}$ are independently sampled with probability $\pi _{Dk_{\!D}}$ and controls in stratum $k_{\!\bar {D}}$ are independently sampled with probability $\pi _{\!\bar {D}k_{\!\bar {D}}}$. Let $n_{Dk_{\!D}}$ and $n_{\!\bar {D}k_{\!\bar {D}}}$ denote the number of phase-two cases and controls sampled from strata $k_{\!D}$ and $k_{\!\bar {D}}$. They are random numbers with expected values $N_{{D}k_{{D}}}\times \pi _{Dk_{{D}}}$ and $N_{\!\bar {D}k_{\!\bar {D}}}\times \pi _{\!\bar {D}k_{\!\bar {D}}}$, respectively. We have $p_{Di}=\pi _{Dk_{\!D}}$ for case $i$ belonging to stratum $k_{\!D}$ and $p_{\!\bar {D}j}=\pi _{\!\bar {D}k_{\!\bar {D}}}$ for control $j$ belonging to stratum $k_{\!\bar {D}j}$.

For estimation of population AUC, when phase-two case and control samples are representative of their respective populations, standard empirical AUC estimator (Bamber, 1975) $\widehat {{\rm AUC}}_x^{{\rm em}}=\left \{{\sum _{i=1}^{N_{\!D}}\sum _{j=1}^{N_{\!\bar {D}}}\delta _{Di} \delta _{\!\bar {D}j}I(X_{Di}>X_{\!\bar {D}j})}\right \}/\left \{{\sum _{i=1}^{N_{\!D}}\sum _{j=1}^{N_{\!\bar {D}}} \delta _{Di}\delta _{\!\bar {D}j}}\right \}$ using phase-two biomarker data provides a valid estimate. However, when phase-two case and control samples are not representative of their respective populations, $\widehat {{\rm AUC}}_x^{{\rm em}}$ can be severely biased. For example, it is common in biomarker study designs that simple random samples of cases are drawn in the second phase, while controls are matched to cases by covariate strata such that control biomarkers are not representative of their population. To take care of the biased sampling, we propose the use of a weighted estimator of AUC based on the idea of inverse-probability weighting (Horvitz and Thompson, 1952). In particular, we construct IPW versions for the numerator and denominator of the empirical AUC estimator, where the contribution of each participant to a case–control pair is weighted by inverse of the *estimated* sampling probability of the participant in phase two:

(2.1) \begin{equation*} \widehat{{\rm AUC}}_x(\hat{p})= \sum_{i=1}^{N_{\!D}}\sum_{j=1}^{N_{\!\bar{D}}} \frac{\delta_{Di}}{\hat{p}_{Di}}\frac{\delta_{\!\bar{D}j}}{\hat{p}_{\!\bar{D}j}}I(X_{Di} >X_{\!\bar{D}j})\left/\sum_{i=1}^{N_{\!D}}\sum_{j=1}^{N_{\!\bar{D}}}\frac{\delta_{Di}}{\hat{p}_{Di}} \frac{\delta_{\!\bar{D}j}}{\hat{p}_{\!\bar{D}j}},\right. \end{equation*}

where $\hat {p}_{Di}$ and $\hat {p}_{\!\bar {D}j}$ indicate estimated phase-two sampling probabilities for case $i$ and control $j$. For continuous covariate, ${p}_{Di}$ and ${p}_{\!\bar {D}j}$ can be estimated by parametric models such as the logistic regression model. For discrete covariate strata, their empirical estimates can be derived. That is, for a case $i$ belonging to stratum $k_{\!D}$, his/her sampling probability $p_{Di}$ is estimated with $\hat {p}_{Di}=\hat {\pi }_{Dk_{\!D}}=n_{Dk_{\!D}}/N_{Dk_{\!D}}$, the proportion of phase-one cases sampled in phase two from stratum $k_{\!D}$; similarly one can estimate $p_{\!\bar {D}j}$ for control $j$ in stratum $k_{\!\bar {D}}$ with $\hat {p}_{\!\bar {D}j}=\hat {\pi }_{\!\bar {D}k_{\!\bar {D}}} =n_{\!\bar {D}k_{\!\bar {D}}}/N_{\!\bar {D}k_{\!\bar {D}}}$.

In Bernoulli sampling, the true sampling probability for each individual is known, but using “estimated” weight can improve efficiency (Web Appendix A&B, see supplementary material). This has also been recommended in other problem settings such as the weighted likelihood estimators (Robins *and others*, 1994; Breslow and Wellner, 2007). Intuitively it holds because using known sampling weights only involves data for subjects sampled at phase two but estimation of the weights allows incorporation of phase-one data available for all subjects, e.g., the number of phase-one cases/controls in each strata in scenarios where sampling probability in phase two varies across discrete strata.

Suppose we model sampling probabilities of the biomarker among cases and controls separately with finite-dimensional parameters $\theta _{\!D}$ and $\theta _{\!\bar {D}}$. Let $\hat {\theta }_{\!D}$ and $\hat {\theta }_{\!\bar {D}}$ be maximum likelihood estimators. Asymptotic distribution of the IPW AUC estimator based on corresponding estimates of $\hat {p}_{\!D}$ and $\hat {p}_{\!\bar {D}}$ is stated below in Theorem 1 (proof in Web Appendix B, see supplementary material).

**Theorem 1.**

Suppose $N_{\!D}/N\rightarrow \lambda \in (0,1)$ as $N\rightarrow \infty $ and $0< p_{\!D}, p_{\!\bar {D}}\le 1$ for each case and control; then $\sqrt {N}\{\widehat {{\rm AUC}}_x(\hat {p})-{\rm AUC}_x\}$ converges asymptotically to a normal random variable with mean 0 and variance

\begin{align*} \Sigma_{x}&=\frac{1}{\lambda}\times \left[\hbox{var}\{F_{\!\bar{D}x}(X_{\!D})\}+E\left\{\frac{p_{\!D}(1-p_{\!D})}{p_{\!D}^2}F_{\!\bar{D}x}^2(X_{\!D})\right\}\right]\\ &\quad +\frac{1}{1-\lambda}\times \left[\hbox{var}\{S_{{D}x}(X_{\!\bar{D}})\} +E\left\{\frac{p_{\!\bar{D}}(1-p_{\!\bar{D}})}{p_{\!\bar{D}}^2}S_{{D}x}^2(X_{\!\bar{D}})\right\}\right]\\ &\quad -{\rm AUC}_x\times \left(\frac{1}{\lambda}\times \left[E\left\{\left(\frac{1}{p_{\!D}}-1\right) F_{\!\bar{D}x}(X_{\!D})\right\}+\hbox{cov}\left\{F_{\!\bar{D}x}(X_{\!D}),\frac{1}{p_{\!D}}-1\right\}\right]\right.\\ &\quad +\left.\frac{1}{1-\lambda}\times \left[E\left\{\left(\frac{1}{p_{\!\bar{D}}}-1\right) S_{{D}x}(X_{\!\bar{D}})\right\}+\hbox{cov}\left\{S_{Dx}(X_{\!\bar{D}}),\frac{1}{p_{\!\bar{D}}}-1\right\}\right]\right)\\ &\quad -\frac{1}{\lambda}({\rm AUC}_x\times Q_{D}-R_{Dx})^TI_{D}^{-1}({\rm AUC}_x\times Q_{D}-R_{Dx})\\ &\quad -\frac{1}{1-\lambda}({\rm AUC}_x\times Q_{\!\bar{D}}-R_{\!\bar{D}x})^TI_{\!\bar{D}}^{-1}({\rm AUC}_x\times Q_{\!\bar{D}}-R_{\!\bar{D}x}), \end{align*}

where

\[ I_{{D}}=E\left\{\left(\frac{1}{p_{{D}}}+\frac{1}{1-p_{{D}}}\right)\frac{\partial p_{{D}}}{\partial \theta_{{D}k_{\!D}}}\frac{\partial p_{{D}}}{\partial \theta_{{D}k^{\prime}_{\!D}}}\right\},\quad I_{\!\bar{D}}=E\left\{\left(\frac{1}{p_{\!\bar{D}}}+\frac{1}{1-p_{\!\bar{D}}}\right)\frac{\partial p_{\!\bar{D}}}{\partial \theta_{\!\bar{D}k_{\!\bar{D}}}}\frac{\partial p_{\!\bar{D}}}{\partial \theta_{\!\bar{D}k^{\prime}_{\!\bar{D}}}}\right\} \]

are information matrices for estimating $\theta _{\!D}$ and $\theta _{\!\bar {D}}$, $Q_{D}=E(({1}/{p_{\!D}})({\partial p_{\!D}}/{\partial \theta }))$, $R_{Dx}=E \{I(X_{\!D}\,{>}\,X_{\!\bar {D}})({1}/{p_{\!D}})({\partial p_{\!D}}/{\partial \theta _{\!D}})\}$, $Q_{\!\bar {D}}=E(({1}/{p_{\!\bar {D}}})({\partial p_{\!\bar {D}}}/{\partial \theta _{\!\bar {D}}}))$, and $R_{\!\bar {D}x}=E\{I(X_{\!D}>X_{\!\bar {D}})({1}/{p_{\!\bar {D}}}) ({\partial p_{\!\bar {D}}}/{\partial \theta _{\!\bar {D}}})\}$.

#### 2.1.2. Comparison of two markers.

For marker $Y$ measured together with $X$, we can similarly estimate its AUC as $\widehat {{\rm AUC}}_y(\hat {p})=\sum _{i=1}^{N_{\!D}}\sum _{j=1}^{N_{\!\bar {D}}}({\delta _{Di}}/{p_{Di}}) ({\delta _{\!\bar {D}j}}/{p_{\!\bar {D}j}})I(Y_{Di}>Y_{\!\bar {D}j})/\sum _{i=1}^{N_{\!D}} \sum _{j=1}^{N_{\!\bar {D}}}({\delta _{Di}}/{\hat {p}_{Di}})({\delta _{\!\bar {D}j}}/{\hat {p}_{\!\bar {D}j}})$, and estimate the difference in AUC between the two markers with $\Delta \,\widehat {{\rm AUC}}(\hat {p})=\widehat {{\rm AUC}}_y(\hat {p})-\widehat {{\rm AUC}}_x(\hat {p})$. Asymptotic distribution of $\Delta \,\widehat {{\rm AUC}}(\hat {p})$ is shown in Theorem 2 (proof in Web Appendix C, see supplementary material).

**Theorem 2.**

Suppose $N_{\!D}/N\rightarrow \lambda \in (0,1)$ as $N\rightarrow \infty $ and $0<p_{\!D}, p_{\!\bar {D}}\le 1$ for every case and control; then $\sqrt {N}\{\Delta \,\widehat {{\rm AUC}}(\hat {p})-\Delta {\rm AUC}\}$ converges asymptotically to a normal random variable with mean 0 and variance

\begin{align*} \Sigma_{xy}&=\Sigma_{x}+\Sigma_{y}- 2\times \frac{1}{\lambda}\times \left[\hbox{cov}\{F_{\!\bar{D}x}(X_{\!D}),F_{\!\bar{D}y}(Y_{\!D})\} +E\left\{\frac{1-p_{\!D}}{p_{\!D}}F_{\!\bar{D}x}(X_{\!D})F_{\!\bar{D}y}(Y_{\!D})\right\}\right]\\ &\quad -2\times \frac{1}{1-\lambda}\times \left[\hbox{cov}\{S_{{D}x}(X_{\!\bar{D}}),S_{{D}y}(Y_{\!\bar{D}})\} +E\left\{\frac{1-p_{\!\bar{D}}}{p_{\!\bar{D}}}S_{{D}x}(X_{\!\bar{D}})S_{{D}y}(Y_{\!\bar{D}})\right\}\right]\\ &\quad -\Delta\,{\rm AUC}\times \left\{\frac{1}{\lambda}\times \left(E\left[\left(\frac{1}{p_{\!D}}-1\right)\times \left\{F_{\!\bar{D}y}(Y_{\!D})-F_{\!\bar{D}x}(X_{\!D})\right\}\right]\right.\right.\\ &\quad +\left.\hbox{cov}\left\{F_{\!\bar{D}y}(Y_{\!D})-F_{\!\bar{D}x}(X_{\!D}),\frac{1}{p_{\!D}}-1\right\}\right)\\ &\quad +\frac{1}{1-\lambda}\times \left(E\left[\left(\frac{1}{p_{\!\bar{D}}}-1\right)\times \{S_{{D}y}(Y_{\!\bar{D}})-S_{{D}x}(X_{\!\bar{D}})\}\right]\right.\\ &\quad +\left.\left.\hbox{cov}\left\{S_{Dy}(Y_{\!\bar{D}})-S_{{D}x}(X_{\!\bar{D}}),\frac{1}{p_{\!\bar{D}}}-1\right\}\right)\right\}\\ &\quad -\frac{1}{\lambda}\times [\Delta\,{\rm AUC}\times Q_{D}-(R_{Dy}-R_{Dx})]^TI_{\!D}^{-1}[\Delta\,{\rm AUC}\times Q_{D}-(R_{Dy}-R_{Dx})]\\ &\quad -\frac{1}{1-\lambda}\times [\Delta\,{\rm AUC}\times Q_{\!\bar{D}}-(R_{\!\bar{D}y}-R_{\!\bar{D}x})]^TI_{\!\bar{D}}^{-1}[\Delta\,{\rm AUC}\times Q_{\!\bar{D}}-(R_{\!\bar{D}y}-R_{\!\bar{D}x})]. \end{align*}

Previously, many authors have studied inference for comparing AUC between paired markers (Hanley and McNeil, 1983; DeLong *and others*, 1988; Wieand *and others*, 1989; Obuchowski and McClish, 1997). These tests were developed, however, for scenarios where cases and controls are randomly sampled from their respective distributions, and thus are not applicable for settings when there is biased sampling associated with cases and/or controls. In contrast, IPW estimator of $\Delta \,{\rm AUC}$ and its analytical variance as presented in Theorem 2 can be used to construct Wald tests for equal AUC between markers, as will be shown later in simulation studies.

### 2.2. Finite-population stratified sampling

Now we consider the finite-population stratified sampling design, the design commonly used in biomarker studies. Again suppose cases and controls among phase-one samples are allocated into $K_{\!D}$ and $K_{\!\bar {D}}$ strata, respectively, with number $N_{Dk_{\!D}}$ and $N_{\!\bar {D}k_{\!\bar {D}}}$ in each stratum. Fixed numbers of cases $n_{k_{\!D}}$ and controls $n_{k_{\!\bar {D}}}$ are then sampled in phase two from these covariate strata to measure the biomarker $X$. Sampling fractions $n_{Dk_{\!D}}/N_{Dk_{\!D}}$ and $n_{\!\bar {D}k_{\!\bar {D}}}/N_{\!\bar {D}k_{\!\bar {D}}}$ can be random.

Let $W_{\!D}$ be the stratum indicator among cases taking unique values $w_{D1},\ldots , w_{DK_{\!D}}$ and $W_{\!\bar {D}}$ be the stratum indicator among controls taking unique values $w_{\!\bar {D}1},\ldots , w_{\!\bar {D}K_{\!\bar {D}}}$. Compute $\hat {\pi }_{Dk_{\!D}}=n_{D{k_{\!D}}}/N_{Dk_{\!D}}$ and $\hat {\pi }_{\!\bar {D}k_{\!\bar {D}}}=n_{\!\bar {D}k_{\!\bar {D}}}/N_{\!\bar {D}k_{\!\bar {D}}}$. In finite-population stratified sampling, sampling probability of a case or control is constant within their corresponding stratum, i.e., $p_{Di}=\pi _{Dk_{\!D}}$ for case $i$ in stratum $k_{\!D}$ and $p_{\!\bar {D}j}=\pi _{\!\bar {D}k_{\!\bar {D}}}$ for control $j$ in stratum $k_{\!\bar {D}}$. The IPW estimator of AUC for marker $X$ (2.1) can be equivalently represented as

(2.2) \begin{equation*} \widehat{{\rm AUC}}_x(\hat{p}) =\sum_{i=1}^{N_{\!D}}\sum_{j=1}^{N_{\!\bar{D}}} \sum_{k_{\!D}=1}^{K_{\!D}}\sum_{k_{\!\bar{D}}=1}^{K_{\!\bar{D}}} \frac{\delta_{Di}I(W_{Di}=w_{Dk_{\!D}})}{\hat{\pi}_{Dk_{\!D}}} \frac{\delta_{\!\bar{D}j}I(W_{\!\bar{D}j}=w_{\!\bar{D}k_{\!\bar{D}}})}{\hat{\pi}_{\!\bar{D}k_{\!\bar{D}}}} I(X_{Di}>X_{\!\bar{D}j}). \end{equation*}

Suppose, as $N\rightarrow \infty $, sampling fractions for cases among stratum $k_{\!D} \in \{1, \ldots , K_{\!D}\}$ converge with $n_{Dk_{\!D}}/N_{Dk_{\!D}}\rightarrow \pi _{Dk_{\!D}}$, and sampling fractions for controls among stratum $k_{\!\bar {D}} \in \{1, \ldots , K_{\!\bar {D}}\}$ converge with $n_{\!\bar {D}k}/N_{\!\bar {D}k}\rightarrow \pi _{\!\bar {D}k_{\!\bar {D}}}$. Then the asymptotic variance of the IPW AUC estimator (2.2) in finite-population stratified sampling is identical to the asymptotic variance of $\widehat {{\rm AUC}}_x(\hat {p})$ in Bernoulli sampling, if, in phase two of the latter design, cases in stratum $k_{\!D}$ are sampled independently with probability $\pi _{Dk_{\!D}}$ and controls in stratum $k_{\!\bar {D}}$ are sampled independently with probability $\pi _{\!\bar {D}k_{\!\bar {D}}}$, with sampling probabilities estimated empirically. A proof is given in Web Appendix C (see supplementary material). The equality in asymptotic variance of $\Delta \,{\rm AUC}(\hat {p})$ between the two designs can be similarly derived. The same argument on efficiency of weighted likelihood estimators in Cox regression comparing the two designs was made earlier (Breslow and Wellner, 2007).

## 3. Simulation studies

In this section, we conduct simulation studies to investigate performance of the proposed IPW estimators of AUC and $\Delta \,{\rm AUC}$. We consider a binary disease outcome $D$ with prevalence $\lambda =P(D=1)=0.1$ in the population. Let $W^{\ast }$ be a continuous covariate that follows the standard normal distribution among controls $(D=0)$ and $N(0.6,1)$ among cases $(D=1)$. Let $W$ be a discrete covariate stratum derived from $W^{\ast }$ with three levels: $W=1$ if $W^{\ast }<\Phi ^{-1}(1/3)$, $W=2$ if $\Phi ^{-1}(1/3)\le W^{\ast } \le \Phi ^{-1}(2/3)$, and $W=3$ if $W^{\ast }> \Phi ^{-1}(2/3)$, where $\Phi $ is the CDF of the standard normal distribution. We consider two biomarkers $X$ and $Y$, where $(X,Y,W^{\ast })$ are jointly normally distributed conditional on $D$, with $\rho _{xy}$, $\rho _{xw^{\ast }}$, and $\rho _{yw^{\ast }}$ the correlations between $X$ and $Y$, between $X$ and $W^{\ast }$, and between $Y$ and $W^{\ast }$, respectively, conditional on $D$. Among controls, $X$ and $Y$ each follows the standard normal distribution. Among cases, $X$ follows $N(\mu _{Dx},\sigma _{Dx})$ and $Y$ follows $N(1,1)$. The ROC curve based on $X$ and $Y$ individually is thus ${\rm ROC}_x(t)=\Phi \{\mu _{Dx}/\sigma _{Dx}+\Phi ^{-1}(t)/\sigma _{Dx}\}$ with ${\rm AUC}_x=\Phi (\mu _{Dx}/\sqrt {1+\sigma _{Dx}^2})$ and ${\rm ROC}_y(t)=\Phi \left \{1+\Phi ^{-1}(t)\right \}$ with ${\rm AUC}_y=\Phi (1/\sqrt {2})=0.76$.

We generate data from two-phase studies. In the first phase, $N=5000$ subjects are randomly sampled from the population, whose $D$ and $W$ values are measured. In the second phase, we considered both Bernoulli sampling and finite-population stratified sampling of cases and controls for measuring markers $X$ and $Y$, assuming that they are measured on the same set of subjects. In Bernoulli sampling, cases are sampled independently with a constant probability $p_{\rm c}$, and controls are sampled independently with a probability that depends on the stratum $W$. In particular, the sampling probability for a control in stratum $W=w$ equals $p_{\rm c}\times P(W=w,D=1)/P(W=w,D=0)$. This ensures that, on average, biomarkers are measured on equal numbers of cases and controls within each stratum. In finite-population stratified sampling, $n_{\!D}=N\times P(D=1)\times p_{\rm c}$ cases are sampled without replacement, and then within each $W$ stratum, the same number of controls as cases in that stratum are drawn without replacement. This type of sampling design where simple random samples of cases are drawn in the second phase while controls are matched to cases by covariate strata is common in biomarker research. We also investigate other scenarios where both case and control sampling probabilities in phase two vary across strata. The comparative performance of various estimators is similar to the results we will present below (results omitted).

Based on 5000 Monte Carlo simulations in each setting, we evaluate performance of AUC estimators for individual markers. We compute $\widehat {{\rm AUC}}^{{\rm em}}$, and $\widehat {{\rm AUC}}(\hat {p})$ with sampling probabilities estimated empirically among cases and among controls conditional on sampling strata. These estimators are compared with respect to bias, efficiency, coverage of 95% Wald confidence intervals (CIs) based on analytical variance estimates, and the power to test $H_0: {\rm AUC}=0.5$. We also evaluate performance of corresponding estimators for $\Delta \,{\rm AUC}$. We compare Wald tests based on $\Delta \,\widehat {{\rm AUC}}(\hat {p})$ and the common Delong–Delong test (DeLong *and others*, 1988) with respect to type-I error rate and power for testing $H_0:{\rm AUC}_x={\rm AUC}_y$. The latter is implemented in the R package pROC (Robin *and others*, 2011).

### 3.1. Evaluate performance of a single marker

Table 1 gives performance of ${\rm AUC}_x$ estimators for ${\rm AUC}_x \in \{0.5, 0.664, 0.76\}$, with $\sigma _{Dx}\in \{1, 1.5\}$, $\rho _{xw^{\ast }}\in \{0.3, 0.5\}$, and the constant case sampling rate $p_{\rm c}\in \{0.2, 0.5, 0.8\}$, for both Bernoulli sampling and finite-population stratified sampling. For both designs, the empirical AUC estimator is biased (with 5–15% relative bias), while $\widehat {{\rm AUC}}(\hat {p})$ has minimum bias. While negative biases for empirical AUC estimator were observed for the particular simulation settings presented here, in general this estimator can have both positive and negative biases depending on the setting. Coverage of 95% Wald CIs for $\widehat {{\rm AUC}}(\hat {p})$ is close to the nominal level, while the CIs based on the empirical AUC has an undercoverage problem. The Wald test for $H_0: {\rm AUC}_x=0.5$ based on $\widehat {{\rm AUC}}(\hat {p})$ has type-I error close to the nominal level, while the test based on empirical AUC has inflated type-I error, the inflation getting worse with the increase in sample size. The $\widehat {{\rm AUC}}_x(\hat {p})$ estimators in the two different sampling designs have similar variances.

**Table 1.** Performance of different ${\rm AUC}_x$ estimators for the bi-normal marker model described in Section 3. Disease prevalence is $0.1$. Biomarker $X$ is standard normal among controls. By $n_{\!D}$ and $n_{\!\bar {D}},$ we indicate the expected number of cases and controls sampled in phase two for Bernoulli sampling and exact number of cases and controls sampled for finite-population stratified sampling. Results are based on 5000 Monte Carlo simulations

				Bernoulli sampling		FPS$^{\dagger }$ sampling	
$\sigma _{Dx}$	${\rm AUC}_x$	$\rho _{xw^{\ast }}$	$n_{\!D}=n_{\!\bar {D}}$	$\widehat {{\rm AUC}}_x(\hat {p})$	$\widehat {{\rm AUC}}_x^{{\rm em}}$	$\widehat {{\rm AUC}}_x(\hat {p})$	$\widehat {{\rm AUC}}_x^{{\rm em}}$
				${\rm Bias}\times 100$			
1.00	0.50	0.30	100	$-$0.06	$-$3.89	$-$0.05	$-$3.90
			250	0.00	$-$3.85	$-$0.04	$-$3.87
			400	0.01	$-$3.84	$-$0.04	$-$3.89
		0.50	100	$-$0.01	$-$6.51	$-$0.14	$-$6.63
			250	$-$0.03	$-$6.54	$-$0.09	$-$6.58
			400	$-$0.04	$-$6.56	$-$0.04	$-$6.55
1.50	0.50	0.30	100	0.01	$-$2.99	$-$0.06	$-$3.08
			250	$-$0.01	$-$3.03	$-$0.07	$-$3.08
			400	$-$0.04	$-$3.05	$-$0.05	$-$3.05
		0.50	100	0.02	$-$5.05	$-$0.08	$-$5.17
			250	$-$0.03	$-$5.10	$-$0.08	$-$5.14
			400	$-$0.05	$-$5.12	$-$0.03	$-$5.12
1.00	0.664	0.30	100	$-$0.05	$-$3.57	$-$0.05	$-$3.58
			250	0.00	$-$3.54	$-$0.04	$-$3.57
			400	0.01	$-$3.53	$-$0.05	$-$3.59
		0.50	100	$-$0.02	$-$6.01	$-$0.11	$-$6.10
			250	$-$0.02	$-$6.04	$-$0.08	$-$6.08
			400	$-$0.04	$-$6.05	$-$0.05	$-$6.05
1.50	0.664	0.30	100	0.03	$-$2.73	$-$0.06	$-$2.83
			250	0.00	$-$2.77	$-$0.07	$-$2.82
			400	$-$0.03	$-$2.80	$-$0.04	$-$2.80
		0.50	100	0.03	$-$4.63	$-$0.08	$-$4.76
			250	$-$0.01	$-$4.68	$-$0.07	$-$4.74
			400	$-$0.05	$-$4.72	$-$0.02	$-$4.71
1.00	0.76	0.30	100	$-$0.04	$-$3.05	$-$0.05	$-$3.07
			250	0.00	$-$3.03	$-$0.04	$-$3.05
			400	0.01	$-$3.02	$-$0.06	$-$3.07
		0.50	100	$-$0.02	$-$5.14	$-$0.09	$-$5.20
			250	$-$0.02	$-$5.16	$-$0.07	$-$5.20
			400	$-$0.04	$-$5.17	$-$0.05	$-$5.18
1.50	0.76	0.30	100	0.04	$-$2.31	$-$0.06	$-$2.42
			250	0.01	$-$2.36	$-$0.06	$-$2.41
			400	$-$0.03	$-$2.40	$-$0.04	$-$2.39
		0.50	100	0.03	$-$3.95	$-$0.07	$-$4.06
			250	$-$0.01	$-$4.00	$-$0.06	$-$4.05
			400	$-$0.04	$-$4.04	$-$0.02	$-$4.02
				${\rm Var}\times N$			
1.00	0.50	0.30	100	9.68	8.26	9.50	7.70
			250	3.88	3.42	3.74	3.15
			400	2.43	2.16	2.38	2.02
		0.50	100	9.00	8.07	8.62	6.83
			250	3.55	3.31	3.41	2.65
			400	2.17	2.03	2.16	1.66
1.50	0.50	0.30	100	9.72	8.85	9.56	8.16
			250	3.88	3.65	3.79	3.29
			400	2.36	2.18	2.38	2.10
		0.50	100	9.15	8.60	8.72	7.28
			250	3.72	3.58	3.52	2.88
			400	2.24	2.16	2.25	1.82
1.00	0.664	0.30	100	8.03	7.62	8.00	7.21
			250	3.23	3.16	3.14	2.94
			400	2.04	2.01	2.00	1.88
		0.50	100	7.27	7.80	7.05	6.62
			250	2.91	3.21	2.79	2.56
			400	1.79	1.97	1.78	1.62
1.50	0.664	0.30	100	8.15	8.01	8.09	7.39
			250	3.30	3.32	3.23	3.01
			400	2.03	2.01	2.03	1.92
		0.50	100	7.64	8.08	7.35	6.84
			250	3.13	3.36	2.96	2.70
			400	1.92	2.05	1.89	1.71
1.00	0.76	0.30	100	6.04	6.13	6.06	5.85
			250	2.44	2.54	2.38	2.39
			400	1.55	1.63	1.52	1.53
		0.50	100	5.38	6.43	5.32	5.58
			250	2.17	2.66	2.08	2.14
			400	1.34	1.64	1.33	1.35
1.50	0.76	0.30	100	6.17	6.38	6.24	5.97
			250	2.52	2.65	2.47	2.42
			400	1.57	1.62	1.55	1.54
		0.50	100	5.73	6.52	5.69	5.70
			250	2.38	2.74	2.25	2.22
			400	1.48	1.69	1.45	1.41
				Coverage of 95% CI			
1.00	0.50	0.30	100	94.3	83.4	94.1	84.2
			250	94.2	67.9	94.7	67.5
			400	94.5	53.3	94.4	51.6
		0.50	100	94.2	63.9	94.4	63.2
			250	94.6	28.2	95.0	24.4
			400	94.7	10.2	95.0	8.00
1.50	0.50	0.30	100	93.8	88.0	94.1	88.5
			250	93.9	78.0	94.5	78.7
			400	94.5	69.3	94.4	68.7
		0.50	100	94.3	76.3	94.8	77.4
			250	94.5	50.3	95.1	49.7
			400	94.5	31.0	94.9	28.8
1.00	0.664	0.30	100	93.9	86.2	94.0	86.6
			250	94.1	70.6	94.7	70.6
			400	94.2	56.7	94.5	55.3
		0.50	100	94.2	68.6	94.7	68.6
			250	94.8	33.4	95.1	30.0
			400	94.8	13.5	95.1	10.5
1.50	0.664	0.30	100	94.1	90.1	94.2	90.2
			250	94.2	79.9	94.3	81.2
			400	94.5	71.5	94.4	71.1
		0.50	100	94.1	79.8	94.8	81.3
			250	94.3	55.4	94.9	54.8
			400	94.6	35.0	94.8	33.5
1.00	0.76	0.30	100	93.8	88.1	93.7	88.6
			250	94.1	73.8	94.9	73.3
			400	94.3	60.6	94.7	58.8
		0.50	100	94.1	73.3	94.3	73.3
			250	94.9	38.4	95.0	36.2
			400	94.9	18.0	95.0	14.4
1.50	0.76	0.30	100	94.2	91.5	94.1	91.5
			250	94.6	81.7	94.4	83.1
			400	94.2	74.4	94.4	74.5
		0.50	100	94.5	82.9	94.3	84.0
			250	94.1	59.8	95.0	60.0
			400	94.4	39.8	94.7	39.3
				Power for testing $H_0: {\rm AUC}_x=0.5$			
1.00	0.50	0.30	100	5.7	16.6	5.9	15.8
			250	5.8	32.1	5.3	32.5
			400	5.5	46.7	5.6	48.4
		0.50	100	5.8	36.1	5.6	36.8
			250	5.4	71.8	5.0	75.6
			400	5.3	89.8	5.0	92.0
1.50	0.50	0.30	100	6.2	12.0	5.9	11.5
			250	6.1	22.0	5.5	21.3
			400	5.5	30.7	5.6	31.3
		0.50	100	5.7	23.7	5.2	22.6
			250	5.5	49.7	4.9	50.3
			400	5.5	69.0	5.1	71.2
1.00	0.664	0.30	100	96.7	89.2	97.1	90.3
			250	100	99.9	100	99.9
			400	100	100	100	100
		0.50	100	98.3	74.1	98.5	74.5
			250	100	98.2	100	99.1
			400	100	100	100	100
1.50	0.664	0.30	100	97.4	92.0	97.70	92.4
			250	100	100	100	100
			400	100	100	100	100
		0.50	100	98.0	82.3	98.4	83.8
			250	100	99.3	100	99.7
			400	100	100	100	100
1.00	0.76	0.30	100	100	100	100	100
			250	100	100	100	100
		0.50	100	100	100	100	100
			250	100	100	100	100
1.50	0.76	0.30	100	100	100	100	100
			250	100	100	100	100
		0.50	100	100	100	100	100
			250	100	100	100	100

$^{\dagger }$Finite-population stratified sampling.

### 3.2. Compare performance between markers

Table 2 shows performance of $\Delta \,{\rm AUC}$ estimators for both types of sampling designs for settings where $X$ and $Y$ have the same variance and same correlation with $W^{\ast }$ conditional on $D$: $\sigma _{Dx}=1$ and $\rho _{xw^{\ast }}=\rho _{yw^{\ast }}=0.5$, where we have ${\rm AUC}_{x}\in \{0.664, 0.76\}$, $\rho _{xy}\in \{0, 0.5\}$, and $p_{\rm c}\in \{0.2, 0.5, 0.8\}$. The IPW $\Delta \,{\rm AUC}$ estimator has good performance: minimum bias, coverage of 95% CI and type-I error for testing the equivalence in AUC close to nominal level. When markers $X$ and $Y$ have exactly the same distribution (consequently same ROC curve and AUC), the empirical estimator of $\Delta \,{\rm AUC}$ is also unbiased: the biases in ${\rm AUC}_{x}$ and ${\rm AUC}_y$ are equal and thus cancel out, due to the equality in the distribution of the two markers and in their correlation with the matching stratum. The coverage of its 95% CI is close to the nominal level. Type-I error for testing the equivalence in AUC using the Delong–Delong test is also close to the nominal level. When ${\rm AUC}_x=0.664$ and $\Delta \,{\rm AUC}=0.096$, the empirical $\Delta \, {\rm AUC}$ estimator has small bias, with a magnitude much smaller compared with that of the ${\rm AUC}_x$ estimator; its 95% CI has good coverage when sample size is small but slight undercoverage when sample size gets large $(p_{\rm c}=0.8)$. Despite the bias in empirical $\Delta \,{\rm AUC}$, the Delong–Delong test for equivalence in AUC can have advantage in power compared with the IPW estimator in this particular setting, due to the positive bias in AUC difference.

**Table 2.** Performance of various estimators of $\Delta \,{\rm AUC}={\rm AUC}_y-{\rm AUC}_x$ for scenarios where the two markers have same variability and same correlation with covariate $W^{\ast }$ conditional on $D,$ for the bi-normal model described in Section 3. Disease prevalence is $0.1$. Marker $X$ and $Y$ is each standard normal among controls. Here we have $\sigma _{Dx}=\sigma _{Dy}=1,$${\rm AUC}_y=0.76,$$\rho _{xw^{\ast }}=\rho _{yw^{\ast }}=0.5$. By $n_{\!D}$ and $n_{\!\bar {D}},$ we indicate, respectively, the expected number of cases and controls sampled in phase two for Bernoulli sampling and exact number of cases and controls sampled for finite-population stratified sampling. Results are based on 5000 Monte Carlo simulations

				Bernoulli sampling		FPS$^{\dagger }$ sampling	
${\rm AUC}_x$	$\Delta \,{\rm AUC}$	$\rho _{xy}$	$n_{\!D}=n_{\!\bar {D}}$	$\Delta \,\widehat {{\rm AUC}}(\hat {p})$	$\Delta \,\widehat {{\rm AUC}}^{{\rm em}}$	$\Delta \,\widehat {{\rm AUC}}(\hat {p})$	$\Delta \,\widehat {{\rm AUC}}^{{\rm em}}$
				${\rm Bias}\times 100$			
0.76	0.00	0.00	100	0.12	0.11	0.12	0.13
			250	0.03	0.02	0.07	0.09
			400	$-$0.00	$-$0.00	0.05	0.07
		0.50	100	0.08	0.06	$-$0.01	0.01
			250	0.03	0.03	$-$0.02	$-$0.02
			400	$-$0.01	0.01	$-$0.03	$-$0.03
0.664	0.096	0.00	100	0.12	0.98	0.14	1.03
			250	0.03	0.90	0.08	0.97
			400	0.01	0.88	0.05	0.94
		0.50	100	0.11	0.96	0.00	0.90
			250	0.04	0.92	$-$0.01	0.86
			400	$-$0.01	0.89	$-$0.03	0.85
				${\rm Var}\times N$			
0.76	0.00	0.00	100	11.66	13.31	11.94	13.63
			250	4.78	5.48	4.72	5.34
			400	2.94	3.42	2.92	3.36
		0.50	100	6.44	7.26	6.42	7.05
			250	2.51	2.88	2.62	2.91
			400	1.59	1.84	1.62	1.82
0.664	0.096	0.00	100	13.79	14.68	13.92	14.97
			250	5.59	6.02	5.50	5.86
			400	3.42	3.75	3.42	3.69
		0.50	100	7.55	7.96	7.57	7.73
			250	2.94	3.16	3.08	3.21
			400	1.85	2.00	1.91	2.01
				Coverage of 95% CI			
0.76	0.00	0.00	100	94.6	94.8	94.8	95.1
			250	94.7	94.8	94.7	95.0
			400	95.0	94.7	94.7	95.1
		0.50	100	94.8	94.8	94.7	95.0
			250	95.3	94.9	94.6	94.8
			400	95.2	95.0	95.0	95.0
0.664	0.096	0.00	100	94.8	94.4	94.8	94.6
			250	94.3	94.0	94.7	93.8
			400	94.9	93.3	94.8	93.1
		0.50	100	94.6	94.2	94.5	94.5
			250	95.2	93.5	94.4	93.1
			400	95.1	92.4	94.6	92.8
				Power for testing $H_0: {\rm AUC}_x={\rm AUC}_y$			
0.76	0.00	0.00	100	5.4	5.1	5.2	4.8
			250	5.3	5.2	5.3	5.0
			400	5.0	5.2	5.3	4.9
		0.50	100	5.2	5.1	5.3	4.9
			250	4.7	5.1	5.4	5.1
			400	4.8	5.0	5.0	5.0
0.664	0.096	0.00	100	46.7	48.9	46.7	49.4
			250	82.5	85.6	83.3	86.5
			400	95.4	96.6	95.9	96.9
		0.50	100	71.4	75.1	70.7	75.5
			250	97.6	98.8	97.6	98.6
			400	100	100	99.9	100

$^{\dagger }$Finite-population stratified sampling.

Table 3 shows results of $\Delta \,{\rm AUC}$ estimators for settings again with equal variance between $X$ and $Y$ conditional on $D$, i.e., $\sigma _{Dx}=1$. However, unlike Table 2 where both markers have the same correlation with the covariate stratum, here we fix $\rho _{yw^{\ast }}$ to be 0.5 but vary $\rho _{xw^{\ast }}$ from 0.4 to 0.1. When markers $X$ and $Y$ have the same distribution and AUC, the empirical estimator of $\Delta \,{\rm AUC}$ is biased because the magnitude of bias is different between $\widehat {{\rm AUC}}_x^{{\rm em}}$ and $\widehat {{\rm AUC}}_y^{{\rm em}}$ due to the difference in correlation between each marker and the covariate stratum. Bias is also observed when $\Delta \,{\rm AUC}=0.096$. Corresponding 95% CI based on $\Delta \,\widehat {{\rm AUC}}^{{\rm em}}$ has an undercoverage problem. The Delong–Delong test also has an inflated type-I error rate. The inflation gets more severe as the difference between $\rho _{xw^{\ast }}$ and $\rho _{yw^{\ast }}$ increases: type-I error can become similar to power for some settings in Table 3 or become even larger than power in some other constructed settings (details omitted). This is due to the bias in the empirical $\Delta \,{\rm AUC}$ estimator such that the observed difference between two markers equivalent in AUC can appear similar or even larger compared with the observed difference between two markers that differ in AUC. In practice, when difference in correlations between marker and stratification variable exists, its magnitude is likely on the small to medium side, and thus we expect some inflation of type-I error when applying Delong–Delong's test but not extreme. In contrast, the IPW estimator of $\Delta \,{\rm AUC}$ are approximately unbiased with coverage of 95% CIs close to the nominal level; corresponding Wald tests for equivalence in AUC between markers have well-controlled type-I error rates.

**Table 3.** Performance of different estimators of $\Delta \,{\rm AUC}={\rm AUC}_y-{\rm AUC}_x$ for scenarios where the two markers have same variability but different correlation with covariate $W^{\ast }$ conditional on $D,$ for the bi-normal marker model described in Section 3. Disease prevalence is $0.1$. Marker $X$ and $Y$ is each standard normal among controls. Here we have $\sigma _{Dx}=\sigma _{Dy}=1,$${\rm AUC}_y=0.76,$$\rho _{yw^{\ast }}=0.5,$$\rho _{xy}=0.5$. By $n_{\!D}$ and $n_{\!\bar {D}},$ we indicate, respectively, the expected number of cases and controls sampled in phase two for Bernoulli sampling and exact number of cases and controls sampled for finite-population stratified sampling. Results are based on 5000 Monte Carlo simulations

				Bernoulli sampling		FPS$^{\dagger }$ sampling	
${\rm AUC}_x$	$\Delta \,{\rm AUC}$	$\rho _{xw^{\ast }}$	$n_{\!D}=n_{\!\bar {D}}$	$\Delta \,\widehat {{\rm AUC}}(\hat {p})$	$\Delta \,\widehat {{\rm AUC}}^{{\rm em}}$	$\Delta \,\widehat {{\rm AUC}}(\hat {p})$	$\Delta \,\widehat {{\rm AUC}}^{{\rm em}}$
				${\rm Bias}\times 100$			
0.76	0.00	0.40	100	0.07	$-$0.99	0.01	$-$1.02
			250	$-$0.01	$-$1.07	0.03	$-$1.04
			400	$-$0.04	$-$1.09	0.01	$-$1.05
		0.30	100	0.03	$-$2.04	$-$0.03	$-$2.13
			250	$-$0.01	$-$2.10	0.01	$-$2.10
			400	$-$0.01	$-$2.10	0.02	$-$2.09
		0.20	100	0.11	$-$2.98	0.03	$-$3.09
			250	0.04	$-$3.08	0.02	$-$3.09
			400	0.03	$-$3.09	0.01	$-$3.09
		0.10	100	0.11	$-$4.03	0.02	$-$4.10
			250	0.02	$-$4.10	0.04	$-$4.09
			400	0.03	$-$4.11	0.01	$-$4.12
0.664	0.096	0.40	100	0.09	$-$0.29	0.03	$-$0.30
			250	0.01	$-$0.36	0.04	$-$0.33
			400	$-$0.02	$-$0.39	0.02	$-$0.35
		0.30	100	0.03	$-$1.52	$-$0.03	$-$1.62
			250	$-$0.02	$-$1.59	0.01	$-$1.58
			400	$-$0.02	$-$1.59	0.01	$-$1.57
		0.20	100	0.12	$-$2.62	0.03	$-$2.76
			250	0.04	$-$2.74	0.01	$-$2.75
			400	0.02	$-$2.76	0.01	$-$2.75
		0.10	100	0.11	$-$3.85	0.03	$-$3.92
			250	0.01	$-$3.93	0.04	$-$3.92
			400	0.02	$-$3.94	0.01	$-$3.95
				${\rm Var}\times N$			
0.76	0.00	0.40	100	6.63	7.18	6.47	6.98
			250	2.51	2.78	2.53	2.74
			400	1.61	1.77	1.63	1.78
		0.30	100	6.62	6.98	6.63	6.81
			250	2.57	2.75	2.66	2.69
			400	1.62	1.75	1.62	1.67
		0.20	100	6.64	6.90	6.44	6.34
			250	2.56	2.65	2.64	2.57
			400	1.66	1.72	1.62	1.59
		0.10	100	6.75	7.00	6.40	5.88
			250	2.56	2.71	2.51	2.33
			400	1.57	1.66	1.61	1.46
0.664	0.096	0.40	100	7.85	7.92	7.61	7.71
			250	2.97	3.08	2.97	3.04
			400	1.88	1.95	1.92	1.97
		0.30	100	7.92	7.78	7.81	7.66
			250	3.05	3.06	3.14	3.03
			400	1.93	1.96	1.91	1.88
		0.20	100	7.82	7.67	7.70	7.23
			250	3.06	2.98	3.11	2.90
			400	1.96	1.92	1.91	1.80
		0.10	100	7.97	7.81	7.57	6.68
			250	3.02	3.02	2.95	2.63
			400	1.86	1.85	1.90	1.67
				Coverage of 95% CI			
0.76	0.00	0.40	100	94.6	94.1	95.0	94.2
			250	95.0	92.0	95.0	93.2
			400	95.1	90.5	94.4	91.2
		0.30	100	94.5	91.4	94.7	90.7
			250	94.7	85.7	94.8	85.6
			400	94.7	79.1	94.8	79.1
		0.20	100	94.2	86.7	94.7	86.9
			250	95.0	73.6	94.8	74.1
			400	94.2	60.2	94.9	60.7
		0.10	100	94.3	79.4	94.3	81.1
			250	95.1	57.7	94.8	57.4
			400	95.0	38.1	95.1	37.2
0.664	0.096	0.40	100	94.5	95.2	94.7	95.0
			250	95.2	94.8	95.0	94.9
			400	95.3	94.5	94.5	94.4
		0.30	100	94.5	92.6	94.7	92.6
			250	95.0	90.1	94.5	89.8
			400	94.7	86.7	94.6	87.4
		0.20	100	94.1	89.1	94.6	88.9
			250	95.1	80.0	94.5	79.9
			400	94.2	70.3	94.7	70.6
		0.10	100	94.5	82.2	94.2	83.5
			250	94.9	63.3	94.8	64.2
			400	95.0	46.1	94.8	45.7
				Power for testing $H_0: {\rm AUC}_x={\rm AUC}_y$			
0.76	0.00	0.40	100	5.4	5.6	5.0	7.9
			250	5.0	7.9	5.0	6.7
			400	4.9	9.5	5.6	8.8
		0.30	100	5.5	8.5	5.3	14.2
			250	5.3	14.2	5.2	14.3
			400	5.3	20.9	5.2	20.7
		0.20	100	5.8	13.1	5.0	26.3
			250	5.0	26.3	5.2	25.7
			400	5.8	39.6	5.1	39.2
		0.10	100	5.7	20.3	4.9	42.0
			250	4.9	42.0	5.2	42.5
			400	5.0	61.7	4.9	62.6
0.664	0.096	0.40	100	70.2	64.7	97.8	95.8
			250	97.8	95.8	97.9	96.3
			400	99.8	99.6	99.9	99.6
		0.30	100	69.4	54.2	97.5	90.1
			250	97.5	90.1	97.5	90.1
			400	99.8	98.3	99.9	98.6
		0.20	100	70.0	43.1	97.7	79.9
			250	97.7	79.9	97.7	80.3
			400	99.9	94.2	99.8	94.8
		0.10	100	70.6	31.7	97.8	64.4
			250	97.8	64.4	98.1	65.8
			400	99.9	83.8	99.9	84.8

$^{\dagger }$Finite-population stratified sampling.

Table 4 presents results for settings with $\sigma _{Dx}\in \{1.5, 2\}$. That is, the variability of marker $X$ is larger than that of $Y$ among cases. Note that when $\mu _{Dx}=\sqrt {(1+\sigma _{Dx})^2/2}$, we have ${\rm AUC}_x={\rm AUC}_y$, although the two markers can have different ROC curves when $\sigma _{Dx}\ne 1$. When this happens, the Delong–Delong test has an inflated type-I error, even when correlation with the stratification variable is the same for both markers; whereas the Wald test based on the IPW estimator of $\Delta \,{\rm AUC}$ has a well-controlled type-I error rate. For both the scenarios with $\Delta \,{\rm AUC}=0$ and $\Delta \,{\rm AUC}=0.096$, the empirical $\Delta \,{\rm AUC}$ estimator is biased; corresponding 95% CI undercovers the true parameter value. In contrast, the IPW estimators have minimum bias and good coverage of 95% CIs.

**Table 4.** Performance of different estimators of $\Delta \,{\rm AUC}={\rm AUC}_y-{\rm AUC}_x$ for scenarios where the two markers have different variability among cases, for the bi-normal model described in Section 3. Disease prevalence is $0.1$. Marker $X$ and $Y$ are each standard normal among controls. Here we have $\sigma _{Dy}=1,$${\rm AUC}_y=0.76,$$\rho _{yw^{\ast }}=0.5,$$\rho _{xy}=0.5$. By $n_{\!D}$ and $n_{\!\bar {D}},$ we indicate, respectively, the expected number of cases and controls sampled in phase two for Bernoulli sampling and exact number of cases and controls sampled for finite-population stratified sampling. Results are based on 5000 Monte Carlo simulations

					Bernoulli sampling		FPS$^{\dagger }$ sampling	
$\sigma _{Dx}$	${\rm AUC}_x$	$\Delta \,{\rm AUC}$	$\rho _{xw^{\ast }}$	$n_{\!D}=n_{\!\bar {D}}$	$\Delta \,\widehat {{\rm AUC}}(\hat {p})$	$\Delta \,\widehat {{\rm AUC}}^{{\rm em}}$	$\Delta \,\widehat {{\rm AUC}}(\hat {p})$	$\Delta \,\widehat {{\rm AUC}}^{{\rm em}}$
					${\rm Bias}\times 100$			
1.50	0.76	0.00	0.50	100	0.04	$-$1.11	0.04	$-$1.08
				250	0.01	$-$1.13	$-$0.01	$-$1.15
				400	0.01	$-$1.12	$-$0.02	$-$1.16
			0.30	100	0.04	$-$2.69	$-$0.03	$-$2.79
				250	0.01	$-$2.75	$-$0.01	$-$2.77
				400	$-$0.01	$-$2.76	$-$0.00	$-$2.76
2.00	0.76	0.00	0.50	100	$-$0.07	$-$2.02	0.10	$-$1.83
				250	$-$0.02	$-$1.95	0.02	$-$1.91
				400	0.02	$-$1.90	$-$0.00	$-$1.93
			0.30	100	0.01	$-$3.19	$-$0.04	$-$3.27
				250	$-$0.00	$-$3.22	$-$0.01	$-$3.23
				400	$-$0.00	$-$3.21	$-$0.02	$-$3.24
1.50	0.664	0.096	0.50	100	0.05	$-$0.43	0.05	$-$0.39
				250	0.01	$-$0.45	0.00	$-$0.46
				400	0.01	$-$0.44	$-$0.01	$-$0.47
			0.30	100	0.04	$-$2.29	$-$0.02	$-$2.38
				250	$-$0.00	$-$2.35	0.00	$-$2.35
				400	$-$0.01	$-$2.35	0.01	$-$2.35
2.00	0.664	0.096	0.50	100	$-$0.04	$-$1.46	0.11	$-$1.27
				250	$-$0.02	$-$1.40	0.03	$-$1.36
				400	0.03	$-$1.35	0.00	$-$1.37
			0.30	100	0.01	$-$2.86	$-$0.03	$-$2.93
				250	$-$0.01	$-$2.90	$-$0.00	$-$2.90
				400	$-$0.00	$-$2.89	$-$0.01	$-$2.90
					${\rm Var}\times N$			
1.50	0.76	0.00	0.50	100	6.84	7.62	6.57	7.29
				250	2.69	3.05	2.71	3.01
				400	1.67	1.90	1.70	1.92
			0.30	100	6.92	7.55	6.85	7.26
				250	2.67	2.94	2.68	2.81
				400	1.67	1.84	1.67	1.77
2.00	0.76	0.00	0.50	100	7.27	7.98	6.91	7.82
				250	2.89	3.21	2.82	3.17
				400	1.81	2.00	1.77	2.01
			0.30	100	7.32	8.07	7.20	7.73
				250	2.87	3.17	2.85	3.03
				400	1.79	1.98	1.79	1.92
1.50	0.664	0.096	0.50	100	7.93	8.39	7.68	8.05
				250	3.14	3.37	3.17	3.35
				400	1.91	2.07	1.98	2.12
			0.30	100	8.07	8.36	7.98	8.13
				250	3.14	3.27	3.12	3.15
				400	1.95	2.04	1.95	1.98
2.00	0.664	0.096	0.50	100	8.47	8.92	8.03	8.71
				250	3.34	3.57	3.28	3.56
				400	2.05	2.18	2.06	2.24
			0.30	100	8.44	8.96	8.26	8.63
				250	3.33	3.52	3.28	3.39
				400	2.07	2.18	2.06	2.14
					Coverage of 95% CI			
1.50	0.76	0.00	0.50	100	94.8	93.6	95.0	94.1
				250	94.6	91.9	94.6	92.2
				400	94.6	90.5	94.7	90.3
			0.30	100	94.2	88.0	94.3	88.1
				250	94.8	78.8	94.8	78.5
				400	94.6	68.3	94.9	68.4
2.00	0.76	0.00	0.50	100	94.7	91.4	95.3	92.3
				250	94.8	87.8	94.6	86.9
				400	94.7	83.5	94.4	83.5
			0.30	100	94.3	86.4	94.6	85.6
				250	94.4	73.6	94.6	73.7
				400	94.5	61.8	94.6	60.9
1.50	0.664	0.096	0.50	100	94.7	94.7	94.7	95.0
				250	94.8	94.0	94.3	94.2
				400	94.9	93.9	94.4	93.6
			0.30	100	94.3	90.3	94.4	89.7
				250	94.6	84.2	94.9	84.1
				400	94.5	77.4	94.8	77.0
2.00	0.664	0.096	0.50	100	94.3	93.0	95.1	93.7
				250	94.5	91.2	94.8	91.0
				400	94.7	89.5	94.5	89.2
			0.30	100	94.6	88.7	94.6	87.9
				250	94.3	79.3	94.5	79.3
				400	94.7	70.2	94.6	70.0
					Power for testing $H_0: {\rm AUC}_x={\rm AUC}_y$			
1.50	0.76	0.00	0.50	100	5.2	6.3	5.0	5.8
				250	5.4	8.0	5.4	7.7
				400	5.4	9.5	5.3	9.6
			0.30	100	5.8	11.8	5.7	11.8
				250	5.2	21.1	5.2	21.3
				400	5.4	31.6	5.1	31.5
2.00	0.76	0.00	0.50	100	5.3	8.5	4.70	7.5
				250	5.2	12.1	5.4	13.0
				400	5.3	16.4	5.6	16.5
			0.30	100	5.7	13.4	5.4	14.2
				250	5.6	26.3	5.4	26.2
				400	5.5	38.1	5.4	39.0
1.50	0.664	0.096	0.50	100	68.9	61.1	69.9	62.3
				250	97.0	94.8	97.1	94.6
				400	99.9	99.6	99.8	99.3
			0.30	100	68.6	44.4	69.6	44.4
				250	97.1	81.9	97.6	82.0
				400	99.9	95.2	99.9	95.4
2.00	0.664	0.096	0.50	100	66.1	49.1	67.9	50.9
				250	96.2	87.5	96.6	87.6
				400	99.8	97.6	99.6	97.6
			0.30	100	66.4	37.4	66.9	37.2
				250	96.3	72.2	96.9	73.0
				400	99.8	89.9	99.9	89.9

$^{\dagger }$Finite-population stratified sampling.

In Web Appendix H (see supplementary material), we also present additional simulation results when biomarkers follow gamma distributions conditional on disease status. The conclusion regarding the performance of the IPW and the empirical AUC estimators is similar to the bi-normal marker model.

## 4. Implication on efficiency of sampling schemes

The analytical variance formula we developed in Section 2 will be valuable to biomarker researchers for studying the impact of the sampling scheme on the efficiency of biomarker performance estimators. We demonstrate that using an example comparing two designs with the same number of participants measuring disease outcome, covariate, and biomarker. The setting is similar to that in Section 3, with disease prevalence $\lambda =P(D=1)=0.1$. Covariate $W^{\ast }$ and biomarker $X$ are bivariate normal with correlation $\rho _{xw^{\ast }}$ conditional on $D$. Among controls $X$ and $W^{\ast }$ are each standard normal; among cases $W^{\ast }\sim N(0.6,1)$ and $X\sim N(\mu _{Dx},1)$. Let $W$ be a binary covariate stratum derived from $W^{\ast }$, with $W=1$ if $W^{\ast }\le \Phi ^{-1}(1/2),$ and $W=2$ otherwise. We compare two sampling designs. Both are two-phase studies with a random cohort sample of size $N$ drawn in the first phase. In the second phase, both designs include all cases from a phase-one sample, i.e., $\pi _{\!D}=1$; a simple random sample of controls of size $n_{\!\bar {D}}=n_{\!D}$ are drawn without replacement in Design 1, simple random samples of controls with the same number as cases are drawn without replacement from each $W$ stratum in Design 2. In Design 1, phase-two case and control samples are representative of their respective distributions; thus the empirical estimator of AUC is valid and is considered for this design. In contrast, phase-two case and control samples in Design 2 are not representative of their respective distributions; thus we use the proposed $\widehat {{\rm AUC}}(\hat {p})$ with empirically estimated sampling weights conditional on sampling strata for Design 2.

Following Theorem 1 and Section 2.4, asymptotic variance of $\widehat {{\rm AUC}}(\hat {p})$ in Design 2 equals

(4.1) \begin{equation*} \frac{\hbox{var}\{F_{\!\bar{D}x}(X_{\!D})\}}{N\times \lambda}+\frac{1}{N(1-\lambda)}\left\{\hbox{var}\{S_{{D}x}(X_{\!\bar{D}})\}+ \sum_{k_{\!\bar{D}}=1}^2\hbox{var}\{S_{{D}x}(X_{\!\bar{D}k_{\!\bar{D}}})\}P(W_{\!\bar{D}} =w_{\!\bar{D}k_{\!\bar{D}}})\frac{1-\pi_{\!\bar{D}k_{\!\bar{D}}}}{\pi_{\!\bar{D}k_{\!\bar{D}}}}\right\}. \end{equation*}

Since $\widehat {{\rm AUC}}^{{\rm em}}$ in Design 1 can be thought of as an $\widehat {{\rm AUC}}(\hat {p})$ estimator where there is only one sampling stratum for cases and for controls, its asymptotic variance can be similarly derived as

(4.2) \begin{equation*} \frac{\hbox{var}\{F_{\!\bar{D}x}(X_{\!D})\}}{N\times \lambda}+\frac{1}{N\times (1-\lambda)}\left\{\hbox{var}\{S_{{D}x}(X_{\!\bar{D}})\}+\hbox{var}\{S_{{D}x}(X_{\!\bar{D}})\} \times\frac{1-\pi_{\!\bar{D}}}{\pi_{\!\bar{D}}}\right\}, \end{equation*}

for $\pi _{\!\bar {D}}=\pi _{\!D}\times \lambda /(1-\lambda )$. The result also follows DeLong *and others* (1988).

**Fig. 1.**
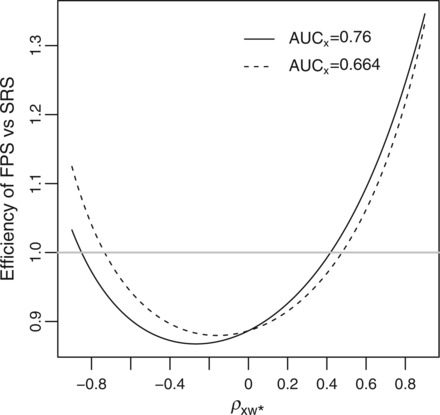
Efficiency of $\widehat {{\rm AUC}}(\hat {p})$ in finite-population stratified sampling (FPS) of controls relative to the empirical AUC estimator in simple random sampling (SRS) without replacement of controls. Efficiency = asymptotic variance of empirical AUC estimator in SRS (equation (4.2))/asymptotic variance of $\widehat {{\rm AUC}}(\hat {p})$ in FPS (equation (4.1)).

Figure 1 shows the relative asymptotic efficiency of $\widehat {{\rm AUC}}(\hat {p})$ in Design 2 versus $\widehat {{\rm AUC}}^{{\rm em}}$ in Design 1 for two different ${\rm AUC}_{x}$ values, as $\rho _{xw^{\ast }}$ changes from $-$0.9 to 0.9. A common U-shape is observed, where Design 2 is more efficient as the magnitude of the correlation increases, whereas Design 1 can be more efficient when the correlation is small. Comparing variance formulae of the two estimators, their difference arises from two components: (i) the difference between $(1-\pi _{\!\bar {D}})/\pi _{\!\bar {D}}$ and $1-\pi _{\!\bar {D}k_{\!\bar {D}}}/\pi _{\!\bar {D}k_{\!\bar {D}}}$, and (ii) the difference between $\hbox {var}\{S_{Dx}(X_{\!\bar {D}})\}$ and $\hbox {var}\{S_{Dx}(X_{\!\bar {D}k_{\!D}})\}$. Note that when $X$ and $W^{\ast }$ are not correlated among controls, the difference in variance between the two estimators is solely due to component (i): simple random sampling without replacement from controls is more efficient since the weighted average of $1-\pi _{\!\bar {D}k_{\!\bar {D}}}/\pi _{\!\bar {D}k_{\!\bar {D}}}$ for $k_{\!\bar {D}}=1,2$ is larger than $(1-\pi _{\!\bar {D}})/\pi _{\!\bar {D}}$ in our example. As $X$ and $W^{\ast }$ become more correlated, variance of $S_{Dx}(X_{\!\bar {D}})$ conditional on the covariate stratum becomes smaller compared with its variance among all controls, and thus stratified sampling is more efficient. This is consistent with the use of stratified sampling in survey sampling as a possible way to increase efficiency in estimating parameters such as the population total when a heterogeneous population can be divided into strata with homogeneous units (Cochran, 2007). The same pattern can be observed when biomarkers conditional on disease status follow gamma distributions (Web Supplementary Figure S3). In practice, researchers can evaluate efficiency of different sampling schemes based on prior knowledge in the relationship between marker, covariate, and disease.

## 5. Example

### 5.1. RV144 example

We illustrate the proposed methodology for estimating and comparing AUC with a real example of biomarker study from the RV144 Thailand HIV vaccine trial. The trial included 16 402 participants aged 18–30 who were 1:1 randomized into a vaccine and a placebo arm. Among vaccine recipients in the RV144 trial who were not yet infected at week 26, an immune response study was conducted to assess vaccine-induced immune response based on peak immunogenicity at week 26 following a finite-population stratified sampling design (Haynes *and others*, 2012). Around 1.8% vaccinees were censored before the end of the study and were treated as non-infected for subsequent sampling. The study includes all 41 vaccinees infected after week 26 visits. The control vaccinees were selected from a stratified random sample of vaccinees free of HIV-1 infection at 42 months, within strata constructed by gender, number of vaccinations received, and per-protocol status, with five times the number of cases within each stratum.

Two of the primary assays studied, the binding of IgG antibodies to variable regions 1 and 2 (V1V2) of the gp120 Env, and the binding of plasma IgA antibodies to Env, were found to correlate significantly with infection risk (Haynes *and others*, 2012). Here we evaluate and compare AUC of the two markers. In this application, $D$ will be HIV infection at 42 months, and $X$ and $Y$ are V1V2 and IgA measures at week 26, respectively.

First, the naive empirical AUC estimate is 0.573 (95% ${\rm CI}=(0.483, 0.663)$) for V1V2, and 0.596 (95% ${\rm CI}=(0.499,0.693)$) for IgA. There is around 4% relative increase in AUC for IgA compared with V1V2, although the difference is not statistically significant ($p$-value $=$ 0.774 based on the Delong–Delong test). With empirically estimated sampling weights conditional on the matching stratum, the $\widehat {{\rm AUC}}(\hat {p})$ equals 0.588 (95% ${\rm CI}=(0.498, 0.679)$) for V1V2 and 0.589 (95% ${\rm CI}=(0.490, 0.687)$) for IgA. The difference in AUC between the two markers becomes even smaller after accounting for the sampling scheme with a $p$-value of 0.997 based on the Wald test. In this example, adjusting for the sampling scheme makes a small difference in point estimates due to the relatively small variability in sampling weights of controls across strata; the observation that the IPW estimate of the AUC difference between IgA and V1V2 is smaller than the empirical estimate is consistent with observations made in simulation studies, where bias in $\Delta \,\widehat {{\rm AUC}}^{{\rm em}}$ can make two markers look more different when they have equal AUC.

### 5.2. Prostate cancer study example

In the second example, we consider a prospective study conducted by the Early Detection Research Network aimed to assess a urine biomarker for prostate cancer, the Prostate Cancer Antigen 3 (PCA3) (Deras *and others*, 2008). This study involved 570 men enrolled at four North American sites scheduled for prostate biopsy, with a prostate cancer $(D)$ prevalence of 36.6%. Urinary PCA3 $(Y)$ and serum PSA (prostate-specific agent, $X$) are obtained from every participant using specimens collected before biopsy. Each biomarker is log-transformed and standardized to have mean zero and variance 1 among subjects without prostate cancer. Among those with prostate cancer, PCA3 and PSA have mean 0.64 and 0.42, and variance 1.0 and 0.83, respectively. Pearson correlations with age are 0.40 for PCA3 and 0.15 for PSA among those without prostate cancer, and 0.24 for PCA3 and 0.28 for PSA among those with prostate cancer. Increase in age also appears to be associated with increased risk of prostate cancer.

To illustrate application of our methodology, we perform a finite-population stratified sampling based on age strata generated by the first and second tertiles of age distribution among controls, i.e., age $<$60, 60–67, $>$67 years. We randomly sampled 120 cases and then sampled the same number of controls as cases within each age stratum. Based on the case–control sample, the empirical AUC estimate is 0.553 (95% ${\rm CI}=(0.481, 0.626)$) for PSA and 0.645 (95% ${\rm CI}=(0.576,0.715)$) for PCA3, with a difference not statistically significant ($\Delta \,{\rm AUC}=0.092$ with 95% CI ($-$0.004, 0.188), and $p$-value $=$ 0.061 based on the Delong–Delong test). In contrast, $\widehat {{\rm AUC}}(\hat {p})$ with empirically estimated sampling weights conditional on the stratum equals 0.553 (95% ${\rm CI}=(0.481, 0.626)$) for PSA and 0.671 (95% ${\rm CI}=(0.606, 0.736)$) for PCA3, with a significant difference detected ($\Delta \,{\rm AUC} = 0.111$ with 95% CI (0.017, 0.206), $p$-value $=$ 0.021 based on the Wald test). Note that there is a significant difference between the two markers if we estimate the empirical AUC based on the full cohort sample ($\Delta \,{\rm AUC} = 0.117$, 95% ${\rm CI} = (0.054,0.179)$, $p$-value $=$ 0.0003 based on Delong–Delong's test). Thus, with the case–control sample only, we would have identified the significant difference with the weighted estimator, but have missed it using the empirical estimator.

## 6. Concluding remarks

In this paper, we developed methods for assessing a biomarker's classification accuracy for a binary disease as characterized by the area under the ROC curve in two-phase sampling designs. Finite-population stratified sampling of biomarkers from a phase-one cohort representative of the target population has become increasingly common in recent years with the availability of large clinical trials and cohort studies. But statistical methods to properly handle the design components when assessing biomarkers are not yet well developed. Empirical area under the ROC curve estimated from the phase-two biomarker samples was often reported in applied literatures even in the presence of biased sampling of cases and/or controls. We showed in this paper that empirical estimators of AUC ignoring the biased sampling scheme can lead to severely biased estimates of classification accuracy and invalid inference for comparing biomarkers. We investigated an inverse-sampling-probability-weighted estimator that achieves unbiased estimation of AUC and developed asymptotic variance formulae applicable to inference in finite-population stratified sampling, through its connection with the IPW estimator in the standard Bernoulli sampling design. The analytical variance formulae we developed will provide valuable guidance to biomarker researchers on optimizing the sampling scheme in designing future biomarker studies, in order to achieve better efficiency in evaluating biomarkers for classification accuracy. For Bernoulli sampling, we observed analytically and numerically that using estimated sampling weights is more efficient even when the true sampling weights are known by design (Web Supplementary Appendix A–C, F–H, see supplementary material). In particular, using estimated weights can lead to much improved efficiency for estimating performance of a single marker. In contrast, the improvement due to weights estimation is relatively minor for comparing performance of paired markers; when sample size is small, the CI of the $\Delta \,{\rm AUC}$ estimator based on known sampling weights can have slightly better coverage than that based on estimated weights.

In this paper, we considered simple design-based weights, the estimation of which does not require more information beyond the sampling strata. In etiology studies, it has been shown that when auxiliary variables are available from a phase-one cohort, they can be used to further adjust the weights for potential efficiency gains in estimating the disease odds ratio or hazard ratio (Breslow *and others*, 2009). It is interesting in future work to investigate the impact of weight adjustments using auxiliary variables on biomarker performance measure such as the AUC. Finally, the inverse-probability-weighting methods can be naturally applied to other performance measures such as the points on the ROC curve and the partial area under the ROC curve.

## Supplementary material

Supplementary Material is available here.

## Funding

This work was supported by the U.S. National Institutes of Health grants R01 GM106177-01 and R01 GM54438. Funding to pay the Open Access publication charges for this article was provided by NIH grant R01 106177.

## Supplementary Material

Supplementary Data
