# Supplementary material for: Evaluating and comparing biomarkers with respect to the area under the receiver operating characteristics curve in two-phase case–control studies
Source: Biostatistics. 2016 Feb 16;17(3):499–522. doi: 10.1093/biostatistics/kxw003 (PMC4915610; doi:10.1093/biostatistics/kxw003)
Supplement: Supplementary Data [file kxw003supp.pdf]

Biostatistics (2016), **0**, 0, *pp.* 1–43
doi:10.1093/biostatistics/WebSupplementary˙BIOSTS-15073-R3New

Supplementary Material for "Evaluating and Comparing Biomarkers with respect to the Area under the Receiver Operating Characteristics Curve in Two-Phase Case-Control Studies"

YING HUANG*
Fred Hutchinson Cancer Research Center, Seattle, Washington, 98109, U.S.A.
Department of Biostatistics, University of Washington, Seattle, Washington, 98109, U.S.A.
yhuang@fhcrc.org

Appendix A: Alternative AUC estimators in Bernoulli Sampling

For case i in the phase-one cohort, let δ_{Di} be the indicator that one's biomarker value is collected in the second phase, with p_{D_i} the corresponding sampling probability. Similarly, for control j in the phase-one cohort, let $\delta_{\bar{D}_j}$ and $p_{\bar{D}j}$ indicate whether he/she is sampled in the second phase and the corresponding sampling probability. To prove asymptotic normality of $\widehat{AUC}_x(\hat{p})$ in Bernoulli sampling design, we first describe three other inverse-probability-weighted (IPW) AUC estimator.

(I) First, hypothetically if we have the biomarker X measured for the entire phase-one cohort, an unbiased estimate of AUC_x can be generated as $\sum_{i=1}^{N_D}\sum_{j=1}^{N_{\bar{D}}} I(X_{Di} > X_{\bar{D}j})/(N_D N_{\bar{D}})$. To account for the sub-sampling of cases and controls in the second phase, we can construct an IPW AUC estimator where the contribution of each participant to a case-control pair is weighted by the known sampling probability of the participant in phase two:

$$\widetilde{AUC}_x(p) = \frac{1}{N_D}\frac{1}{N_{\bar{D}}}\sum_{i=1}^{N_D}\sum_{j=1}^{N_{\bar{D}}}\frac{\delta_{Di}}{p_{Di}}\frac{\delta_{\bar{D}j}}{p_{\bar{D}j}} I(X_{Di} > X_{\bar{D}j}). \tag{0.1}$$

*To whom correspondence should be addressed.

© The Author 2016. Published by Oxford University Press. All rights reserved. For permissions, please e-mail: journals.permissions@oup.com

(II) Second, note that an IPW estimator of $N_D N_{\bar{D}}$, the total number of case-control pairs in the phase-one sample, can be constructed as $\sum_{i=1}^{N_D}\sum_{j=1}^{N_{\bar{D}}} \delta_{Di}\delta_{\bar{D}j}/(p_{Di}p_{\bar{D}j})$. Therefore, one could replace $N_D N_{\bar{D}}$ in (0.1) with its IPW estimator. This leads to an alternative AUC estimator

$$\widehat{AUC}_x(p) = \sum_{i=1}^{N_D}\sum_{j=1}^{N_{\bar{D}}} \frac{\delta_{Di}}{p_{Di}}\frac{\delta_{\bar{D}j}}{p_{\bar{D}j}} I(X_{Di} > X_{\bar{D}j}) / \sum_{i=1}^{N_D}\sum_{j=1}^{N_{\bar{D}}} \frac{\delta_{Di}}{p_{Di}}\frac{\delta_{\bar{D}j}}{p_{\bar{D}j}}. \tag{0.2}$$

An alternative way to view this is that $\widehat{AUC}_x(p) = \sum_{i=1}^{N_D}\sum_{j=1}^{N_{\bar{D}}} \delta_{Di}v_{Di}\delta_{\bar{D}j}v_{\bar{D}j} I(X_{Di} > X_{\bar{D}j})/(N_D N_{\bar{D}})$ where v_{Di} and $v_{\bar{D}j}$ are weights assigned to case i and control j respectively, with $v_{Di} = N_D/\left(p_{Di}\sum_{k=1}^{N_D}\delta_{Dk}/p_{Dk}\right)$ and $v_{\bar{D}j} = N_{\bar{D}}/\left(p_{\bar{D}j}\sum_{k=1}^{N_{\bar{D}}}\delta_{\bar{D}k}/p_{\bar{D}k}\right)$. Since $\sum_{i=1}^{N_D}\delta_{Di}v_{Di} = N_D$ and $\sum_{j=1}^{N_{\bar{D}}}\delta_{\bar{D}j}v_{\bar{D}j} = N_{\bar{D}}$, $\widehat{AUC}(p)$ can be thought as the IPW estimator where weights are calibrated such that sample sizes for phase-one cases or controls equal to their IPW estimates based on phase-two sample.

(III) The third AUC estimator replaces known sampling probability in $\widetilde{AUC}(p)$ with their estimates. Let $\hat{p}_D$ and $\hat{p}_{\bar{D}}$ indicate estimated phase-two sampling probabilities for cases and controls respectively: $\widetilde{AUC}_x(\hat{p}) = \frac{1}{N_D}\frac{1}{N_{\bar{D}}}\sum_{i=1}^{N_D}\sum_{j=1}^{N_{\bar{D}}} \frac{\delta_{Di}}{\hat{p}_{Di}}\frac{\delta_{\bar{D}j}}{\hat{p}_{\bar{D}j}} I(X_{Di} > X_{\bar{D}j}).$ (0.3)

Finally, note the $\widehat{AUC}_x(\hat{p})$ as presented in (2.1) of main paper replaces known sampling probability in $\widehat{AUC}(p)$ with their estimated sampling probabilities.

The four AUC estimators: $\widetilde{AUC}_x(p)$, $\widehat{AUC}_x(p)$, $\widetilde{AUC}_x(\hat{p})$, and $\widehat{AUC}_x(\hat{p})$ in general differ from each other. In the special case where the phase-two sampling probabilities for cases and controls are constant within each covariate stratum with p_D and $p_{\bar{D}}$ estimated empirically within discrete covariate strata, $\widetilde{AUC}(\hat{p})$ in (0.3) and $\widehat{AUC}(\hat{p})$ in (2.1) of main paper are equivalent as shown below. Moreover, if in phase two cases and controls are each randomly sampled, then $\widetilde{AUC}(\hat{p})$, $\widehat{AUC}(p)$, and $\widehat{AUC}(\hat{p})$ are equivalent to the empirical AUC estimator.

Let W_D and $W_{\bar{D}}$ indicate the discrete stratum for sampling the biomarker, for a case and a control respectively. The strata defined can be same or different between cases and controls. Suppose there are K_D strata for cases and $K_{\bar{D}}$ strata for controls such that W_D can take unique values $\{w_{D1}, \ldots, w_{DK_D}\}$ and $W_{\bar{D}}$ can take unique values $\{w_{\bar{D}1}, \ldots, w_{\bar{D}K_{\bar{D}}}\}$. Let

N_{Dk_D} and $N_{\bar{D}k_{\bar{D}}}$ be the number of phase-one cases and controls in stratum k_D and $k_{\bar{D}}$ respectively. Let n_{Dk_D} and $n_{\bar{D}k_{\bar{D}}}$ be the number of phase-two cases and controls in stratum k_D and $k_{\bar{D}}$ respectively. Let $\pi_D = \{\pi_{D1}, \ldots, \pi_{DK_D}\}$ and $\pi_{\bar{D}} = \{\pi_{\bar{D}1}, \ldots, \pi_{\bar{D}K_{\bar{D}}}\}$ be phase-two sampling probabilties of cases and controls within each stratum, with empirical estimates $\hat{\pi}_{Dk_D} = \sum_{i=1}^{N_D} \delta_{Di} I(W_{Di} = w_{Dk_D}) / \sum_{i=1}^{N_D} I(W_{Di} = w_{Dk_D}) = n_{Dk_D}/N_{Dk_D}$, $\hat{\pi}_{\bar{D}k_{\bar{D}}} = \sum_{j=1}^{N_{\bar{D}}} \delta_{\bar{D}j} I(W_{\bar{D}j} = w_{\bar{D}k_{\bar{D}}}) / \sum_{j=1}^{N_{\bar{D}}} I(W_{\bar{D}j} = w_{\bar{D}k_{\bar{D}}}) = n_{\bar{D}k_{\bar{D}}}/N_{\bar{D}k_{\bar{D}}}$. Then we have

$$\frac{1}{N_D}\frac{1}{N_{\bar{D}}}\sum_{i=1}^{N_D}\sum_{j=1}^{N_{\bar{D}}}\frac{\delta_{Di}}{\hat{p}_{Di}}\frac{\delta_{\bar{D}j}}{\hat{p}_{\bar{D}j}} = \sum_{i=1}^{N_D}\sum_{j=1}^{N_{\bar{D}}}\sum_{k_D=1}^{K_D}\sum_{k_{\bar{D}}=1}^{K_{\bar{D}}} \frac{\delta_{Di} I(W_{Di} = w_{Dk_D}) N_{Dk_D}}{n_{Dk_D}} \frac{\delta_{\bar{D}j} I(W_{\bar{D}j} = w_{\bar{D}k_{\bar{D}}}) N_{\bar{D}k_{\bar{D}}}}{n_{\bar{D}k_{\bar{D}}}}$$

$$= \sum_{k_D=1}^{K_D}\sum_{k_{\bar{D}}=1}^{K_{\bar{D}}} \frac{\sum_{i=1}^{N_D}\sum_{j=1}^{N_{\bar{D}}} \delta_{Di}\delta_{\bar{D}j} I(W_{Di} = w_{Dk_D}) I(W_{\bar{D}j} = w_{\bar{D}k_{\bar{D}}})}{n_{Dk_D} n_{\bar{D}k_{\bar{D}}}} \frac{N_{Dk_D} N_{\bar{D}k_{\bar{D}}}}{N_D N_{\bar{D}}}$$

$$= \sum_{k_D=1}^{K_D}\sum_{k_{\bar{D}}=1}^{K_{\bar{D}}} \frac{\sum_{i=1}^{N_D}\sum_{j=1}^{N_{\bar{D}}} \delta_{Di}\delta_{\bar{D}j} I(W_{Di} = w_{Dk_D}) I(W_{\bar{D}j} = w_{\bar{D}k_{\bar{D}}})}{\sum_{i=1}^{N_D}\sum_{j=1}^{N_{\bar{D}}} \delta_{Di}\delta_{\bar{D}j} I\left(W_{Di} = w_{Dk_D}\right) I\left(W_{\bar{D}j} = w_{\bar{D}k_{\bar{D}}}\right)} \frac{N_{Dk_D} N_{\bar{D}k_{\bar{D}}}}{N_D N_{\bar{D}}} = 1,$$

and thus

$$\widehat{AUC}_x(\hat{p}) = \frac{\widetilde{AUC}_x(\hat{p})}{\sum_{i=1}^{N_D}\sum_{j=1}^{N_{\bar{D}}} \frac{\delta_{Di}}{p_{Di}}\frac{\delta_{\bar{D}j}}{p_{\bar{D}j}}/(N_D N_{\bar{D}})} = \widetilde{AUC}_x(\hat{p}).$$

Appendix B: Proof of Theorem 1

B1. Asymptotic normality of $\widetilde{AUC}(p)$

In Appendix B, we use AUC to indicate AUC_x. The subscript x is omitted for simplicity.

First, with $AUC = E\left\{S_D(X_{\bar{D}})\right\}$, note that

$$\sqrt{N}\left\{\widetilde{AUC}(p) - AUC\right\}$$

$$= \sqrt{N}\left\{\frac{1}{N_D}\frac{1}{N_{\bar{D}}}\sum_{i=1}^{N_D}\sum_{j=1}^{N_{\bar{D}}}\frac{\delta_{Di}}{p_{Di}}\frac{\delta_{\bar{D}j}}{p_{\bar{D}j}} I(X_{Di} > X_{\bar{D}j}) - \sum_{j=1}^{N_{\bar{D}}}\frac{\delta_{\bar{D}j}}{p_{\bar{D}j}N_{\bar{D}}} S_D(X_{\bar{D}j})\right\}$$

$$+ \sqrt{N}\left\{\sum_{j=1}^{N_{\bar{D}}}\frac{\delta_{\bar{D}j}}{p_{\bar{D}j}N_{\bar{D}}} S_D(X_{\bar{D}j}) - E\{S_D(X_{\bar{D}})\}\right\}$$

$$= A + B + \sqrt{N}\left\{\frac{1}{N_D}\frac{1}{N_{\bar{D}}}\sum_{i=1}^{N_D}\sum_{j=1}^{N_{\bar{D}}}\frac{\delta_{Di}}{p_{Di}}\frac{\delta_{\bar{D}j}}{p_{\bar{D}j}} I(X_{Di} > X_{\bar{D}j}) - \sum_{j=1}^{N_{\bar{D}}}\frac{\delta_{\bar{D}j}}{p_{\bar{D}j}N_{\bar{D}}} S_D(X_{\bar{D}j})\right\}$$

$$- \sqrt{N}\left\{\sum_{i=1}^{N_D}\frac{\delta_{Di}}{p_{Di}N_D} F_{\bar{D}}(X_{Di}) - E\{S_D(X_{\bar{D}})\}\right\}$$

$$= A + B + \sqrt{N}\left[\sum_{j=1}^{N_{\bar D}} \frac{\delta_{\bar D j}}{p_{\bar D j} N_{\bar D}} \left\{\frac{1}{N_D}\sum_{i=1}^{N_D}\frac{\delta_{Di}}{p_{Di}} I(X_{Di} > X_{\bar D j}) - S_D(X_{\bar D j})\right\} - \left\{\frac{1}{N_D}\sum_{i=1}^{N_D}\frac{\delta_{Di}}{p_{Di}} F_{\bar D}(X_{Di}) - E\{S_D(X_{\bar D})\}\right\}\right]$$
$$= A + B + o_p(1),$$

where
$$A = \sqrt{N}\left\{\sum_{i=1}^{N_D}\frac{\delta_{Di}}{p_{Di}N_D} F_{\bar D}(X_{Di}) - E\{S_D(X_{\bar D})\}\right\},$$
$$B = \sqrt{N}\left\{\sum_{j=1}^{N_{\bar D}}\frac{\delta_{\bar D j}}{p_{\bar D j}N_{\bar D}} S_D(X_{\bar D j}) - E\{S_D(X_{\bar D})\}\right\}.$$

Asymptotic normality of $\sqrt{N}\{\widetilde{AUC}(p) - AUC\}$ follows trivially, given A and B are sums of independent observations of mean zero. That is, as $N \to \infty$, $\sqrt{N}\{\widetilde{AUC}(p) - AUC\}$ converges to a normal random variable with mean 0 and variance $\Sigma_x^{(I)} = \mathrm{var}(A) + \mathrm{var}(B)$.

Let σ_{N_D} indicate the sigma field of information, or complete data, potentially available in all cases in phase-one sample, and $\sigma_{N_{\bar D}}$ the sigma field of information for all controls in phase-one sample. We have

$$E(A) = E\left(E\left[\sqrt{N}\left\{\sum_{i=1}^{N_D}\frac{\delta_{Di}}{p_{Di}N_D} F_{\bar D}(X_{Di}) - E\{F_{\bar D}(X_D)\}\right\} | \sigma_{N_D}\right]\right)$$
$$= E\left[\sqrt{N}\left\{\frac{1}{N_D}\sum_{i=1}^{N_D} F_{\bar D}(X_{Di}) - E\{F_{\bar D}(X_D)\}\right\}\right] = 0.$$

$$E(B) = E\left(E\left[\sqrt{N}\left\{\sum_{j=1}^{N_{\bar D}}\frac{\delta_{\bar D j}}{p_{\bar D j}N_{\bar D}} S_D(X_{\bar D j}) - E\{S_D(X_{\bar D})\}\right\} | \sigma_{N_{\bar D}}\right]\right)$$
$$= E\left[E\left\{\sqrt{N}\left\{\frac{1}{N_{\bar D}}\sum_{j=1}^{N_{\bar D}} S_D(X_{\bar D j}) - E\{S_D(X_{\bar D})\}\right\}\right\}\right] = 0.$$

And
$$\mathrm{var}(A) = \frac{N_D}{N}\left(\mathrm{var}\left[E\left\{\frac{\delta_D}{p_D}F_{\bar D}(X_D) \mid \sigma_{N_D}\right\}\right] + E\left[\mathrm{var}\left\{\frac{\delta_D}{p_D}F_{\bar D}(X_D) \mid \sigma_{N_D}\right\}\right]\right)$$
$$= \frac{1}{\lambda} \times \left[\mathrm{var}\{F_{\bar D}(X_D)\} + E\left\{\frac{p_D(1-p_D)}{p_D^2}F_{\bar D}^2(X_D)\right\}\right],$$

$$\mathrm{var}(B) = \frac{N_{\bar D}}{N}\left(\mathrm{var}\left[E\left\{\frac{\delta_{\bar D}}{p_{\bar D}}S_D(X_{\bar D}) \mid \sigma_{N_{\bar D}}\right\}\right] + E\left[\mathrm{var}\left\{\frac{\delta_{\bar D}}{p_{\bar D}}S_D(X_{\bar D}) \mid \sigma_{N_{\bar D}}\right\}\right]\right)$$
$$= \frac{1}{1-\lambda} \times \left[\mathrm{var}\{S_D(X_{\bar D})\} + E\left\{\frac{p_{\bar D}(1-p_{\bar D})}{p_{\bar D}^2}S_D^2(X_{\bar D})\right\}\right].$$

B2. Asymptotic normality of $\widehat{AUC}(p)$

Next we consider the asymptotic distribution of

$$\widehat{AUC}(p) = \frac{\widetilde{AUC}(p)}{\sum_{i=1}^{N_D}\sum_{j=1}^{N_{\bar{D}}} \frac{\delta_{Di}}{p_{Di}}\frac{\delta_{\bar{D}j}}{p_{\bar{D}j}}/(N_D N_{\bar{D}})}.$$

Note that

$$\begin{aligned}
&\sqrt{N}\left\{\begin{pmatrix} \widetilde{AUC}(p) \\ \frac{1}{N_D N_{\bar{D}}}\sum_{i=1}^{N_D}\sum_{j=1}^{N_{\bar{D}}} \frac{\delta_{Di}}{p_{Di}}\frac{\delta_{\bar{D}j}}{p_{\bar{D}j}} \end{pmatrix} - \begin{pmatrix} AUC \\ 1 \end{pmatrix}\right\} \\
&= \left\{\begin{array}{c} \sqrt{N}\left\{\sum_{i=1}^{N_D} \frac{\delta_{Di}}{p_{Di}N_D} F_{\bar{D}}(X_{Di}) - E\{S_D(X_{\bar{D}})\}\right\} + \sqrt{N}\left\{\sum_{j=1}^{N_{\bar{D}}} \frac{\delta_{\bar{D}j}}{p_{\bar{D}j}N_{\bar{D}}} S_D(X_{\bar{D}j}) - E\{S_D(X_{\bar{D}})\}\right\} + o_p(1) \\ \sqrt{N}\left(\frac{1}{N_D}\frac{1}{N_{\bar{D}}}\sum_{i=1}^{N_D}\sum_{j=1}^{N_{\bar{D}}} \frac{\delta_{Di}}{p_{Di}}\frac{\delta_{\bar{D}j}}{p_{\bar{D}j}} - \frac{1}{N_{\bar{D}}}\sum_{j=1}^{N_{\bar{D}}} \frac{\delta_{\bar{D}j}}{p_{\bar{D}j}}\right) + \sqrt{N}\left(\frac{1}{N_{\bar{D}}}\sum_{j=1}^{N_{\bar{D}}} \frac{\delta_{\bar{D}j}}{p_{\bar{D}j}} - 1\right) \end{array}\right\} \\
&= \left\{\begin{array}{c} \sqrt{N}\left\{\sum_{i=1}^{N_D} \frac{\delta_{Di}}{p_{Di}N_D} F_{\bar{D}}(X_{Di}) - E\{S_D(X_{\bar{D}})\}\right\} + \sqrt{N}\left\{\sum_{j=1}^{N_{\bar{D}}} \frac{\delta_{\bar{D}j}}{p_{\bar{D}j}N_{\bar{D}}} S_D(X_{\bar{D}j}) - E\{S_D(X_{\bar{D}})\}\right\} + o_p(1) \\ \sqrt{N}\left(\frac{1}{N_D}\sum_{i=1}^{N_D} \frac{\delta_{Di}}{p_{Di}} - 1\right) + \sqrt{N}\left(\frac{1}{N_{\bar{D}}}\sum_{j=1}^{N_{\bar{D}}} \frac{\delta_{\bar{D}j}}{p_{\bar{D}j}} - 1\right) + o_p(1) \end{array}\right\} \\
&= \left\{\begin{array}{c} A + B + o_p(1) \\ E + F + o_p(1) \end{array}\right\}
\end{aligned}$$

with

$$E = \sqrt{N}\left(\frac{1}{N_D}\sum_{i=1}^{N_D} \frac{\delta_{Di}}{p_{Di}} - 1\right), \qquad F = \sqrt{N}\left(\frac{1}{N_{\bar{D}}}\sum_{j=1}^{N_{\bar{D}}} \frac{\delta_{\bar{D}j}}{p_{\bar{D}j}} - 1\right).$$

Note that $\left\{\begin{array}{c} A + B \\ E + F \end{array}\right\}$ can be written as the sum of two independent terms

$$\frac{\sqrt{N}}{\sqrt{N_D}}\sqrt{N_D}\left\{\frac{1}{N_D}\sum_{i=1}^{N_D}\begin{pmatrix} \frac{\delta_{Di}}{p_{Di}} F_{\bar{D}}(X_{Di}) \\ \frac{\delta_{Di}}{p_{Di}} \end{pmatrix} - \begin{pmatrix} E\{F_{\bar{D}}(X_D)\} \\ 1 \end{pmatrix}\right\}$$

and

$$\frac{\sqrt{N}}{\sqrt{N_{\bar{D}}}}\sqrt{N_{\bar{D}}}\left\{\frac{1}{N_{\bar{D}}}\sum_{i=1}^{N_{\bar{D}}}\begin{pmatrix} \frac{\delta_{\bar{D}i}}{p_{\bar{D}j}} S_D(X_{\bar{D}j}) \\ \frac{\delta_{\bar{D}j}}{p_{\bar{D}j}} \end{pmatrix} - \begin{pmatrix} E\{S_D(X_{\bar{D}})\} \\ 1 \end{pmatrix}\right\},$$

where each term follows bivariate normal distribution following multivariate central limit theorem.

We thus have

$$\sqrt{N}\left\{\begin{pmatrix} \widetilde{AUC}(p) \\ \frac{1}{N_D N_{\bar{D}}}\sum_{i=1}^{N_D}\sum_{j=1}^{N_{\bar{D}}} \frac{\delta_{Di}}{p_{Di}}\frac{\delta_{\bar{D}j}}{p_{\bar{D}j}} \end{pmatrix} - \begin{pmatrix} AUC \\ 1 \end{pmatrix}\right\}$$

asymptotically bivariate normal.

The asymptotic normality of $\sqrt{N}\left\{\widehat{AUC}(p) - AUC\right\}$ follows according to Delta method. That is, as $N \to \infty$, $\sqrt{N}\{\widehat{AUC}(p) - AUC\}$ converges to a normal random variable with mean 0 and variance $\Sigma_x^{(II)}$, which is computed as below. We have

$$E(E) = E\left[E\left\{\sqrt{N}\left(\frac{1}{N_D}\sum_{i=1}^{N_D}\frac{\delta_{Di}}{p_{Di}} - 1\right)|\sigma_{N_D}\right\}\right] = E\left[\sqrt{N}\,(1-1)\right] = 0,$$

$$E(F) = E\left[E\left\{\sqrt{N}\left(\frac{1}{N_{\bar{D}}}\sum_{j=1}^{N_{\bar{D}}}\frac{\delta_{\bar{D}j}}{p_{\bar{D}j}} - 1\right)|\sigma_{N_{\bar{D}}}\right\}\right] = E\left[\sqrt{N}\,(1-1)\right] = 0,$$

$$\begin{aligned}\operatorname{var}(E) &= \operatorname{var}\left[E\left\{\sqrt{N}\left(\frac{1}{N_D}\sum_{i=1}^{N_D}\frac{\delta_{Di}}{p_{Di}} - 1\right)|\,\sigma_{N_D}\right\}\right] + E\left[\operatorname{var}\left\{\sqrt{N}\left(\frac{1}{N_D}\sum_{i=1}^{N_D}\frac{\delta_{Di}}{p_{Di}} - 1\right)|\,\sigma_{N_D}\right\}\right]\\ &= 0 + \frac{n}{N_D}E\left\{\operatorname{var}\left(\frac{\delta_D}{p_D}\,|\,\sigma_{N_D}\right)\right\}\\ &= \frac{1}{\lambda}\times E\left(\frac{1}{p_D} - 1\right).\end{aligned}$$

Similarly, we can show that $\operatorname{var}(F) = \frac{1}{1-\lambda}\times E\left(\frac{1}{p_{\bar{D}}} - 1\right).$

Moreover,

$$\begin{aligned}\operatorname{cov}(B,F) &= \operatorname{cov}\left\{\sqrt{N}\left\{\sum_{j=1}^{N_{\bar{D}}}\frac{\delta_{\bar{D}j}}{p_{\bar{D}j}}S_D(X_{\bar{D}j}) - E\{S_D(X_{\bar{D}})\}\right\}, \sqrt{n}\left(\frac{1}{N_{\bar{D}}}\sum_{j=1}^{N_{\bar{D}}}\frac{\delta_{\bar{D}j}}{p_{\bar{D}j}} - 1\right)\right\}\\ &= \frac{N}{N_{\bar{D}}}\operatorname{cov}\left\{\frac{\delta_{\bar{D}}}{p_{\bar{D}}}S_D(X_{\bar{D}}), \frac{\delta_{\bar{D}}}{p_{\bar{D}}}\right\}\\ &= \frac{1}{1-\lambda}\left(\left[\operatorname{cov}\left\{E\left(\frac{\delta_{\bar{D}}}{p_{\bar{D}}}S_D(X_{\bar{D}})|\sigma_{N_{\bar{D}}}\right), E\left(\frac{\delta_{\bar{D}}}{p_{\bar{D}}}|\sigma_{N_{\bar{D}}}\right)\right\}\right] + E\left[\operatorname{cov}\left\{\frac{\delta_{\bar{D}}}{p_{\bar{D}}}S_D(X_{\bar{D}}), \frac{\delta_{\bar{D}}}{p_{\bar{D}}}|\sigma_{N_{\bar{D}}}\right\}\right]\right)\\ &= \frac{1}{1-\lambda}\times E\left\{\left(\frac{1}{p_{\bar{D}}} - 1\right)S_D(X_{\bar{D}})\right\}.\end{aligned}$$

Similarly, we have

$$\operatorname{cov}(A,E) = \frac{1}{\lambda}E\left\{\left(\frac{1}{p_D} - 1\right)F_{\bar{D}}(X_D)\right\}.$$

Thus by Delta method,

$$\begin{aligned}\Sigma_x^{(II)} &= \operatorname{var}\left\{\sqrt{N}\left(\widehat{AUC}(p) - AUC\right)\right\} + o_p(1)\\ &= \begin{pmatrix} 1 & -AUC \end{pmatrix}\begin{pmatrix} \operatorname{var}(A) + \operatorname{var}(B) & \operatorname{cov}(A,E) + \operatorname{cov}(B,F) \\ \operatorname{cov}(A,E) + \operatorname{cov}(B,F) & \operatorname{var}(E) + \operatorname{var}(F) \end{pmatrix}\begin{pmatrix} 1 \\ -AUC \end{pmatrix}\\ &= \operatorname{var}(A) + \operatorname{var}(B) - 2AUC\left\{\operatorname{cov}(A,E) + \operatorname{cov}(B,F)\right\} + AUC^2\left\{\operatorname{var}(E) + \operatorname{var}(F)\right\}\end{aligned}$$

$$
\begin{aligned}
&= \mathrm{var}(A) + \mathrm{var}(B) - AUC \times \left(\frac{1}{\lambda} \times \left[2E\left\{ \left(\frac{1}{p_D} - 1 \right) F_{\bar{D}}(X_D) \right\} - AUC \times E\left(\frac{1}{p_D} - 1 \right) \right] \right. \\
&\left. + \frac{1}{1-\lambda} \times \left[2E\left\{ \left(\frac{1}{p_{\bar{D}}} - 1 \right) S_D(X_{\bar{D}}) \right\} - AUC \times E\left(\frac{1}{p_{\bar{D}}} - 1 \right) \right] \right) \\
&= \Sigma_x^{(I)} - AUC \times \left(\frac{1}{\lambda} \times \left[E\left\{ \left(\frac{1}{p_D} - 1 \right) F_{\bar{D}}(X_D) \right\} + \mathrm{cov}\left(F_{\bar{D}}(X_D), \frac{1}{p_D} - 1 \right) \right] \right. \\
&\left. + \frac{1}{1-\lambda} \times \left[E\left\{ \left(\frac{1}{p_{\bar{D}}} - 1 \right) S_D(X_{\bar{D}}) \right\} + \mathrm{cov}\left(S_D(X_{\bar{D}}), \frac{1}{p_{\bar{D}}} - 1 \right) \right] \right).
\end{aligned}
$$

The comparison of efficiency between $\widetilde{AUC}_x(p)$ and $\widehat{AUC}_x(p)$ depends on the sampling design and the biomarker distribution. Among other conditions, one condition that would ensure $\Sigma_x^{(II)} \leqslant \Sigma_x^{(I)}$ is the non-negativity of the covariances between $1/p_D$ and $F_{\bar{D}x}(X_D)$ and between $1/p_{\bar{D}}$ and $S_{Dx}(X_{\bar{D}})$. A special case when this holds is when cases and controls each are randomly sampled in the second phase such that the phase-two sampling probability of a subject is independent of one's biomarker value.

B3. Asymptotic normality of $\widetilde{AUC}(\hat{p})$

Suppose we model $\hat{p}_D$ as a function of covariates with finite-dimensional parameters $\theta_D = \{\theta_{D1}, \ldots, \theta_{DK_D}\}$, and model $p_{\bar{D}}$ as a function of covariates with finite-dimensional parameters $\theta_{\bar{D}} = \{\theta_{\bar{D}1}, \ldots, \theta_{\bar{D}K_D}\}$. Let $\hat{\theta}_D$ and $\hat{\theta}_{\bar{D}}$ be maximum likelihood estimators of θ_D and $\theta_{\bar{D}}$, and let $\hat{p}_D$ and $\hat{p}_{\bar{D}}$ be corresponding estimators of p_D and $p_{\bar{D}}$. Through Taylor's expansion, we have

$$
\begin{aligned}
\sqrt{N}\left\{ \widetilde{AUC}(\hat{p}) - AUC \right\} &= \sqrt{n}\left\{ \widetilde{AUC}(\hat{p}) - \widetilde{AUC}(p) \right\} + \sqrt{n}\left\{ \widetilde{AUC}(p) - AUC \right\} \\
&= \frac{\partial E\{\widetilde{AUC}(p)\}}{\partial \theta_D} \sqrt{n}(\hat{\theta}_D - \theta_D) + \frac{\partial E\{\widetilde{AUC}(p)\}}{\partial \theta_{\bar{D}}} \sqrt{n}(\hat{\theta}_{\bar{D}} - \theta_{\bar{D}}) + A + B + o_p(1) \\
&= C + D + A + B + o_p(1),
\end{aligned}
$$

where

$$
\begin{aligned}
C &= \frac{\partial E\{\widetilde{AUC}(p)\}}{\partial \theta_D} \sqrt{n}(\hat{\theta}_D - \theta_D) \\
&= -\frac{1}{N_D \times N_{\bar{D}}} E\left\{ \sum_{i=1}^{N_D} \sum_{j=1}^{N_{\bar{D}}} \frac{\delta_{Di}}{p_{Di}^2} \frac{\delta_{\bar{D}j}}{p_{\bar{D}j}} I(X_{Di} > X_{\bar{D}j}) \frac{\partial p_D}{\partial \theta_D} \right\} \sqrt{n}(\hat{\theta}_D - \theta_D) \\
&= -E\left\{ I(X_D > X_{\bar{D}}) \frac{1}{p_D} \frac{\partial p_D}{\partial \theta_D} \right\} \sqrt{N}\left(\hat{\theta}_D - \theta_D \right)
\end{aligned}
$$

$$
\begin{aligned}
D &= \frac{\partial E\left\{\widetilde{AUC}(p)\right\}}{\partial \theta_{\bar{D}}} \sqrt{n}(\hat{\theta}_{\bar{D}} - \theta_{\bar{D}}) \\
&= -\frac{1}{N_D \times N_{\bar{D}}} E\left\{\sum_{i=1}^{n_D}\sum_{j=1}^{n_{\bar{D}}} \frac{\delta_{Di}}{p_{Di}} \frac{\delta_{\bar{D}j}}{p_{\bar{D}j}^2} I(X_{Di} > X_{\bar{D}j}) \frac{\partial p_{\bar{D}}}{\partial \theta_{\bar{D}}}\right\} \sqrt{n}(\hat{\theta}_{\bar{D}} - \theta_{\bar{D}}) \\
&= -E\left\{I(X_D > X_{\bar{D}}) \frac{1}{p_{\bar{D}}} \frac{\partial p_{\bar{D}}}{\partial \theta_{\bar{D}}}\right\} \sqrt{n}(\hat{\theta}_{\bar{D}} - \theta_{\bar{D}}).
\end{aligned}
$$

Therefore, as $N \to \infty$, $\sqrt{N}\{\widetilde{AUC}(\hat{p}) - AUC\}$ converges to a normal random variable with mean 0 and variance $\Sigma_x^{(III)}$. We have

$$
\Sigma_x^{(III)} = \mathrm{var}(C) + \mathrm{var}(D) + \mathrm{var}(A) + \mathrm{var}(B) + 2\mathrm{cov}(A, C) + 2\mathrm{cov}(B, D).
$$

Considering estimating $\hat{\theta}_{\bar{D}}$ by maximizing the likelihood of observing markers in phase two. Log-likelihood for sampling a control from the phase-one sample is

$$
l_{\bar{D}} = \delta_{\bar{D}} \log(p_{\bar{D}}) + (1 - \delta_{\bar{D}}) \log(1 - p_{\bar{D}}).
$$

The corresponding score function is

$$
\frac{\partial l_{\bar{D}}}{\partial \theta_{\bar{D}k_{\bar{D}}}} = \left(\frac{\delta_{\bar{D}}}{p_{\bar{D}}} - \frac{1 - \delta_{\bar{D}}}{1 - p_{\bar{D}}}\right) \frac{\partial p_{\bar{D}}}{\partial \theta_{\bar{D}k_{\bar{D}}}},
$$

the Hessian is

$$
\frac{\partial l_{\bar{D}}^2}{\partial \theta_{\bar{D}k_{\bar{D}}} \partial \theta_{\bar{D}k'_{\bar{D}}}} = \left(\frac{\delta_{\bar{D}}}{p_{\bar{D}}} - \frac{1 - \delta_{\bar{D}}}{1 - p_{\bar{D}}}\right) \frac{p_{\bar{D}}^2}{\partial \theta_{\bar{D}k_{\bar{D}}} \partial \theta_{\bar{D}k'_{\bar{D}}}} - \left(\frac{\delta_{\bar{D}}}{p_{\bar{D}}^2} + \frac{1 - \delta_{\bar{D}}}{(1 - p_{\bar{D}})^2}\right) \frac{\partial p_{\bar{D}k}}{\partial \theta_{\bar{D}k_{\bar{D}}}} \frac{\partial p_{\bar{D}k'_{\bar{D}}}}{\partial \theta_{\bar{D}k'_{\bar{D}}}},
$$

and the information matrix is

$$
I_{\bar{D}} = -E\left(\frac{\partial l^2}{\partial \theta_{\bar{D}k_{\bar{D}}} \partial \theta_{\bar{D}k'_{\bar{D}}}}\right) = E\left\{\left(\frac{1}{p_{\bar{D}}} + \frac{1}{1 - p_{\bar{D}}}\right) \frac{\partial p_{\bar{D}k}}{\partial \theta_{\bar{D}k_{\bar{D}}}} \frac{\partial p_{\bar{D}k'_{\bar{D}}}}{\partial \theta_{\bar{D}k'_{\bar{D}}}}\right\}.
$$

Note

$$
\begin{aligned}
\mathrm{var}(D) &= \frac{N}{N_{\bar{D}}} \left[-E\left\{I(X_D > X_{\bar{D}}) \frac{1}{p_{\bar{D}}} \frac{\partial p_{\bar{D}}}{\partial \theta_{\bar{D}}}\right\}\right]^T I_{\bar{D}}^{-1} \left[-E\left\{I(X_D > X_{\bar{D}}) \frac{1}{p_{\bar{D}}} \frac{\partial p_{\bar{D}}}{\partial \theta_{\bar{D}}}\right\}\right] \\
&= \frac{1}{1 - \lambda} \left[E\left\{I(X_D > X_{\bar{D}}) \frac{1}{p_{\bar{D}}} \frac{\partial p_{\bar{D}}}{\partial \theta_{\bar{D}}}\right\}\right]^T I_{\bar{D}}^{-1} \left[E\left\{I(X_D > X_{\bar{D}}) \frac{1}{p_{\bar{D}}} \frac{\partial p_{\bar{D}}}{\partial \theta_{\bar{D}}}\right\}\right],
\end{aligned}
$$

$$\begin{aligned}
\operatorname{cov}(B, D) &= \frac{1}{1-\lambda}\operatorname{cov}\left(\frac{\delta_{\bar{D}}}{p_{\bar{D}}}S_D(X_{\bar{D}}), \left[-E\left\{I(X_D > X_{\bar{D}})\frac{1}{p_D}\frac{\partial p_{\bar{D}}}{\partial\theta_{\bar{D}}}\right\}\right]^T I_{\bar{D}}^{-1}\frac{\partial l_{\bar{D}}}{\partial\theta_{\bar{D}}}\right)\\
&= \frac{1}{1-\lambda}\operatorname{cov}\left(\frac{\delta_{\bar{D}}}{p_{\bar{D}}}S_D(X_{\bar{D}}), \left[-E\left\{I(X_D > X_{\bar{D}})\frac{1}{p_D}\frac{\partial p_{\bar{D}}}{\partial\theta_{\bar{D}}}\right\}\right]^T I_{\bar{D}}^{-1}\left(\frac{\delta_{\bar{D}}}{p_{\bar{D}}} - \frac{1-\delta_{\bar{D}}}{1-p_{\bar{D}}}\right)\frac{\partial p_{\bar{D}}}{\partial\theta_{\bar{D}}}\right)\\
&= -\frac{1}{1-\lambda}\left[E\left\{I(X_D > X_{\bar{D}})\frac{\partial p_{\bar{D}}}{\partial\theta_{\bar{D}}}\right\}\right]^T I_{\bar{D}}^{-1} E\left[S_D(X_{\bar{D}})\frac{1}{p_{\bar{D}}}\frac{\partial p_{\bar{D}}}{\partial\theta_{\bar{D}}}\right]\\
&= -\operatorname{var}(D).
\end{aligned}$$

Similarly we can show that

$$\operatorname{cov}(A, C) = -\operatorname{var}(C) = -\frac{1}{\lambda}\left[E\left\{I(X_D > X_{\bar{D}})\frac{1}{p_D}\frac{\partial p_D}{\partial\theta_D}\right\}\right]^T I_D^{-1} E\left[I(X_D > X_{\bar{D}})\frac{1}{p_D}\frac{\partial p_D}{\partial\theta_D}\right]$$

Thus $\Sigma_x^{(III)} = \operatorname{var}(A) + \operatorname{var}(B) - \operatorname{var}(C) - \operatorname{var}(D) \leqslant \operatorname{var}(A) + \operatorname{var}(B) = \Sigma_x^{(I)}$.

B4. Asymptotic normality of $\widehat{AUC}(\hat{p})$

Next we derive asymptotic distribution of $\sqrt{N}\{\widehat{AUC}(\hat{p}) - AUC\}$. Following similar arguments as in B2 and B3,

$$\begin{aligned}
&\sqrt{N}\left\{\begin{pmatrix} \widetilde{AUC}(\hat{p}) \\ \frac{1}{N_D N_{\bar{D}}}\sum_{i=1}^{N_D}\sum_{j=1}^{N_{\bar{D}}}\frac{\delta_{Di}}{\hat{p}_{Di}}\frac{\delta_{\bar{D}j}}{\hat{p}_{\bar{D}j}} \end{pmatrix} - \begin{pmatrix} AUC \\ 1 \end{pmatrix}\right\}\\
&= \left\{\begin{pmatrix} C + D + A + B + o_p(1) \\ \sqrt{N}\left(\frac{1}{N_D}\frac{1}{N_{\bar{D}}}\sum_{i=1}^{N_D}\sum_{j=1}^{N_{\bar{D}}}\frac{\delta_{Di}}{\hat{p}_{Di}}\frac{\delta_{\bar{D}j}}{\hat{p}_{\bar{D}j}} - \frac{1}{N_D}\frac{1}{N_{\bar{D}}}\sum_{i=1}^{N_D}\sum_{j=1}^{N_{\bar{D}}}\frac{\delta_{Di}}{p_{Di}}\frac{\delta_{Dj}}{p_{\bar{D}j}}\right) + \sqrt{N}\left(\frac{1}{N_D}\frac{1}{N_{\bar{D}}}\sum_{i=1}^{N_D}\sum_{j=1}^{N_{\bar{D}}}\frac{\delta_{Di}}{p_{Di}}\frac{\delta_{\bar{D}j}}{p_{\bar{D}j}} - 1\right) \end{pmatrix}\right\}\\
&= \left\{\begin{pmatrix} C + D + A + B + o_p(1) \\ -\frac{1}{N_D N_{\bar{D}}}E\left\{\sum_{i=1}^{D}\sum_{j=1}^{N_{\bar{D}}}\frac{\delta_{Di}}{p_{Di}^2}\frac{\delta_{\bar{D}j}}{p_{\bar{D}j}}\frac{\partial p_D}{\partial\theta_D}\right\}\sqrt{N}\left(\hat{\theta}_D - \theta_D\right) - \frac{1}{N_D N_{\bar{D}}}E\left\{\sum_{i=1}^{D}\sum_{j=1}^{N_{\bar{D}}}\frac{\delta_{Di}}{p_{Di}}\frac{\delta_{\bar{D}j}}{p_{\bar{D}j}^2}\frac{\partial p_{\bar{D}}}{\partial\theta_{\bar{D}}}\right\}\sqrt{n}\left(\hat{\theta}_{\bar{D}} - \theta_{\bar{D}}\right) + \\ E + F + o_p(1) \end{pmatrix}\right\}\\
&= \left\{\begin{pmatrix} C + D + A + B + o_p(1) \\ G + H + E + F + o_p(1) \end{pmatrix}\right\}
\end{aligned}$$

is asymptotically bivariate normal, where

$$\begin{aligned}
G &= -E\left(\frac{1}{p_D}\frac{\partial p_D}{\partial\theta_D}\right)\sqrt{N}\left(\hat{\theta}_D - \theta_D\right),\\
H &= -E\left(\frac{1}{p_{\bar{D}}}\frac{\partial p_{\bar{D}}}{\partial\theta_{\bar{D}}}\right)\sqrt{N}\left(\hat{\theta}_{\bar{D}} - \theta_{\bar{D}}\right).
\end{aligned}$$

And the asymptotic normality of $\sqrt{N}\left\{\widehat{AUC}(\hat{p}) - AUC\right\}$ follows. Denote its asymptotic variance as $\Sigma_x^{(IV)}$. Denote

$$M = \sqrt{N}\left(\frac{1}{N_D}\frac{1}{N_{\bar{D}}}\sum_{i=1}^{N_D}\sum_{j=1}^{N_{\bar{D}}}\frac{\delta_{Di}}{\hat{p}_{Di}}\frac{\delta_{\bar{D}j}}{\hat{p}_{\bar{D}j}} - 1\right).$$

By Delta method,

$$\Sigma_x^{(IV)} = \text{var}\left\{\sqrt{N}\left(\widehat{AUC}(\hat{p}) - AUC\right)\right\} + o_p(1)$$

$$= \begin{pmatrix} 1 & -AUC \end{pmatrix} \begin{pmatrix} \text{var}(\sqrt{N}\left(\widetilde{AUC}(\hat{p}) - AUC\right), & \text{cov}\left[\sqrt{N}\left\{\widetilde{AUC}(\hat{p}) - AUC\right\}, M\right] \\ \text{cov}\left[\sqrt{N}\left\{\widetilde{AUC}(\hat{p}) - AUC\right\}, M\right] & \text{var}(M)) \end{pmatrix} \begin{pmatrix} 1 \\ -AUC \end{pmatrix} + o_p(1)$$

$$= \text{var}(A) + \text{var}(B) - \text{var}(C) - \text{var}(D) - 2AUC \times \text{cov}(A + B + C + D, E + F + G + H)$$

$$+ AUC^2 \text{var}(E + F + G + H)$$

$$= \text{var}(A) + \text{var}(B) + \text{var}(C) + \text{var}(D) + 2\text{cov}(A, C) + 2\text{cov}(B, D)$$

$$- 2AUC \{\text{cov}(A, E) + \text{cov}(C, E) + \text{cov}(B, F) + \text{cov}(D, F)$$

$$+ \text{cov}(A, G) + \text{cov}(C, G) + \text{cov}(B, H) + \text{cov}(D, H)\}$$

$$+ AUC^2 \{\text{var}(E) + \text{var}(F) + \text{var}(G) + \text{var}(H) + 2\text{cov}(E, G) + 2\text{cov}(F, H)\}$$

$$= \Sigma_x^{(II)} - \text{var}(C) - \text{var}(D)$$

$$- 2AUC \{\text{cov}(C, E) + \text{cov}(D, F) + \text{cov}(A, G) + \text{cov}(C, G) + \text{cov}(B, H) + \text{cov}(D, H)\}$$

$$+ AUC^2 \{\text{var}(G) + \text{var}(H) + 2\text{cov}(E, G) + 2\text{cov}(F, H)\}.$$

Note

$$\text{cov}(D, F)$$

$$= \text{cov}\left[-\sqrt{N}E\left\{I(X_D > X_{\bar{D}})\frac{\partial p_{\bar{D}}}{\partial \theta_{\bar{D}}}\right\}\frac{1}{N_{\bar{D}}}\sum_{j=1}^{N_{\bar{D}}} I_{\bar{D}}^{-1} \times \left(\frac{\delta_{\bar{D}}}{p_{\bar{D}}} - \frac{1-\delta_{\bar{D}}}{1-p_{\bar{D}}}\right)\frac{\partial p_{\bar{D}}}{\partial \theta_{\bar{D}}}, \sqrt{N}\left(\frac{1}{N_{\bar{D}}}\sum_{j=1}^{N_{\bar{D}}}\frac{\delta_{\bar{D}j}}{p_{\bar{D}j}} - 1\right)\right]$$

$$= \frac{1}{1-\lambda} \times \text{cov}\left[-E\left\{I(X_D > X_{\bar{D}})\frac{\partial p_{\bar{D}}}{\partial \theta_{\bar{D}}}\right\} I_{\bar{D}}^{-1} \times \left(\frac{\delta_{\bar{D}}}{p_{\bar{D}}} - \frac{1-\delta_{\bar{D}}}{1-p_{\bar{D}}}\right)\frac{\partial p_{\bar{D}}}{\partial \theta_{\bar{D}}}, \frac{\delta_{\bar{D}j}}{p_{\bar{D}j}} - 1\right]$$

$$= -\frac{1}{1-\lambda} \times E\left\{I(X_D > X_{\bar{D}})\frac{\partial p_{\bar{D}}}{\partial \theta_{\bar{D}}}\right\} I_{\bar{D}}^{-1} \times E\left(\frac{\delta_{\bar{D}}^2}{p_{\bar{D}}^2}\frac{\partial p_{\bar{D}}}{\partial \theta_{\bar{D}}}\right)$$

$$= -\frac{1}{1-\lambda} \times E\left\{I(X_D > X_{\bar{D}})\frac{\partial p_{\bar{D}}}{\partial \theta_{\bar{D}}}\right\} I_{\bar{D}}^{-1} E\left(\frac{1}{p_{\bar{D}}}\frac{\partial p_{\bar{D}}}{\partial \theta_{\bar{D}}}\right)$$

Similarly,

$$\text{cov}(C, E) = -\frac{1}{\lambda} \times E\left\{I(X_D > X_{\bar{D}})\frac{\partial p_D}{\partial \theta_D}\right\} I_D^{-1} E\left(\frac{1}{p_D}\frac{\partial p_D}{\partial \theta_D}\right).$$

In addition,

$$\text{var}(G) = \frac{N}{N_D}(-1) \times E\left(\frac{1}{p_D}\frac{\partial p_D}{\partial \theta_D}\right)^T I_D^{-1}(-1) \times E\left(\frac{1}{p_D}\frac{\partial p_D}{\partial \theta_D}\right)$$

$$= \frac{1}{\lambda}E\left(\frac{1}{p_D}\frac{\partial p_D}{\partial \theta_D}\right)^T I_D^{-1} E\left(\frac{1}{p_D}\frac{\partial p_D}{\partial \theta_D}\right),$$

$$
\begin{aligned}
\mathrm{var}(H) &= \frac{N}{N_{\bar{D}}}(-1) \times E\left(\frac{1}{p_{\bar{D}}}\frac{\partial p_{\bar{D}}}{\partial \theta_{\bar{D}}}\right)^T I_{\bar{D}}^{-1}(-1) \times E\left(\frac{1}{p_{\bar{D}}}\frac{\partial p_{\bar{D}}}{\partial \theta_{\bar{D}}}\right) \\
&= \frac{1}{\lambda} E\left(\frac{1}{p_{\bar{D}}}\frac{\partial p_{\bar{D}}}{\partial \theta_{\bar{D}}}\right)^T I_{\bar{D}}^{-1} E\left(\frac{1}{p_{\bar{D}}}\frac{\partial p_{\bar{D}}}{\partial \theta_{D}}\right), \\
\mathrm{cov}(E,G) &= \frac{1}{\lambda}\mathrm{cov}\left\{\frac{\delta_D}{p_D} - 1, -E\left(\frac{1}{p_D}\frac{\partial p_D}{\partial \theta_D}\right) I_D^{-1}\left(\frac{\delta_D}{p_D} - \frac{1-\delta_D}{1-p_D}\right)\frac{\partial p_D}{\partial \theta_D}\right\} \\
&= -\frac{1}{\lambda} E\left(\frac{1}{p_D}\frac{\partial p_D}{\partial \theta_D}\right) I_D^{-1} E\left(\frac{1}{p_D}\frac{\partial p_D}{\partial \theta_D}\right), \\
\mathrm{cov}(F,H) &= -\frac{1}{1-\lambda} E\left(\frac{1}{p_{\bar{D}}}\frac{\partial p_{\bar{D}}}{\partial \theta_{\bar{D}}}\right) I_{\bar{D}}^{-1} E\left(\frac{1}{p_{\bar{D}}}\frac{\partial p_{\bar{D}}}{\partial \theta_{\bar{D}}}\right), \\
\mathrm{cov}(A,G) &= -\frac{1}{\lambda} E\left(\frac{1}{p_D}\frac{\partial p_D}{\partial \theta_D}\right) I_D^{-1} E\left(\frac{1}{p_D} F_{\bar{D}}(X_D)\frac{\partial p_D}{\partial \theta_D}\right), \\
\mathrm{cov}(B,H) &= -\frac{1}{1-\lambda} E\left(\frac{1}{p_{\bar{D}}}\frac{\partial p_{\bar{D}}}{\partial \theta_{\bar{D}}}\right) I_{\bar{D}}^{-1} E\left(\frac{1}{p_{\bar{D}}} S_D(X_{\bar{D}})\frac{\partial p_{\bar{D}}}{\partial \theta_{\bar{D}}}\right), \\
\mathrm{cov}(C,G) &= \frac{1}{\lambda} E\left\{I(X_D > X_{\bar{D}})\frac{1}{p_D}\frac{\partial p_D}{\partial \theta_D}\right\} I_D^{-1} E\left\{\frac{1}{p_D}\frac{\partial p_D}{\partial \theta_D}\right\} \\
\mathrm{cov}(D,H) &= \frac{1}{1-\lambda} E\left\{I(X_D > X_{\bar{D}})\frac{1}{p_{\bar{D}}}\frac{\partial p_{\bar{D}}}{\partial \theta_{\bar{D}}}\right\} I_{\bar{D}}^{-1} E\left\{\frac{1}{p_{\bar{D}}}\frac{\partial p_{\bar{D}}}{\partial \theta_{\bar{D}}}\right\}.
\end{aligned}
$$

Consequently,

$$
\begin{aligned}
&-\mathrm{var}(C) - 2AUC\left\{\mathrm{cov}(C,E) + \mathrm{cov}(A,G) + \mathrm{cov}(C,G)\right\} + AUC^2\left\{\mathrm{var}(G) + 2\mathrm{cov}(E,G)\right\} \\
&= -\frac{1}{1-\lambda}\left[E\left\{I(X_D > X_{\bar{D}}\frac{1}{p_D}\frac{\partial p_D}{\partial \theta}\right\}^T I_D^{-1} E\left\{I(X_D > X_{\bar{D}}\frac{1}{p_D}\frac{\partial p_D}{\partial \theta}\right\}\right. \\
&- 2AUC \times \left[-E\left\{I(X_D > X_{\bar{D}}\frac{1}{p_D}\frac{\partial p_D}{\partial \theta}\right\}^T I_D^{-1} E\left\{\frac{1}{p_D}\frac{\partial p_D}{\partial \theta}\right\}\right. \\
&- E\left\{\frac{1}{p_D}\frac{\partial p_D}{\partial \theta}\right\}^T I_D^{-1} E\left\{I(X_D > X_{\bar{D}}\frac{1}{p_D}\frac{\partial p_D}{\partial \theta}\right\} + \left. E\left\{I(X_D > X_{\bar{D}}\frac{1}{p_D}\frac{\partial p_D}{\partial \theta}\right\}^T I_D^{-1} E\left\{\frac{1}{p_D}\frac{\partial p_D}{\partial \theta}\right\}\right] \\
&+ \left. AUC^2\left\{E\left\{\frac{1}{p_D}\frac{\partial p_D}{\partial \theta}\right\}^T I_D^{-1} E\left\{\frac{1}{p_D}\frac{\partial p_D}{\partial \theta}\right\} - 2E\left\{\frac{1}{p_D}\frac{\partial p_D}{\partial \theta}\right\}^T I_D^{-1} E\left\{\frac{1}{p_D}\frac{\partial p_D}{\partial \theta}\right\}\right\}\right] \\
&= -\frac{1}{1-\lambda}\left[E\left\{I(X_D > X_{\bar{D}}\frac{1}{p_D}\frac{\partial p_D}{\partial \theta}\right\}^T I_D^{-1} E\left\{I(X_D > X_{\bar{D}}\frac{1}{p_D}\frac{\partial p_D}{\partial \theta}\right\}\right. \\
&+ \left. 2AUC \times E\left\{\frac{1}{p_D}\frac{\partial p_D}{\partial \theta}\right\}^T I_D^{-1} E\left\{I(X_D > X_{\bar{D}}\frac{1}{p_D}\frac{\partial p_D}{\partial \theta}\right\} - AUC^2 E\left\{\frac{1}{p_D}\frac{\partial p_D}{\partial \theta}\right\}^T I_D^{-1} E\left\{\frac{1}{p_D}\frac{\partial p_D}{\partial \theta}\right\}\right] \\
&= -\frac{1}{1-\lambda}\left[AUC \times Q_{\bar{D}} - R_{\bar{D}}\right]^T I_{\bar{D}}^{-1}\left[AUC \times Q_{\bar{D}} - R_{\bar{D}}\right]
\end{aligned}
$$

where $Q_{\bar{D}} = E\left\{\frac{1}{p_{\bar{D}}}\frac{\partial p_{\bar{D}}}{\partial \theta}\right\}$ and $R_{\bar{D}} = E\left\{I(X_D > X_{\bar{D}})\frac{1}{p_{\bar{D}}}\frac{\partial p_{\bar{D}}}{\partial \theta}\right\}$. Similarly, we can show that

$$-\text{var}(D) - 2AUC\left\{\text{cov}(D,F) + \text{cov}(B,H) + \text{cov}(D,H)\right\} + AUC^2\left\{\text{var}(H) + 2\text{cov}(F,H)\right\}$$
$$= -\frac{1}{\lambda}\left[AUC \times Q_D - R_D\right]^T I_D^{-1}\left[AUC \times Q_D - R_D\right],$$

where $Q_D = E\left(\frac{1}{p_D}\frac{\partial p_D}{\partial \theta}\right)$, $R_D = E\left\{I(X_D > X_{\bar{D}})\frac{1}{p_D}\frac{\partial p_D}{\partial \theta}\right\}$.

Since I_D^{-1} and $I_{\bar{D}}^{-1}$ are positive definition, we have $\Sigma_x^{(III)} \leqslant \Sigma_x^{(I)}$ and $\Sigma_x^{(IV)} \leqslant \Sigma_x^{(II)}$. That is, the asymptotic variance of $\widetilde{AUC}_x(\hat{p})$ is smaller than or equal to the asymptotic variance of $\widetilde{AUC}_x(p)$, and the asymptotic variance of $\widehat{AUC}_x(\hat{p})$ is smaller than or equal to the asymptotic variance of $\widehat{AUC}_x(p)$. So estimating the sampling weight can lead to improvement in efficiency in Bernoulli sampling even if the sampling probability is known.

APPENDIX C. PROOFS OF THEOREM 2

We sketch the proofs for Theorem 2, which follow similar argument as the proofs of Theorem 1.

i) We can show that $\sqrt{n}\left\{\Delta\widetilde{AUC}(p) - \Delta AUC\right\} = (A_y - A_x) + (B_y - B_x) + o_p(1)$, which is asymptotically normally distributed with mean zero and variance $\text{var}(A_y - A_x) + \text{var}(B_y - B_x)$, where

$$\text{var}(A_y - A_x) = \text{var}(A_x) + \text{var}(A_y) - 2\text{cov}(A_x, A_y)$$
$$\text{var}(B_y - B_x) = \text{var}(B_x) + \text{var}(B_y) - 2\text{cov}(B_x, B_y),$$

and

$$\text{cov}(A_x, A_y) = \frac{N}{N_D}\text{cov}\left\{\frac{\delta_D}{p_D}F_{\bar{D}x}(X_D), \frac{\delta_D}{p_D}F_{\bar{D}y}(Y_D)\right\}$$
$$= \frac{N}{N_D}\left(\text{cov}\left[E\left\{\frac{\delta_D}{p_D}F_{\bar{D}x}(X_D)|\sigma_{N_D}\right\}, E\left\{\frac{\delta_D}{p_D}F_{\bar{D}y}(Y_D)|\sigma_{N_D}\right\}\right] + E\left[\text{cov}\left\{\frac{\delta_D}{p_D}F_{\bar{D}x}(X_D), \frac{\delta_D}{p_D}F_{\bar{D}y}(Y_D)|\sigma_{N_D}\right\}\right]\right)$$
$$= \frac{1}{\lambda}\left[\text{cov}\left\{F_{\bar{D}x}(X_D), F_{\bar{D}y}(Y_D)\right\} + E\left\{\frac{1-p_D}{p_D}F_{\bar{D}x}(X_D)F_{\bar{D}y}(Y_D)\right\}\right],$$
$$\text{cov}(B_x, B_y) = \frac{1}{1-\lambda}\left[\text{cov}\left\{S_{Dx}(X_{\bar{D}}), S_{Dy}(Y_{\bar{D}})\right\} + E\left\{\frac{1-p_{\bar{D}}}{p_{\bar{D}}}S_{Dx}(X_{\bar{D}})S_{Dy}(Y_{\bar{D}})\right\}\right].$$

ii) We have

$$\text{var}\left[\sqrt{N}\left\{\Delta\widehat{AUC}(p) - \Delta AUC\right\}\right]$$
$$\simeq \text{var}(A_y - A_x) + \text{var}(B_y - B_x) - 2\Delta AUC\left\{\text{cov}(A_y - A_x, E) + \text{cov}(B_y - B_x, F)\right\}$$
$$+ \Delta AUC^2\left\{\text{var}(E) + \text{var}(F)\right\}.$$

iii) We have

$$\sqrt{N}\left\{\Delta\widetilde{\widehat{AUC}}(\hat{p})-\Delta AUC\right\}\simeq(A_y-A_x)+(B_y-B_x)+(C_y-C_x)+(D_y-D_x).$$

As a result,

$$\mathrm{var}\left[\sqrt{N}\left\{\Delta\widetilde{\widehat{AUC}}(\hat{p})-\Delta AUC\right\}\right]\simeq\mathrm{var}(A_y-A_x)+\mathrm{var}(B_y-B_x)+\mathrm{var}(C_y-C_x)+\mathrm{var}(D_y-D_x)$$

$$+\,2\mathrm{cov}(A_y-A_x,C_y-C_x)+2\mathrm{cov}(B_y-B_x,D_y-D_x).$$

From similar argument as in single marker case, we have

$$\mathrm{cov}(C_x,C_y)=\frac{1}{\lambda}E\left\{I(X_D>X_{\bar{D}})\frac{1}{p_D}\frac{\partial p_D}{\partial\theta_D}\right\}^T I_D^{-1}E\left\{I(Y_D>Y_{\bar{D}})\frac{1}{p_D}\frac{\partial p_D}{\partial\theta_D}\right\}$$

$$\mathrm{cov}(D_x,D_y)=\frac{1}{1-\lambda}E\left\{I(X_D>X_{\bar{D}})\frac{1}{p_{\bar{D}}}\frac{\partial p_{\bar{D}}}{\partial\theta_{\bar{D}}}\right\}^T I_{\bar{D}}^{-1}E\left\{I(Y_D>Y_{\bar{D}})\frac{1}{p_{\bar{D}}}\frac{\partial p_{\bar{D}}}{\partial\theta_{\bar{D}}}\right\},$$

$\mathrm{cov}(B_x,D_y)=\mathrm{cov}(D_x,B_y)=-\mathrm{cov}(D_x,D_y)$, and $\mathrm{cov}(A_x,C_y)=\mathrm{cov}(C_x,A_y)=-\mathrm{cov}(C_x,C_y)$, which lead to $\mathrm{cov}(B_y-B_x,D_y-D_x)=-\mathrm{var}(D_y-D_x)$ and $\mathrm{cov}(A_y-A_x,C_y-C_x)=-\mathrm{var}(C_y-C_x)$. Therefore we have

$$\mathrm{var}\left[\sqrt{N}\left\{\Delta\widetilde{\widehat{AUC}}(\hat{p})-\Delta AUC\right\}\right]\simeq\mathrm{var}(A_y-A_x)+\mathrm{var}(B_y-B_x)-\mathrm{var}(C_y-C_x)-\mathrm{var}(D_y-D_x).$$

iv) We have

$$\mathrm{var}\left\{\sqrt{N}\left(\Delta\widehat{AUC}(\hat{p})-\Delta AUC\right)\right\}$$

$$\simeq\mathrm{var}(A_y-A_x)+\mathrm{var}(B_y-B_x)+\mathrm{var}(C_y-C_x)+\mathrm{var}(D_y-D_x)$$

$$+\,2\mathrm{cov}(A_y-A_x,C_y-C_x)+2\mathrm{cov}(B_y-B_x,D_x-D_y)$$

$$-\,2\Delta AUC\times\{\mathrm{cov}(A_y-A_x,E)+\mathrm{cov}(C_y-C_x,E)+\mathrm{cov}(B_y-B_x,F)+\mathrm{cov}(D_y-D_x,F)$$

$$+\,\mathrm{cov}(A_y-A_x,G)+\mathrm{cov}(C_y-C_x,G)+\mathrm{cov}(B_y-B_x,H)+\mathrm{cov}(D_y-D_x,H)\}$$

$$+\,\Delta AUC^2\times\{\mathrm{var}(E)+\mathrm{var}(F)+\mathrm{var}(G)+\mathrm{var}(H)+2\mathrm{cov}(E,G)+2\mathrm{cov}(F,H)\}$$

$$= \text{var}\left\{\sqrt{n}\left(\Delta\widehat{AUC}(p) - \Delta AUC\right)\right\} - \text{var}(C_y - C_x) - \text{var}(D_y - D_x)$$

$$- 2\Delta AUC \times \{\text{cov}(C_y - C_x, E) + \text{cov}(D_y - D_x, F) + \text{cov}(A_y - A_x, G) + \text{cov}(C_y - C_x, G)$$

$$+ \text{cov}(B_y - B_x, H) + \text{cov}(D_y - D_x, H)\}$$

$$+ \Delta AUC^2 \times \{\text{var}(G) + \text{var}(H) + 2\text{cov}(E, G) + 2\text{cov}(F, H)\}$$

$$= \text{var}\left\{\sqrt{n}\left(\Delta\widehat{AUC}(p) - \Delta AUC\right)\right\} - \frac{1}{\lambda}\left[\Delta AUC \times Q_D - (R_{Dy} - R_{Dx})\right]^T I_D^{-1}\left[\Delta AUC \times Q_D - (R_{Dy} - R_{Dx})\right]$$

$$- \frac{1}{1-\lambda}\left[\Delta AUC \times Q_{\bar{D}} - (R_{\bar{D}y} - R_{\bar{D}x})\right]^T I_{\bar{D}}^{-1}\left[\Delta AUC \times Q_{\bar{D}} - (R_{\bar{D}y} - R_{\bar{D}x})\right].$$

Again, we can see that asymptotic variance of $\Delta\widetilde{AUC}(\hat{p})$ is less than or equal to the asymptotic variance of $\Delta\widetilde{AUC}(p)$ and asymptotic variance of $\Delta\widehat{AUC}(\hat{p})$ is less than or equal to the asymptotic variance of $\Delta\widehat{AUC}(p)$.

Appendix D. Finite-Population Stratified Sampling

Here we demonstrate the equivalence in asymptotic variance of $\widetilde{AUC}(\hat{p})$ between Bernoulli sampling and finite-population stratified sampling when $\hat{p}$ is estimated from discrete stratum and when sampling fraction for cases and controls in each stratum in finite-population stratified sampling converges to the corresponding sampling probability in Bernoulli sampling.

In particular, suppose there are K_D strata among cases with stratum indicator $W_D = w_{D1}, \ldots, w_{DK_D}$ and $K_{\bar{D}}$ strata among controls with stratum indicator $W_{\bar{D}} = w_{\bar{D}1}, \ldots, w_{\bar{D}K_{\bar{D}}}$. Let $N_{Dk_D}, k_D = 1, \ldots, K_D$ be number of cases in stratum k_D and $N_{\bar{D}k_{\bar{D}}}, k_{\bar{D}} = 1, \ldots, K_{\bar{D}}$ be number of controls in stratum $k_{\bar{D}}$ in the phase-one cohort sample. In second phase of the study, we sample fixed number n_{Dk_D} of cases without replacement from stratum k_D and sampling fixed number $n_{\bar{D}k_{\bar{D}}}$ of controls without replacement from stratum $k_{\bar{D}}$. Suppose as $N \to \infty$, the sampling fractions for cases among stratum $k_D \in (1, \ldots, K_D)$ converge with $n_{Dk_D}/N_{Dk_D} \to \pi_{Dk_D} \in (0, 1]$, and the sampling fractions for controls among stratum $k_{\bar{D}} \in (1, \ldots, K_{\bar{D}})$ converge with $n_{\bar{D}k_{\bar{D}}}/N_{\bar{D}k_{\bar{D}}} \to \pi_{\bar{D}k_{\bar{D}}} \in (0, 1]$

Here we use subscript ${}_{Dk_D,i}$ to indicate the ith cases among stratum k_D, and use subscript ${}_{\bar{D}k_{\bar{D}},j}$ to indicate the jth controls among stratum $k_{\bar{D}}$. We have

$$
\begin{aligned}
&\sqrt{N}\left\{\widetilde{\widehat{AUC}}(\hat{p}) - AUC\right\} \\
&= \sqrt{N}\left\{\frac{1}{N_D}\frac{1}{N_{\bar{D}}}\sum_{k_{\bar{D}}=1}^{K_{\bar{D}}}\sum_{k_D=1}^{K_D}\sum_{j=1}^{N_{\bar{D}k_{\bar{D}}}}\sum_{i=1}^{N_{Dk_D}}\frac{\delta_{\bar{D}k_{\bar{D}},j}}{\hat{\pi}_{\bar{D}k_{\bar{D}}}}\frac{\delta_{Dk_D,i}}{\hat{\pi}_{Dk_D}}I\left(X_{Dk_D,i} > X_{\bar{D}k_{\bar{D}},j}\right) - \frac{1}{N_{\bar{D}}}\sum_{k_{\bar{D}}=1}^{K_{\bar{D}}}\sum_{j=1}^{N_{\bar{D}k_{\bar{D}}}}\frac{\delta_{\bar{D}k_{\bar{D}},j}}{\hat{\pi}_{\bar{D}k_{\bar{D}}}}S_D(X_{\bar{D}k_{\bar{D}},j})\right\} \\
&+ \sqrt{N}\left[\frac{1}{N_{\bar{D}}}\sum_{k_{\bar{D}}=1}^{K_{\bar{D}}}\sum_{j=1}^{N_{\bar{D}k_{\bar{D}}}}\frac{\delta_{k_{\bar{D}},j}}{\hat{\pi}_{\bar{D}k_{\bar{D}}}}S_D(X_{\bar{D}k_{\bar{D}},j}) - E\{S_D(X_{\bar{D}})\}\right] \\
&= AA + BB + o_p(1),
\end{aligned}
$$

where

$$
AA = \sqrt{N}\left\{\frac{1}{N_D}\sum_{k_D=1}^{K_D}\frac{\delta_{Dk_D,i}}{\hat{\pi}_{Dk_D}}F_{\bar{D}}(X_{Dk_D,i}) - E\{F_{\bar{D}}(X_D)\}\right\},
$$

$$
BB = \sqrt{N}\left\{\frac{1}{N_{\bar{D}}}\sum_{k_{\bar{D}}=1}^{K_{\bar{D}}}\frac{\delta_{\bar{D}k_{\bar{D}},j}}{\hat{\pi}_{\bar{D}k_{\bar{D}}}}S_D(X_{\bar{D}k_{\bar{D}},j}) - E\{S_D(X_{\bar{D}})\}\right\}.
$$

Next we derive the asymptotic variance of AA. The derivation follows the strategy as in Breslow and Wellner (2007). The asymptotic variance of BB can be derived following similar arguments.

Let $\mathbb{P}^{\pi}_{N_D}$ be the IPW empirical measure of cases.

$$
\mathbb{P}^{\pi}_{N_D} = \frac{1}{N_D}\sum_{k_D=1}^{K_D}\frac{N_{Dk_D}}{n_{Dk_D}}\sum_{i=1}^{N_{Dk_D}}\delta_{Dk_D,i}\zeta_{X_{Dk_D,i}} = \frac{1}{N_D}\sum_{k_D=1}^{K_D}\frac{N^2_{Dk_D}}{n_{Dk_D}}\mathbb{P}^{\delta}_{k_D,N_{Dk_D}},
$$

where $\mathbb{P}^{\delta}_{k_D,N_{Dk_D}} = \sum_{j=1}^{N_{Dk_D}}\delta_{Dk_D,j}\zeta_{X_{Dk_D,i}}/N_{Dk_D}$ is a 'finite sampling empirical measure' for stratum k_D among cases and $\zeta_{X_{Dk_D,i}}$ is the Dirac measure placing unit mass on $X_{Dk_D,i}$. Let $\mathbb{P}_{N_D}$ denote the empirical measure of cases:

$$
\mathbb{P}_{N_D} = \frac{1}{N_D}\sum_{k_D=1}^{K_D}N_{Dk_D}\frac{1}{N_{Dk_D}}\sum_{j=1}^{N_{Dk_D}}\zeta_{X_{Dk_D,i}} = \frac{1}{N_D}\sum_{k_D=1}^{K_D}N_{Dk_D}\mathbb{P}_{k_D,N_{Dk_D}},
$$

where $\mathbb{P}_{k_D,N_{Dk_D}} = \sum_{i=1}^{N_{Dk_D}}\zeta_{X_{Dk_D,i}}/N_{Dk_D}$. Let P_{D_0} denote the true marker distribution among cases. Let $\mathbb{G}_{N_D} = \sqrt{N_D}\,(\mathbb{P}_{N_D} - P_{D_0})$ denote the standard empirical process for cases. We have

$$
\begin{aligned}
\mathbb{G}^{\pi}_{N_D} &= \sqrt{N_D}\left(\mathbb{P}^{\pi}_{N_D} - P_{D_0}\right)\\
&= \sqrt{N_D}\left(\mathbb{P}_{N_D} - P_{D_0}\right) + \sqrt{N_D}\left(\mathbb{P}^{\pi}_{N_D} - \mathbb{P}_{N_D}\right)\\
&= \mathbb{G}_{N_D} + \frac{1}{\sqrt{N_D}}\sum_{k_D=1}^{K_D}\frac{N^2_{Dk_D}}{n_{Dk_D}}\left(\mathbb{P}^{\delta}_{k_D,N_{Dk_D}} - \frac{n_{Dk_D}}{N_{Dk_D}}\mathbb{P}_{k_D,N_{Dk_D}}\right)\\
&= \mathbb{G}_{N_D} + \sum_{k_D=1}^{K_D}\sqrt{\frac{N_{Dk_D}}{n_{Dk_D}}}\frac{N_{Dk_D}}{n_{Dk_D}}\mathbb{G}^{\delta}_{k_D,N_{Dk_D}},
\end{aligned}
$$

where $\mathbb{G}^{\delta}_{k_D,N_{Dk_D}} = \sqrt{N_{Dk_D}}\left(\mathbb{P}^{\delta}_{k_D,N_{Dk_D}} - \frac{n_{Dk_D}}{N_{Dk_D}}\mathbb{P}_{k_D,N_{Dk_D}}\right)$ is the 'finite sampling empirical process' for stratum k_D among cases.

If $n_{Dk_D}/N_{Dk_D} \to \pi_{Dk_D}$, then $\sum_{k_D=1}^{K_D}\left(\delta_{Dk_D,j} - \bar{\delta}_{Dk_D}.\right)^2/N_{Dk_D} \to^p \pi_{Dk_D}(1-\pi_{Dk_D})$. Furthermore, with $\rightsquigarrow$ denoting weak convergence in $l^{\infty}(\mathcal{F})$, we have $\sqrt{N_{Dk_D}}\left(\mathbb{P}_{k_D,N_{Dk_D}} - P_{D_0|k_D}\right) \rightsquigarrow \mathbb{G}_{Dk_D}$, where $P_{D_0|k_D}$ denote P_{D_0} conditional on membership in stratum k_D, as shown in Breslow and Wellner (2007). Thus according to theorem 3.6.13 and 1.12.4 in Van der Vaart and Wellner (1996), for almost every sequence of complete data, $\mathbb{G}^{\delta}_{k_D,N_{Dk_D}} \rightsquigarrow \sqrt{\pi_{Dk_D}(1-\pi_{Dk_D})}\mathbb{G}_{Dk_D}$, where $\mathbb{G}_{Dk_D}$ denote the $P_{D_0|k_D}$ Brownian Bridge. Let σ_{N_D} be the sigma field of information, potentially available for N_D cases. Conditional on σ_{N_D}, the process $\mathbb{G}^{\delta}_{k_D,N_{k_D}}$ are mutually independent because of the independence of the $\{\delta_{Dk_D,j}\}$ in different strata. Furthermore, they are (unconditionally) uncorrelated with $\mathbb{G}_{N_D} = \sqrt{N_D}(\mathbb{P}_{N_D} - P_0)$, following corollary 2.9.3 of Van der Vaart and Wellner (1996) and Breslow and Wellner (2007). The vector of processes $(\mathbb{G}_{N_D}, \mathbb{G}^{\delta}_{1,N_{D1}}, \ldots, \mathbb{G}^{\delta}_{K_D,N_{DK_D}})$ converges weakly to the vector of independent Brownian bridge processes $(\mathbb{G}, \mathbb{G}^{\delta}_1, \ldots, \mathbb{G}^{\delta}_{K_D})$, which leads to

$$
\mathbb{G}^{\pi}_{N_D} \rightsquigarrow \mathbb{G} + \sum_{k_D=1}^{K_D}\sqrt{P(W_D = w_{k_D})}\sqrt{\frac{1-\pi_{Dk_D}}{\pi_{Dk_D}}}\mathbb{G}_{Dk_D},
$$

by continuous mapping theorem. Therefore,

$$
\operatorname{var}(AA) \simeq \frac{1}{\lambda}\left[\operatorname{var}\{F_{\bar{D}}(X_D)\} + \sum_{k_D=1}^{K_D}P(W_D = w_{k_D})\frac{1-\pi_{Dk_D}}{\pi_{Dk_D}}\operatorname{var}\left\{F_{\bar{D}}(X_{Dk_D})\right\}\right]. \qquad (0.4)
$$

Now look at the asymptotic variance of $\widehat{AUC}(\hat{p})$ or equivalently $\widetilde{AUC}(\hat{p})$ in Bernoulli sam-

pling where $\hat{p}_{Dk_D}$ and $\hat{p}_{\bar{D}k_{\bar{D}}}$ are empirical estimates from each stratum. From Appendix B2, $\sqrt{N}\left\{\widehat{AUC}(\hat{p}) - AUC\right\} = \mathrm{var}(A) - \mathrm{var}(C) + \mathrm{var}(B) - \mathrm{var}(D)$, where $\mathrm{var}(A) - \mathrm{var}(C)$ is variability contributed by cases and $\mathrm{var}(B) - \mathrm{var}(D)$ is variability contributed by controls. We have

$$\mathrm{var}(C) = \frac{1}{\lambda}\sum_{k=1}^{K_D}\left[E\left\{I(X_D > X_{\bar{D}})I(W_D = w_{Dk_D})\frac{1}{\pi_{Dk_D}}\right\}\right]^2\left\{\left(\frac{1}{\pi_{Dk_D}} + \frac{1}{1-\pi_{Dk_D}}\right)P(W_D = w_{Dk_D})\right\}^{-1}$$

$$= \frac{1}{\lambda}\sum_{k=1}^{K_D}\frac{1-\pi_{Dk_D}}{\pi_{Dk_D}}P(W_D = w_{Dk_D})P(X_D > X_{\bar{D}}|W_D = w_{Dk_D})^2 = \frac{1}{\lambda}\sum_{k=1}^{K_D}\frac{1-\pi_{Dk_D}}{\pi_{Dk_D}}P(W_D = w_{Dk_D})E\{F_{X_{\bar{D}}}(X_{Dk_D})\}^2,$$

and therefore,

$$\begin{aligned}
&\mathrm{var}(A) - \mathrm{var}(C)\\
&= \frac{1}{\lambda}\left(\mathrm{var}\{F_{\bar{D}}(X_D) + E\left\{\frac{p_D(1-p_D)}{p_D^2}F_{\bar{D}}(X_D)^2\right\} - \sum_{k=1}^{K_D}P(W_D = w_{Dk_D})\frac{1-\pi_{Dk_D}}{\pi_{Dk_D}}E\{F_{X_{\bar{D}}}(X_{Dk})\}^2\right)\\
&= \frac{1}{\lambda}\left[\mathrm{var}\{F_{\bar{D}}(X_D) + \sum_{k_D=1}^{K_D}P(W_D = w_{Dk_D})\frac{1-\pi_{Dk_D}}{\pi_{Dk_D}}E\{F_{\bar{D}}(X_{Dk_D})^2\}\right.\\
&\left.-\sum_{k=1}^{K_D}P(W_D = w_{Dk_D})\frac{1-\pi_{Dk_D}}{\pi_{Dk_D}}E\{F_{X_{\bar{D}}}(X_{Dk_D})\}^2\right]\\
&= \frac{1}{\lambda}\left[\mathrm{var}\{F_{\bar{D}}(X_D)\} + \sum_{k_D=1}^{K_D}P(W_D = w_{Dk_D})\frac{1-\pi_{Dk_D}}{\pi_{Dk_D}}\mathrm{var}\{F_{\bar{D}}(X_{Dk_D})\}.\right],
\end{aligned}$$

which equals (0.4).

Appendix E. Comparing variance of $\widetilde{AUC}(p)$ and $\widehat{AUC}(p)$ in Bernoulli Sampling With Discrete Strata

Consider Bernoulli sampling design where $\hat{\pi}_{Dk_D}$ and $\hat{\pi}_{\bar{D}k_{\bar{D}}}$ are empirical estimates of phase-two sampling proportion from each case and control stratum. From appendix B2, analytical variance of $\sqrt{N}\left\{\widetilde{AUC}_x(p) - AUC_x\right\}$ minus analytical variance of $\sqrt{N}\left\{\widehat{AUC}_x(p) - AUC_x\right\}$ equals

$$\begin{aligned}
AUC_x \times \frac{1}{\lambda}&\left[2\sum_{k_D=1}^{K_D}P(W_D = w_{Dk_D})\left(\frac{1}{\pi_{Dk_D}} - 1\right)E\left\{F_{X_{\bar{D}}}(X_{Dk_D})\right\}\right.\\
&\left.-\sum_{k_D=1}^{K_D}P(W_D = w_{Dk_D})\left(\frac{1}{\pi_{Dk_D}} - 1\right)\sum_{k_D=1}^{K_D}P(W_D = w_{Dk_D})E\left\{F_{X_{\bar{D}}}(X_{Dk_D})\right\}\right]
\end{aligned} \tag{0.5}$$

$$+ AUC_x \times \frac{1}{1-\lambda}\left[2\sum_{k_{\bar{D}}=1}^{K_{\bar{D}}} P(W_{\bar{D}} = w_{\bar{D}k_{\bar{D}}})\left(\frac{1}{\pi_{\bar{D}k_{\bar{D}}}} - 1\right) E\left\{S_{X_D}(X_{\bar{D}k_{\bar{D}}})\right\}\right.$$
$$\left. - \sum_{k_{\bar{D}}=1}^{K_{\bar{D}}} P(W_{\bar{D}} = w_{\bar{D}k_{\bar{D}}})\left(\frac{1}{\pi_{\bar{D}k_{\bar{D}}}} - 1\right) \times \sum_{k_{\bar{D}}=1}^{K_{\bar{D}}} P(W_{\bar{D}} = w_{\bar{D}k_{\bar{D}}}) E\left\{S_{X_D}(X_{\bar{D}k_{\bar{D}}})\right\}\right], \qquad (0.6)$$

where (0.5) and (0.6) are variance components due to variability of cases and controls respectively.

Similarly, analytical variance of $\sqrt{N}\left\{\Delta\widetilde{AUC}(p) - \Delta AUC\right\}$ minus that of $\sqrt{N}\left\{\Delta\widehat{AUC}(p) - \Delta AUC\right\}$ equals

$$\Delta AUC \times \frac{1}{\lambda}\left[2\sum_{k_D=1}^{K_D} P(W_D = w_{Dk_D})\left(\frac{1}{\pi_{Dk_D}} - 1\right)\left[E\left\{F_{Y_{\bar{D}}}(Y_{Dk_D})\right\} - E\left\{F_{X_{\bar{D}}}(X_{Dk_D})\right\}\right]\right.$$
$$\left. - \sum_{k_D=1}^{K_D} P(W_D = w_{Dk_D})\left(\frac{1}{\pi_{Dk_D}} - 1\right)\sum_{k_D=1}^{K_D} P(W_D = w_{Dk_D}) \times \left[E\left\{F_{Y_{\bar{D}}}(Y_{Dk_D})\right\} - E\left\{F_{X_{\bar{D}}}(X_{Dk_D})\right\}\right]\right]$$
$$+ \Delta AUC \times \frac{1}{1-\lambda}\left[2\sum_{k_{\bar{D}}=1}^{K_{\bar{D}}} P(W_{\bar{D}} = w_{\bar{D}k_{\bar{D}}})\left(\frac{1}{\pi_{\bar{D}k_{\bar{D}}}} - 1\right)\left[E\left\{S_{Y_D}(Y_{\bar{D}k_{\bar{D}}})\right\} - E\left\{S_{X_D}(X_{\bar{D}k_{\bar{D}}})\right\}\right]\right.$$
$$\left. - \sum_{k_{\bar{D}}=1}^{K_{\bar{D}}} P(W_{\bar{D}} = w_{\bar{D}k_{\bar{D}}})\left(\frac{1}{\pi_{\bar{D}k_{\bar{D}}}} - 1\right) \times \sum_{k_{\bar{D}}=1}^{K_{\bar{D}}} P(W_{\bar{D}} = w_{\bar{D}k_{\bar{D}}}) \times \left[E\left\{S_{Y_D}(Y_{\bar{D}k_{\bar{D}}})\right\} - E\left\{S_{X_D}(X_{\bar{D}k_{\bar{D}}})\right\}\right]\right]$$

We take a close look at (0.6) since results about (0.5) follow similarly. We made the following observations: i) oftentimes decreasing sampling probability for controls tends to increase (0.6); ii) (0.6) will be non-negative if $1/p_{\bar{D}} - 1$ and $E\left\{S_{X_D}(X_{\bar{D}k_{\bar{D}}})\right\}$ are independent or positively correlated; iii) there exist scenarios where (0.6) is negative. For example, if $AUC_x = 0.664$, $\pi_{Dk_{\bar{D}}} = 0.9, 0.1$ for $k_{\bar{D}} = 1, 2$, $E\left\{S_{X_D}(X_{\bar{D}k_{\bar{D}}})\right\} = (0.254, 0.701)$ for $k_{\bar{D}} = 1, 2$, then the term in (0.6) equals -0.0479. In general, we found $\widehat{AUC}(p)$ to be more efficient or have very similar variability compared to $\widetilde{AUC}(p)$. Supplementary Figure 1 explored the magnitude of (0.6) as a function of several parameters for the following setting.

Appendix E1: Setting for Supplementary Figures 1 and 2

We consider binary disease D with prevalence $\lambda = 0.1$. Biomarker X and covariate W^* are jointly normally distributed conditional on D, with $\rho_{xw^*} = 0.5$ conditional on D. Among controls, X and W^* each follows a standard normal distribution. Among cases, X follows $N(1,1)$ and W follows $N(0.6,1)$. We have $AUC_x = 0.76$. Let W be a discrete covariate stratum derived from W^*, which

takes two levels: $W = 1$ if $W^* < \Phi^{-1}(\eta)$, and $W = 2$ if $W^* > \Phi^{-1}(1-\eta)$, for $\eta \in (0,1)$, where Φ is the CDF of standard normal. Consider data from two-phase studies. In the first phase, $N = 5,000$ subjects are randomly sampled from the population, whose D and W values are measured. In the second phase, Bernoulli sampling of cases and controls are performed for measuring marker X stratified on strata W, assuming on average $n_{\bar{D}} = 250$ controls are sampled from the two strata. Note $\eta = P(W = 1|D = 0)$ is the probability a control in the population is from stratum 1, and let $\alpha = P(W = 1|D = 0, \text{Sampled in phase two})$ be the proportion of phase-two control samples from stratum 1. We have $\pi_{\bar{D}1} = n_{\bar{D}} \times \alpha/(N \times (1-\lambda) \times \eta)$ and $\pi_{\bar{D}2} = n_{\bar{D}} \times (1-\alpha)/(N \times (1-\lambda) \times (1-\eta))$.

In Supplementary Figure 1, we plot (0.6) as function of η, α, and ρ^*_{xw} individually while keeping the other two factors constant. It appears that (0.6) tends to increase with η or α getting closer to 0 or 1.

Appendix F. Variance reduction due to weight estimation in Bernoulli Sampling from Discrete Strata

Consider Bernoulli sampling design where $\hat{\pi}_{Dk_D}$ and $\hat{\pi}_{\bar{D}k_{\bar{D}}}$ are empirical estimates of phase-two sampling proportion from each case and control stratum.

(I) From Appendix B3, reduction in analytical variance of $\sqrt{N}\left\{\widetilde{AUC}_x(\hat{p}) - AUC_x\right\}$ compared to variance of $\sqrt{N}\left\{\widetilde{AUC}_x(p) - AUC_x\right\}$ is $\text{var}(C_x) + \text{var}(D_x)$, where

$$\text{var}(C_x) = \frac{1}{\lambda}\sum_{k_D=1}^{K_D}\frac{1-\pi_{Dk_D}}{\pi_{Dk_D}}P(W_D = w_{Dk_D})E\{F_{X_{\bar{D}}}(X_{Dk_D})\}^2,$$

$$\text{var}(D_x) = \frac{1}{1-\lambda}\sum_{k_{\bar{D}}=1}^{K_{\bar{D}}}\frac{1-\pi_{\bar{D}k_{\bar{D}}}}{\pi_{\bar{D}k_{\bar{D}}}}P(W_{\bar{D}} = w_{\bar{D}k_{\bar{D}}})E\{S_{X_D}(X_{\bar{D}k_{\bar{D}}})\}^2.$$

Similarly, reduction in analytical variance of $\sqrt{N}\left\{\Delta\widetilde{AUC}(\hat{p}) - \Delta AUC\right\}$ compared to variance of $\sqrt{N}\left\{\Delta\widetilde{AUC}(p) - \Delta AUC\right\}$ is $\text{var}(C_y - C_x) + \text{var}(D_y - D_x)$, where

$$\text{var}(C_y - C_x) = \frac{1}{\lambda}\sum_{k_D=1}^{K_D}\frac{1-\pi_{Dk_D}}{\pi_{Dk_D}}P(W_D = w_{Dk_D})\left(E\{F_{Y_{\bar{D}}}(Y_{Dk_D})\} - E\{F_{X_{\bar{D}}}(X_{Dk_D})\}\right)^2,$$

$$\text{var}(D_y - D_x) = \frac{1}{1-\lambda} \sum_{k_{\bar{D}}=1}^{K_{\bar{D}}} \frac{1-\pi_{\bar{D}k_{\bar{D}}}}{\pi_{\bar{D}k_{\bar{D}}}} P(W_{\bar{D}} = w_{\bar{D}k_{\bar{D}}}) \left(E\{S_{Y_D}(Y_{\bar{D}k_{\bar{D}}})\} - E\{S_{X_D}(X_{\bar{D}k_{\bar{D}}})\} \right)^2 .$$

Note that analytic variance reduction due to weights estimation for $\widetilde{AUC}$ or $\Delta\widetilde{AUC}$ can be represented as a weighted sum of squared terms across strata; the term for each stratum is the AUC comparing cases in this particular stratum with a random control or vice versa for estimation of AUC, or the difference in these AUCs between markers for estimation of ΔAUC. The latter can be much smaller in magnitude since AUC difference is smaller than AUC itself.

(II) From Appendix B4, reduction in analytical variance of $\sqrt{N}\left\{\widehat{AUC}_x(\hat{p}) - AUC_x\right\}$ compared to variance of $\sqrt{N}\left\{\widehat{AUC}_x(p) - AUC_x\right\}$ equals the sum of following two terms:

$$\frac{1}{\lambda} \sum_{k_D=1}^{K_D} \frac{1-\pi_{Dk_D}}{\pi_{Dk_D}} P(W_D = w_{Dk_D}) \left(AUC_x - E\{F_{X_{\bar{D}}}(X_{Dk_D})\} \right)^2 , \tag{0.7}$$

$$\frac{1}{1-\lambda} \sum_{k_{\bar{D}}=1}^{K_{\bar{D}}} \frac{1-\pi_{\bar{D}k_{\bar{D}}}}{\pi_{\bar{D}k_{\bar{D}}}} P(W_{\bar{D}} = w_{\bar{D}k_{\bar{D}}}) \left(AUC_x - E\{S_{X_D}(X_{\bar{D}k_{\bar{D}}})\} \right)^2 . \tag{0.8}$$

Similarly, reduction in analytical variance of $\sqrt{N}\left\{\Delta\widehat{AUC}(\hat{p}) - \Delta AUC\right\}$ compared to variance of $\sqrt{N}\left\{\Delta\widehat{AUC}(p) - \Delta AUC\right\}$ equals the sum of following two terms:

$$\frac{1}{\lambda} \sum_{k_D=1}^{K_D} \frac{1-\pi_{Dk_D}}{\pi_{Dk_D}} P(W_D = w_{Dk_D}) \left[AUC_y - AUC_x - \left(E\{F_{Y_{\bar{D}}}(Y_{Dk_D})\} - E\{F_{X_{\bar{D}}}(X_{Dk_D})\} \right) \right]^2 ,$$

$$\frac{1}{1-\lambda} \sum_{k_{\bar{D}}=1}^{K_{\bar{D}}} \frac{1-\pi_{\bar{D}k_{\bar{D}}}}{\pi_{\bar{D}k_{\bar{D}}}} P(W_{\bar{D}} = w_{\bar{D}k_{\bar{D}}}) \left[AUC_y - AUC_x - \left(E\{S_{Y_D}(Y_{\bar{D}k_{\bar{D}}})\} - E\{S_{X_D}(X_{\bar{D}k_{\bar{D}}})\} \right) \right]^2 .$$

We take a close look at (0.8), i.e. reduction in asymptotic variance of $\widehat{AUC}(p)$ attributed to variability of controls by estimating sampling probability. Note that (0.8) tends to be big if there exist some large strata $k_{\bar{D}}$ with small sampling probability and if there is large difference between $E\{S_{X_D}(X_{\bar{D}k_{\bar{D}}})\}$ and AUC_x. Using the same setting as presented in Supplementary Appendix E1, we plot (0.8) as function of $\eta = P(W=1|D=0)$, $\alpha = P(W=1|D=0, \text{Sampled in phase two})$, and $\rho^*_{xw} = cor(X, W^*|D=0)$ individually while keeping the other two factors constant. In

general, we see a U-shape for (0.8) as functions of α or ρ_{xw^*} and a upside down U-shape for (0.8) as a function of η (Supplementary Figure 2).

Appendix G. Expanded Tables for Simulation Studies with bi-binormal marker

This section present expanded tables for simulation studies described in Section 3 of the main text by including $\widetilde{AUC}(p)$ and $\widehat{AUC}(p)$ in addition to $\widehat{AUC}(\hat{p})$ and $\widehat{AUC}^{em}$.

Supplementary Table 1 shows performance of various estimators of AUC_x. Supplementary Table 2 shows performance of various estimators of ΔAUC for scenarios where the two markers have same variability and same correlation with covariate W^* conditional on D; Supplementary Table 3 shows performance of various estimators of ΔAUC for scenarios where the two markers have same variability but different correlation with covariate W^* conditional on D; Supplementary Table 4 shows performance of various estimators of ΔAUC for scenarios the two markers have different variability among cases.

Appendix H. Results for Biomarkers following Bi-gamma Model

Appendix H1. Simulations studies comparing various estimators

We consider a binary disease outcome D with prevalence $P(D = 1) = 0.1$ in the population. Let W^* be a continuous covariate, which belongs to standard normal among controls ($D = 0$) and belongs to $N(0.6, 1)$ among cases ($D = 1$). Let W be a discrete covariate stratum derived from W^*, which takes three levels: $W = 1$ if $W^* < \Phi^{-1}(1/3)$, $W = 2$ if $\Phi^{-1}(1/3) \leqslant W^* \leqslant \Phi^{-1}(2/3)$, and $W = 3$ if $W^* > \Phi^{-1}(2/3)$, where Φ is the CDF of standard normal.

We consider two biomarkers X and Y, each follows gamma distribution conditional on D (Dorfman *and others*, 1997). We assume shape parameter for each marker is equal between cases and controls, denoted as κ_x and κ_y for X and Y. Scale parameters for X and Y are σ_{Dx} and σ_{Dy} among cases and equal to 1 among controls. Let ρ_{xy}, ρ_{xw^*}, and ρ_{yw^*} be correlations between X

and Y, between X and W^* and between Y and W^* respectively conditional on D. We simulate the joint distribution of X, Y, W^* conditional on D using Gaussian copula model (Nelsen, 2013).

The sampling scheme for Monte-Carlo simulation studies is the same as that in Section 3 of the main text. Supplementary Table 9 shows performance of various estimators of AUC_x. Supplementary Table 10 shows performance of various estimators of ΔAUC for scenarios where the two marker have the same shape parameter and same correlation with covariate conditional on D; Supplementary Table 11 shows performance of various estimators of ΔAUC for scenarios where the two marker have the same shape parameter but different correlation with covariate conditional on D; Supplementary Table 12 shows performance of various estimators of ΔAUC for scenarios where the two markers have different shape parameters among cases.

Appendix H2. Implication on efficiency of sampling scheme for biomarkers following bi-gamma model

Consider a binary disease outcome D with prevalence $P(D = 1) = 0.1$ in the population. Let W^* be a continuous covariate, which belongs to standard normal among controls ($D = 0$) and belongs to $N(0.6, 1)$ among cases ($D = 1$). Let W be a discrete covariate stratum derived from W^*, which takes two levels: $W = 1$ if $W^* < \Phi^{-1}(1/2)$, $W = 2$ otherwise. Suppose marker X follows gamma distribution conditional on D, with shape parameter $1/3$, scale parameter 1 among controls, and scale parameter σ_{Dx} among cases. Correlation between X and W^* is ρ_{x,w^*} conditional on D.

We compare two sampling designs. Both are two-phase studies with a random cohort sample of size N drawn in the first phase. In the second phase, both designs include all cases from phase-one sample, *i.e.*, $\pi_D = 1$; a simple random sample of controls of size $n_{\bar{D}} = n_D$ are drawn without replacement in Design 1, simple random samples of controls with the same number as cases are drawn without replacement from each W stratum in Design 2. Empirical estimator of AUC is constructed using the biomarker samples in Design 1. The $\widehat{AUC}(\hat{p})$ with empirically estimated

sampling weights conditional on sampling strata is computed for Design 2. Supplementary Figure 3 shows the relative asymptotic efficiency of $\widehat{AUC}(\hat{p})$ in Design 2 versus $\widehat{AUC}^{em}$ in Design 1 for two different AUC_x values, as the correlation ρ_{x,w^*} changes.

Table 1: Performance of different AUC_x estimators for the bi-normal marker model described in Section 3. Disease prevalence is 0.1. Biomarker X is standard normal among controls. n_D and $n_{\bar{D}}$ indicate expected number of cases and controls sampled in phase two for Bernoulli sampling and exact number of cases and controls sampled for finite-population stratified sampling. Results are based on 5,000 Monte-Carlo Simulations.

					Bernoulli Sampling				*FPS sampling	
μ_{Dx}	σ_{Dx}	AUC_x	ρ^*_{xw}	$n_D = n_{\bar{D}}$	$\widetilde{AUC}_x(p)$	$\widehat{AUC}_x(p)$	$\widehat{AUC}_x(\hat{p}) = \widetilde{AUC}_x(\hat{p})$	$\widehat{AUC}_x^{em}$	$\widehat{AUC}_x(\hat{p})$	$\widehat{AUC}_x^{em}$
							Bias×100			
0.00	1.00	0.50	0.30	100	-0.01	-0.10	-0.06	-3.89	-0.05	-3.90
				250	-0.11	-0.02	0.00	-3.85	-0.04	-3.87
				400	-0.09	-0.00	0.01	-3.84	-0.04	-3.89
			0.50	100	0.01	-0.08	-0.01	-6.51	-0.14	-6.63
				250	-0.14	-0.07	-0.03	-6.54	-0.09	-6.58
				400	-0.15	-0.07	-0.04	-6.56	-0.04	-6.55
0.00	1.50	0.50	0.30	100	0.05	-0.01	0.01	-2.99	-0.06	-3.08
				250	-0.13	-0.03	-0.01	-3.03	-0.07	-3.08
				400	-0.14	-0.04	-0.04	-3.05	-0.05	-3.05
			0.50	100	0.04	-0.04	0.02	-5.05	-0.08	-5.17
				250	-0.13	-0.06	-0.03	-5.10	-0.08	-5.14
				400	-0.15	-0.07	-0.05	-5.12	-0.03	-5.12
0.60	1.00	0.664	0.30	100	0.01	-0.08	-0.05	-3.57	-0.05	-3.58
				250	-0.15	-0.02	0.00	-3.54	-0.04	-3.57
				400	-0.13	0.00	0.01	-3.53	-0.05	-3.59
			0.50	100	0.01	-0.08	-0.02	-6.01	-0.11	-6.10
				250	-0.16	-0.06	-0.02	-6.04	-0.08	-6.08
				400	-0.18	-0.07	-0.04	-6.05	-0.05	-6.05
0.765	1.50	0.664	0.30	100	0.08	0.01	0.03	-2.73	-0.06	-2.83
				250	-0.15	-0.01	0.00	-2.77	-0.07	-2.82
				400	-0.17	-0.04	-0.03	-2.80	-0.04	-2.80
			0.50	100	0.05	-0.02	0.03	-4.63	-0.08	-4.76
				250	-0.15	-0.04	-0.01	-4.68	-0.07	-4.74
				400	-0.18	-0.07	-0.05	-4.72	-0.02	-4.71
1.00	1.00	0.76	0.30	100	0.03	-0.06	-0.04	-3.05	-0.05	-3.07
				250	-0.18	-0.02	0.00	-3.03	-0.04	-3.05
				400	-0.15	0.00	0.01	-3.02	-0.06	-3.07
			0.50	100	0.01	-0.07	-0.02	-5.14	-0.09	-5.20
				250	-0.18	-0.05	-0.02	-5.16	-0.07	-5.20
				400	-0.19	-0.06	-0.04	-5.17	-0.05	-5.18
1.275	1.50	0.76	0.30	100	0.09	0.02	0.04	-2.31	-0.06	-2.42
				250	-0.17	-0.01	0.01	-2.36	-0.06	-2.41

				400	-0.18	-0.04	-0.03	-2.40	-0.04	-2.39
			0.50	100	0.06	-0.00	0.03	-3.95	-0.07	-4.06
				250	-0.16	-0.03	-0.01	-4.00	-0.06	-4.05
				400	-0.20	-0.06	-0.04	-4.04	-0.02	-4.02
							Var$\times N$			
0.00	1.00	0.50	0.30	100	39.37	9.85	9.68	8.26	9.50	7.70
				250	14.00	4.01	3.88	3.42	3.74	3.15
				400	7.59	2.53	2.43	2.16	2.38	2.02
			0.50	100	40.85	9.78	9.00	8.07	8.62	6.83
				250	14.74	4.02	3.55	3.31	3.41	2.65
				400	7.92	2.42	2.17	2.03	2.16	1.66
0.00	1.50	0.50	0.30	100	38.50	9.78	9.72	8.85	9.56	8.16
				250	13.78	3.98	3.88	3.65	3.79	3.29
				400	7.43	2.42	2.36	2.18	2.38	2.10
			0.50	100	39.70	9.60	9.15	8.60	8.72	7.28
				250	14.36	4.02	3.72	3.58	3.52	2.88
				400	7.70	2.40	2.24	2.16	2.25	1.82
0.60	1.00	0.664	0.30	100	58.92	8.21	8.03	7.62	8.00	7.21
				250	20.39	3.35	3.23	3.16	3.14	2.94
				400	10.68	2.13	2.04	2.01	2.00	1.88
			0.50	100	60.47	7.95	7.27	7.80	7.05	6.62
				250	21.17	3.28	2.91	3.21	2.79	2.56
				400	11.10	2.00	1.79	1.97	1.78	1.62
0.76	1.50	0.664	0.30	100	57.80	8.22	8.15	8.01	8.09	7.39
				250	20.14	3.38	3.30	3.32	3.23	3.01
				400	10.60	2.08	2.03	2.01	2.03	1.92
			0.50	100	59.45	8.03	7.64	8.08	7.35	6.84
				250	20.77	3.37	3.13	3.36	2.96	2.70
				400	10.91	2.05	1.92	2.05	1.89	1.71
1.00	1.00	0.76	0.30	100	71.53	6.18	6.04	6.13	6.06	5.85
				250	24.35	2.53	2.44	2.54	2.38	2.39
				400	12.54	1.61	1.55	1.63	1.52	1.53
			0.50	100	72.95	5.86	5.38	6.43	5.32	5.58
				250	25.12	2.44	2.17	2.66	2.08	2.14
				400	12.96	1.50	1.34	1.64	1.33	1.35
1.275	1.50	0.76	0.30	100	70.40	6.23	6.17	6.38	6.24	5.97
				250	24.16	2.58	2.52	2.65	2.47	2.42
				400	12.51	1.61	1.57	1.62	1.55	1.54
			0.50	100	72.05	6.03	5.73	6.52	5.69	5.70
				250	24.74	2.55	2.38	2.74	2.25	2.22
				400	12.82	1.57	1.48	1.69	1.45	1.41
							Coverage of 95% CI			
0.00	1.00	0.50	0.30	100	94.3	94.6	94.3	83.4	94.1	84.2
				250	94.8	94.3	94.2	67.9	94.7	67.5
				400	95.2	94.6	94.5	53.3	94.4	51.6
			0.50	100	94.8	95.0	94.2	63.9	94.4	63.2
				250	95.2	94.5	94.6	28.2	95.0	24.4
				400	95.4	95.1	94.7	10.2	95.0	8.0
0.00	1.50	0.50	0.30	100	94.5	94.1	93.8	88.0	94.1	88.5
				250	94.9	94.4	93.9	78.0	94.5	78.7
				400	94.4	94.6	94.5	69.3	94.4	68.7
			0.50	100	94.8	94.7	94.3	76.3	94.8	77.4

				250	94.8	94.4	94.5	50.3	95.1	49.7
				400	94.8	94.8	94.5	31.0	94.9	28.8
0.60	1.00	0.664	0.30	100	94.8	94.7	93.9	86.2	94.0	86.6
				250	94.9	94.5	94.1	70.6	94.7	70.6
				400	95.1	94.3	94.2	56.7	94.5	55.3
			0.50	100	95.1	95.2	94.2	68.6	94.7	68.6
				250	95.3	94.5	94.8	33.4	95.1	30.0
				400	95.6	94.9	94.8	13.5	95.1	10.5
0.765	1.50	0.664	0.30	100	94.6	94.5	94.1	90.1	94.2	90.2
				250	95.2	94.5	94.2	79.9	94.3	81.2
				400	94.9	94.4	94.5	71.5	94.4	71.1
			0.50	100	95.0	94.7	94.1	79.8	94.8	81.3
				250	95.1	94.3	94.3	55.4	94.9	54.8
				400	95.3	94.6	94.6	35.0	94.8	33.5
1.00	1.00	0.76	0.30	100	95.0	94.7	93.8	88.1	93.7	88.6
				250	95.1	94.4	94.1	73.8	94.9	73.3
				400	95.0	94.1	94.3	60.6	94.7	58.8
			0.50	100	95.2	95.1	94.1	73.3	94.3	73.3
				250	95.2	94.4	94.9	38.4	95.0	36.2
				400	95.6	94.7	94.9	18.0	95.0	14.4
1.275	1.50	0.76	0.30	100	94.8	94.4	94.2	91.5	94.1	91.5
				250	95.2	94.5	94.6	81.7	94.4	83.1
				400	95.1	94.3	94.2	74.4	94.4	74.5
			0.50	100	95.0	95.0	94.5	82.9	94.3	84.0
				250	95.0	94.1	94.1	59.8	95.0	60.0
				400	95.4	94.6	94.4	39.8	94.7	39.3
					Power for testing $H_0 : AUC_x = 0.5$					
0.00	1.00	0.50	0.30	100	5.7	5.4	5.7	16.6	5.9	15.8
				250	5.2	5.7	5.8	32.1	5.3	32.5
				400	4.8	5.4	5.5	46.7	5.6	48.4
			0.50	100	5.2	5.0	5.8	36.1	5.6	36.8
				250	4.8	5.5	5.4	71.8	5.0	75.6
				400	4.6	4.9	5.3	89.8	5.0	92.0
0.00	1.50	0.50	0.30	100	5.5	5.9	6.2	12.0	5.9	11.5
				250	5.1	5.6	6.1	22.0	5.5	21.3
				400	5.6	5.4	5.5	30.7	5.6	31.3
			0.50	100	5.2	5.3	5.7	23.7	5.2	22.6
				250	5.2	5.6	5.5	49.7	4.9	50.3
				400	5.2	5.2	5.5	69.0	5.1	71.2
0.60	1.00	0.664	0.30	100	32.1	96.2	96.7	89.2	97.1	90.3
				250	70.4	100	100	99.9	100	99.9
				400	93.8	100	100	100	100	100
			0.50	100	31.1	97.0	98.3	74.1	98.5	74.5
				250	68.6	100	100	98.20	100	99.10
				400	91.9	100	100	100	100	100
0.765	1.50	0.664	0.30	100	31.9	97.1	97.4	92.0	97.7	92.4
				250	71.0	100	100	100	100	100
				400	93.5	100	100	100	100	100
			0.50	100	31.5	97.2	98.0	82.3	98.4	83.8
				250	70.1	100	100	99.3	100	99.7
				400	92.5	100	100	100	100	100
1.00	1.00	0.76	0.30	100	56.1	100	100	100	100	100

				250	95.6	100	100	100	100	100
				400	99.9	100	100	100	100	100
			0.50	100	54.7	100	100	100	100	100
				250	94.9	100	100	100	100	100
				400	99.9	100	100	100	100	100
1.275	1.50	0.76	0.30	100	56.7	100	100	100	100	100
				250	96.0	100	100	100	100	100
				400	99.9	100	100	100	100	100
			0.50	100	55.4	100	100	100	100	100
				250	95.4	100	100	100	100	100
				400	99.9	100	100	100	100	100

*finite-population stratified sampling

Table 2: Performance of various estimators of $\Delta AUC = AUC_y - AUC_x$ when the two markers have same variability and same correlation with covariate W^* conditional on D, for the bi-normal model described in Section 3. Disease prevalence is 0.1. Marker X and Y is each standard normal among controls. Here we have $\mu_{Dy} = 1$, $\sigma_{Dx} = \sigma_{Dy} = 1$, $AUC_y = 0.76$, $\rho_{xw^*} = \rho_{yw^*} = 0.5$. n_D and $n_{\bar{D}}$ indicate expected number of cases and controls sampled in phase two for Bernoulli sampling and exact number of cases and controls sampled for finite-population stratified sampling. Results are based on 5,000 Monte-Carlo Simulations.

					Bernoulli Sampling				*FPS sampling	
μ_{Dx}	AUC_x	ΔAUC	ρ_{xy}	$n_D = n_{\bar{D}}$	$\Delta\widetilde{AUC}(p)$	$\Delta\widehat{AUC}(p)$	$\Delta\widehat{AUC}(\hat{p}) = \Delta\widetilde{AUC}(\hat{p})$	$\Delta\widehat{AUC}^{em}$	$\Delta\widehat{AUC}(\hat{p})$	$\Delta\widehat{AUC}^{em}$
							Bias×100			
1.00	0.76	0.00	0.00	100	0.09	0.10	0.12	0.11	0.12	0.13
				250	0.02	0.03	0.03	0.02	0.07	0.09
				400	-0.00	-0.00	-0.00	-0.00	0.05	0.07
			0.50	100	0.07	0.07	0.08	0.06	-0.01	0.01
				250	0.02	0.02	0.03	0.03	-0.02	-0.02
				400	-0.01	-0.01	-0.01	0.01	-0.03	-0.03
0.60	0.664	0.096	0.00	100	0.10	0.11	0.12	0.98	0.14	1.03
				250	0.01	0.04	0.03	0.90	0.08	0.97
				400	-0.01	0.01	0.01	0.88	0.05	0.94
			0.50	100	0.10	0.11	0.11	0.96	0.00	0.90
				250	0.02	0.04	0.04	0.92	-0.01	0.86
				400	-0.02	-0.00	-0.01	0.89	-0.03	0.85
							var×N			
1.00	0.76	0.00	0.00	100	11.48	11.47	11.66	13.31	11.94	13.63
				250	4.75	4.75	4.78	5.48	4.72	5.34
				400	2.90	2.92	2.94	3.42	2.92	3.36
			0.50	100	6.33	6.33	6.44	7.26	6.42	7.05
				250	2.48	2.49	2.51	2.88	2.62	2.91
				400	1.58	1.59	1.59	1.84	1.62	1.82
0.60	0.664	0.096	0.00	100	14.36	13.52	13.79	14.68	13.92	14.97
				250	5.82	5.56	5.59	6.02	5.50	5.86
				400	3.52	3.41	3.42	3.75	3.42	3.69
			0.50	100	8.32	7.43	7.55	7.96	7.57	7.73
				250	3.20	2.94	2.94	3.16	3.08	3.21

				400	1.98	1.85	1.85	2.00	1.91	2.01
							Coverage of 95% CI			
1.00	0.76	0.00	0.00	100	95.3	95.0	94.6	94.8	94.8	95.1
				250	94.8	94.8	94.7	94.8	94.7	95.0
				400	95.4	95.1	95.0	94.7	94.7	95.1
			0.50	100.0	95.8	94.9	94.8	94.8	94.7	95.0
				250	95.3	95.4	95.3	94.9	94.6	94.8
				400	95.3	95.3	95.2	95.0	95.0	95.0
0.60	0.664	0.096	0.00	100	95.1	95.1	94.8	94.4	94.8	94.6
				250	94.9	94.6	94.3	94.0	94.7	93.8
				400	95.4	95.0	94.9	93.3	94.8	93.1
			0.50	100	95.1	95.1	94.6	94.2	94.5	94.5
				250	95.1	95.3	95.2	93.5	94.4	93.1
				400	95.3	95.2	95.1	92.4	94.6	92.8
							Power for testing $H_0 : AUC_x = 0.5$			
1.00	0.76	0.00	0.00	100	4.7	5.0	5.4	5.1	5.2	4.8
				250	5.2	5.2	5.3	5.2	5.3	5.0
				400	4.6	4.9	5.0	5.2	5.3	4.9
			0.50	100	4.2	5.1	5.2	5.1	5.3	4.9
				250	4.7	4.6	4.7	5.1	5.4	5.1
				400	4.7	4.7	4.8	5.0	5.0	5.0
0.60	0.664	0.096	0.00	100	40.6	45.7	46.7	48.9	46.7	49.4
				250	81.2	82.4	82.5	85.6	83.3	86.5
				400	95.0	95.4	95.4	96.6	95.9	96.9
			0.50	100	65.1	70.7	71.4	75.1	70.7	75.5
				250	97.2	97.7	97.6	98.8	97.6	98.6
				400	99.9	100	100	100	99.9	100

*finite-population stratified sampling

Table 3: Performance of different estimators of $\Delta AUC = AUC_y - AUC_x$ when the two markers have same variability but different correlation with covariate W^* conditional on D, for the bi-normal marker model described in Section 3. Disease prevalence is 0.1. Marker X and Y is each standard normal among controls. Here we have $\mu_{Dy} = 1$, $\sigma_{Dx} = \sigma_{Dy} = 1$, $AUC_y = 0.76$, $\rho_{yw^*} = 0.5$, $\rho_{xy} = 0.5$. n_D and $n_{\bar{D}}$ indicate expected number of cases and controls sampled in phase two for Bernoulli sampling and exact number of cases and controls sampled for finite-population stratified sampling. Results are based on 5,000 Monte-Carlo Simulations.

					Bernoulli Sampling				*FPS sampling	
μ_{Dx}	AUC_x	ΔAUC	ρ_{xw^*}	$n_D = n_{\bar{D}}$	$\Delta\widetilde{AUC}(p)$	$\Delta\widehat{AUC}(p)$	$\Delta\widehat{AUC}(\hat{p}) = \Delta\widehat{AUC}(\hat{p})$	$\Delta\widehat{AUC}^{em}$	$\Delta\widehat{AUC}(\hat{p})$	$\Delta\widehat{AUC}^{em}$
							Bias×100			
1.00	0.76	0.00	0.40	100	0.07	0.06	0.07	-0.99	0.01	-1.02
				250	-0.01	-0.01	-0.01	-1.07	0.03	-1.04
				400	-0.04	-0.04	-0.04	-1.09	0.01	-1.05
			0.30	100	0.03	0.01	0.03	-2.04	-0.03	-2.13
				250	-0.01	-0.02	-0.01	-2.10	0.01	-2.10
				400	-0.01	-0.02	-0.01	-2.10	0.02	-2.09
			0.20	100	0.10	0.07	0.11	-2.98	0.03	-3.09
				250	0.04	0.02	0.04	-3.08	0.02	-3.09

				400	0.03	0.02	0.03	-3.09	0.01	-3.09
			0.10	100	0.10	0.05	0.11	-4.03	0.02	-4.10
				250	0.02	-0.00	0.02	-4.10	0.04	-4.09
				400	0.02	0.01	0.03	-4.11	0.01	-4.12
0.60	0.664	0.096	0.40	100	0.09	0.08	0.09	-0.29	0.03	-0.30
				250	-0.00	0.01	0.01	-0.36	0.04	-0.33
				400	-0.04	-0.02	-0.02	-0.39	0.02	-0.35
			0.30	100	0.03	0.01	0.03	-1.52	-0.03	-1.62
				250	-0.04	-0.03	-0.02	-1.59	0.01	-1.58
				400	-0.04	-0.03	-0.02	-1.59	0.01	-1.57
			0.20	100	0.12	0.09	0.12	-2.62	0.03	-2.76
				250	0.02	0.02	0.04	-2.74	0.01	-2.75
				400	-0.00	0.01	0.02	-2.76	0.01	-2.75
			0.10	100	0.10	0.06	0.11	-3.85	0.03	-3.92
				250	-0.01	-0.01	0.01	-3.93	0.04	-3.92
				400	-0.01	0.00	0.02	-3.94	0.01	-3.95
					Var×N					
1.00	0.76	0.00	0.40	100	6.56	6.55	6.63	7.18	6.47	6.98
				250	2.51	2.51	2.51	2.78	2.53	2.74
				400	1.60	1.61	1.61	1.77	1.63	1.78
			0.30	100	6.59	6.60	6.62	6.98	6.63	6.81
				250	2.60	2.61	2.57	2.75	2.66	2.69
				400	1.63	1.64	1.62	1.75	1.62	1.67
			0.20	100	6.81	6.77	6.64	6.90	6.44	6.34
				250	2.64	2.63	2.56	2.65	2.64	2.57
				400	1.70	1.70	1.66	1.72	1.62	1.59
			0.10	100	7.04	7.04	6.75	7.00	6.40	5.88
				250	2.69	2.71	2.56	2.71	2.51	2.33
				400	1.66	1.67	1.57	1.66	1.61	1.46
0.60	0.664	0.096	0.40	100	8.77	7.69	7.85	7.92	7.61	7.71
				250	3.31	2.95	2.97	3.08	2.97	3.04
				400	2.05	1.88	1.88	1.95	1.92	1.97
			0.30	100	8.95	7.80	7.92	7.78	7.81	7.66
				250	3.48	3.06	3.05	3.06	3.14	3.03
				400	2.15	1.94	1.93	1.96	1.91	1.88
			0.20	100	9.17	7.87	7.82	7.67	7.70	7.23
				250	3.61	3.11	3.06	2.98	3.11	2.90
				400	2.23	2.00	1.96	1.92	1.91	1.80
			0.10	100	9.51	8.16	7.97	7.81	7.57	6.68
				250	3.68	3.15	3.02	3.02	2.95	2.63
				400	2.20	1.94	1.86	1.85	1.90	1.67
					Coverage of 95% CI					
1.00	0.76	0.00	0.40	100	95.4	95.1	94.6	94.1	95.0	94.2
				250	95.3	95.1	95.0	92.0	95.0	93.2
				400	95.3	95.2	95.1	90.5	94.4	91.2
			0.30	100	95.5	94.9	94.5	91.4	94.7	90.7
				250	95.1	94.7	94.7	85.7	94.8	85.6
				400	94.9	94.7	94.7	79.1	94.8	79.1
			0.20	100	95.0	94.4	94.2	86.7	94.7	86.9
				250	95.2	94.8	95.0	73.6	94.8	74.1
				400	94.6	94.5	94.2	60.2	94.9	60.7
			0.10	100	94.8	94.6	94.3	79.4	94.3	81.1

				250	95.1	95.0	95.1	57.7	94.8	57.4
				400	95.3	95.0	95.0	38.1	95.1	37.2
0.60	0.664	0.096	0.40	100	94.9	94.7	94.5	95.2	94.7	95.0
				250	95.0	95.3	95.2	94.8	95.0	94.9
				400	95.1	95.4	95.3	94.5	94.5	94.4
			0.30	100	95.0	94.8	94.5	92.6	94.7	92.6
				250	94.6	94.8	95.0	90.1	94.5	89.8
				400	94.9	94.7	94.7	86.7	94.6	87.4
			0.20	100	94.5	94.4	94.1	89.1	94.6	88.9
				250	94.9	95.0	95.1	80.0	94.5	79.9
				400	94.4	94.0	94.2	70.3	94.7	70.6
			0.10	100	94.7	94.8	94.5	82.2	94.2	83.5
				250	94.4	94.8	94.9	63.3	94.8	64.2
				400	95.3	94.9	95.0	46.1	94.8	45.7
					Power for testing $H_0 : AUC_x = AUC_y$					
1.00	0.76	0.00	0.40	100	4.6	4.9	5.4	5.6	5.0	7.9
				250	4.7	4.9	5.0	7.9	5.0	6.7
				400	4.7	4.8	4.9	9.5	5.6	8.8
			0.30	100	4.5	5.1	5.5	8.5	5.3	14.2
				250	4.9	5.3	5.3	14.2	5.2	14.3
				400	5.1	5.3	5.3	20.9	5.2	20.7
			0.20	100	5.0	5.6	5.8	13.1	5.0	26.3
				250	4.8	5.2	5.0	26.3	5.2	25.7
				400	5.4	5.5	5.8	39.6	5.1	39.2
			0.10	100	5.2	5.4	5.7	20.3	4.9	42.0
				250	4.9	5.0	4.9	42.0	5.2	42.5
				400	4.7	5.0	5.0	61.7	4.9	62.6
0.60	0.664	0.096	0.40	100	63.0	69.7	70.2	64.7	97.8	95.8
				250	97.3	97.8	97.8	95.8	97.9	96.3
				400	99.8	99.8	99.8	99.6	99.9	99.6
			0.30	100	60.4	68.1	69.4	54.2	97.5	90.1
				250	96.4	97.3	97.5	90.1	97.5	90.1
				400	99.7	99.8	99.8	98.3	99.9	98.6
			0.20	100	60.1	68.8	70.0	43.1	97.7	79.9
				250	96.1	97.3	97.7	79.9	97.7	80.3
				400	99.8	99.9	99.9	94.2	99.8	94.8
			0.10	100	59.1	68.1	70.6	31.7	97.8	64.4
				250	95.4	96.9	97.8	64.4	98.1	65.8
				400	99.7	99.8	99.9	83.8	99.9	84.8

*finite-population stratified sampling

Table 4: Performance of different estimators of $\Delta AUC = AUC_y - AUC_x$ when the two markers have different variability among cases, for the binormal model described in Section 3. Disease prevalence is 0.2. Marker X and Y is each standard normal among controls. Here we have $\mu_{Dy} = 1$, $\sigma_{Dy} = 1$, $AUC_y = 0.76$, $\rho_{yw^*} = 0.5$, $\rho_{xy} = 0.5$. n_D and $n_{\bar{D}}$ indicate expected number of cases and controls sampled in phase two for Bernoulli sampling and exact number of cases and controls sampled for finite-population stratified sampling. Results are based on 5,000 Monte-Carlo Simulations.

	Bernoulli Sampling	*FPS sampling

μ_{Dx}	σ_{Dx}	AUC_x	ΔAUC	ρ_{xw^*}	$n_D = n_{\bar{D}}$	$\Delta\widetilde{AUC}(p)$	$\Delta\widehat{AUC}(p)$	$\Delta\widehat{AUC}(\hat{p})$ $= \Delta\widetilde{AUC}(\hat{p})$	$\Delta\widehat{AUC}^{em}$	$\Delta\widehat{AUC}(\hat{p})$	$\Delta\widehat{AUC}^{em}$
								Bias×100			
1.275	1.50	0.76	0.00	0.50	100	0.04	0.02	0.04	-1.11	0.04	-1.08
					250	0.01	0.00	0.01	-1.13	-0.01	-1.15
					400	0.00	-0.00	0.01	-1.12	-0.02	-1.16
				0.30	100	0.05	0.01	0.04	-2.69	-0.03	-2.79
					250	0.01	-0.01	0.01	-2.75	-0.01	-2.77
					400	-0.01	-0.02	-0.01	-2.76	-0.00	-2.76
1.581	2.00	0.76	0.00	0.50	100	-0.06	-0.10	-0.07	-2.02	0.10	-1.83
					250	-0.03	-0.03	-0.02	-1.95	0.02	-1.91
					400	0.02	0.01	0.02	-1.90	-0.00	-1.93
				0.30	100	0.02	-0.02	0.01	-3.19	-0.04	-3.27
					250	-0.00	-0.02	-0.00	-3.22	-0.01	-3.23
					400	-0.00	-0.01	-0.00	-3.21	-0.02	-3.24
0.765	1.50	0.664	0.10	0.50	100	0.05	0.04	0.05	-0.43	0.05	-0.39
					250	-0.01	0.01	0.01	-0.45	0.00	-0.46
					400	-0.01	0.01	0.01	-0.44	-0.01	-0.47
				0.30	100	0.05	0.01	0.04	-2.29	-0.02	-2.38
					250	-0.02	-0.01	-0.00	-2.35	0.00	-2.35
					400	-0.03	-0.02	-0.01	-2.35	0.01	-2.35
0.949	2.00	0.664	0.096	0.50	100	-0.04	-0.07	-0.04	-1.46	0.11	-1.27
					250	-0.04	-0.03	-0.02	-1.40	0.03	-1.36
					400	0.00	0.02	0.03	-1.35	0.00	-1.37
				0.30	100	0.02	-0.02	0.01	-2.86	-0.03	-2.93
					250	-0.03	-0.02	-0.01	-2.90	-0.00	-2.90
					400	-0.02	-0.01	-0.00	-2.89	-0.01	-2.90
								Var×N			
1.275	1.50	0.76	0.00	0.50	100	6.79	6.79	6.84	7.62	6.57	7.29
					250	2.69	2.69	2.69	3.05	2.71	3.01
					400	1.66	1.67	1.67	1.90	1.70	1.92
				0.30	100	7.08	7.02	6.92	7.55	6.85	7.26
					250	2.75	2.74	2.67	2.94	2.68	2.81
					400	1.71	1.72	1.67	1.84	1.67	1.77
1.581	2.00	0.76	0.00	0.50	100	7.27	7.29	7.27	7.98	6.91	7.82
					250	2.90	2.91	2.89	3.21	2.82	3.17
					400	1.81	1.82	1.81	2.00	1.77	2.01
				0.30	100	7.56	7.49	7.32	8.07	7.20	7.73
					250	2.97	2.97	2.87	3.17	2.85	3.03
					400	1.85	1.86	1.79	1.98	1.79	1.92
0.765	1.50	0.664	0.096	0.50	100	8.97	7.84	7.93	8.39	7.68	8.05
					250	3.45	3.13	3.14	3.37	3.17	3.35
					400	2.07	1.91	1.91	2.07	1.98	2.12
				0.30	100	9.45	8.09	8.07	8.36	7.98	8.13
					250	3.64	3.19	3.14	3.27	3.12	3.15
					400	2.21	1.98	1.95	2.04	1.95	1.98
0.949	2.00	0.664	0.096	0.50	100	9.69	8.44	8.47	8.92	8.03	8.71
					250	3.68	3.34	3.34	3.57	3.28	3.56
					400	2.22	2.05	2.05	2.18	2.06	2.24
				0.30	100	10.00	8.57	8.44	8.96	8.26	8.63
					250	3.89	3.41	3.33	3.52	3.28	3.39
					400	2.36	2.12	2.07	2.18	2.06	2.14

						Coverage of 95% CI					
1.275	1.50	0.76	0.00	0.50	100	95.4	95.2	94.8	93.6	95.0	94.1
					250	94.9	94.7	94.6	91.9	94.6	92.2
					400	94.8	94.9	94.6	90.5	94.7	90.3
				0.30	100	94.8	94.5	94.2	88.0	94.3	88.1
					250	94.7	94.7	94.8	78.8	94.8	78.5
					400	94.5	94.5	94.6	68.3	94.9	68.4
1.581	2.00	0.76	0.00	0.50	100	95.6	94.7	94.7	91.4	95.3	92.3
					250	94.8	94.6	94.8	87.8	94.6	86.9
					400	94.7	94.8	94.7	83.5	94.4	83.5
				0.30	100	94.9	94.6	94.3	86.4	94.6	85.6
					250	94.5	94.7	94.4	73.6	94.6	73.7
					400	94.5	94.4	94.5	61.8	94.6	60.9
0.765	1.50	0.664	0.096	0.50	100	94.5	94.9	94.7	94.7	94.7	95.0
					250	94.6	94.7	94.8	94.0	94.3	94.2
					400	94.9	95.0	94.9	93.9	94.4	93.6
				0.30	100	94.5	94.6	94.3	90.3	94.4	89.7
					250	94.6	94.5	94.6	84.2	94.9	84.1
					400	94.6	94.5	94.5	77.4	94.8	77.0
0.949	2.00	0.664	0.096	0.50	100	94.6	94.4	94.3	93.0	95.1	93.7
					250	94.9	94.8	94.5	91.2	94.8	91.0
					400	95.2	94.9	94.7	89.5	94.5	89.2
				0.30	100	94.8	95.1	94.6	88.7	94.6	87.9
					250	94.6	94.7	94.3	79.3	94.5	79.3
					400	94.7	94.6	94.7	70.2	94.6	70.0
						Power for testing $H_0 : AUC_x = AUC_y$					
1.275	1.50	0.76	0.00	0.50	100	4.6	4.8	5.2	6.3	5.0	5.8
					250	5.1	5.3	5.4	8.0	5.4	7.7
					400	5.2	5.1	5.4	9.5	5.3	9.6
				0.30	100	5.2	5.5	5.8	11.8	5.7	11.8
					250	5.3	5.3	5.2	21.1	5.2	21.3
					400	5.5	5.5	5.4	31.6	5.1	31.5
1.581	2.00	0.76	0.00	0.50	100	4.4	5.3	5.3	8.5	4.70	7.5
					250	5.2	5.4	5.20	12.1	5.4	13.0
					400	5.3	5.2	5.30	16.4	5.6	16.5
				0.30	100	5.1	5.4	5.7	13.4	5.4	14.2
					250	5.5	5.3	5.6	26.3	5.4	26.2
					400	5.5	5.6	5.5	38.1	5.4	39.0
0.765	1.50	0.66	0.096	0.50	100	61.1	68.1	68.9	61.1	69.9	62.3
					250	96.3	96.9	97.0	94.8	97.1	94.6
					400	99.8	99.9	99.9	99.6	99.8	99.3
				0.30	100	59.5	67.5	68.6	44.4	69.6	44.4
					250	95.8	96.8	97.1	81.9	97.6	82.0
					400	99.7	99.8	99.9	95.2	99.9	95.4
0.949	2.00	0.66	0.096	0.50	100	57.5	65.1	66.1	49.1	67.9	50.9
					250	95.2	96.2	96.2	87.5	96.6	87.6
					400	99.6	99.7	99.8	97.6	99.6	97.6
				0.30	100	56.4	64.7	66.4	37.4	66.9	37.2
					250	94.8	95.8	96.3	72.2	96.9	73.0
					400	99.6	99.7	99.8	89.9	99.9	89.9

*finite-population stratified sampling

Table 5: Performance of different AUC_x estimators for the bi-gamma marker model described in Appendix H1. Disease prevalence is 0.1. Biomarker X follows gamma distribution with shape parameter κ_x conditional on D, scale parameter 1 among controls, and scale parameter σ_{Dx} among cases. n_D and $n_{\bar{D}}$ indicate expected number of cases and controls sampled in phase two for Bernoulli sampling and exact number of cases and controls sampled for finite-population stratified sampling. Results are based on 5,000 Monte-Carlo Simulations.

					Bernoulli Sampling				*FPS sampling	
κ_x	σ_{Dx}	AUC_x	ρ_{xw^*}	$n_D = n_{\bar{D}}$	$\widetilde{AUC}_x(p)$	$\widehat{AUC}_x(p)$	$\widehat{AUC}_x(\hat{p}) = \widetilde{AUC}_x(\hat{p})$	$\widehat{AUC}_x^{em}$	$\widehat{AUC}_x(\hat{p})$	$\widehat{AUC}_x^{em}$
					Bias×100					
1.00	1.00	0.50	0.30	100	-0.06	-0.13	-0.07	-4.35	-0.06	-4.32
				250	-0.16	-0.08	-0.06	-4.33	-0.04	-4.33
				400	-0.17	-0.08	-0.06	-4.34	-0.01	-4.30
			0.50	100	0.02	-0.08	0.01	-7.24	-0.05	-7.29
				250	-0.13	-0.07	-0.03	-7.28	-0.03	-7.27
				400	-0.13	-0.05	-0.02	-7.27	0.01	-7.25
0.33	1.00	0.50	0.30	100	-0.04	-0.11	-0.05	-5.03	-0.10	-5.07
				250	-0.17	-0.09	-0.06	-5.03	-0.06	-5.03
				400	-0.15	-0.07	-0.04	-5.03	-0.04	-5.02
			0.50	100	0.00	-0.12	-0.01	-8.50	-0.06	-8.55
				250	-0.14	-0.08	-0.03	-8.51	-0.07	-8.55
				400	-0.17	-0.09	-0.06	-8.53	-0.03	-8.51
1.00	1.50	0.60	0.30	100	-0.06	-0.13	-0.07	-3.98	-0.07	-3.94
				250	-0.18	-0.08	-0.05	-3.94	-0.05	-3.94
				400	-0.18	-0.07	-0.06	-3.95	-0.01	-3.92
			0.50	100	0.02	-0.07	0.01	-6.60	-0.05	-6.64
				250	-0.15	-0.06	-0.02	-6.64	-0.03	-6.62
				400	-0.15	-0.04	-0.01	-6.63	0.00	-6.60
0.33	2.36	0.60	0.30	100	-0.04	-0.10	-0.05	-4.38	-0.10	-4.42
				250	-0.18	-0.08	-0.05	-4.37	-0.06	-4.38
				400	-0.17	-0.06	-0.04	-4.37	-0.03	-4.36
			0.50	100	-0.01	-0.12	-0.02	-7.38	-0.07	-7.42
				250	-0.16	-0.08	-0.03	-7.39	-0.07	-7.42
				400	-0.18	-0.08	-0.05	-7.40	-0.03	-7.38
1.00	2.34	0.70	0.30	100	-0.04	-0.11	-0.06	-3.33	-0.07	-3.29
				250	-0.20	-0.07	-0.05	-3.29	-0.05	-3.29
				400	-0.19	-0.06	-0.05	-3.29	-0.01	-3.27
			0.50	100	0.01	-0.06	0.00	-5.50	-0.06	-5.54
				250	-0.17	-0.06	-0.03	-5.54	-0.03	-5.52
				400	-0.17	-0.04	-0.02	-5.53	-0.00	-5.50
0.33	6.20	0.70	0.30	100	-0.03	-0.09	-0.04	-3.46	-0.10	-3.51
				250	-0.19	-0.06	-0.04	-3.45	-0.05	-3.46
				400	-0.18	-0.05	-0.03	-3.45	-0.03	-3.44
			0.50	100	-0.01	-0.10	-0.02	-5.82	-0.08	-5.85
				250	-0.18	-0.06	-0.03	-5.82	-0.06	-5.84
				400	-0.20	-0.07	-0.04	-5.83	-0.02	-5.81
					Var×N					
1.00	1.00	0.50	0.30	100	39.37	9.74	9.50	8.33	9.34	7.46
				250	14.04	3.91	3.73	3.27	3.74	3.03

				400	7.50	2.44	2.34	2.04	2.44	2.00
			0.50	100	41.06	9.73	8.73	8.07	8.67	6.61
				250	15.03	3.95	3.46	3.29	3.41	2.61
				400	8.05	2.46	2.18	2.04	2.14	1.64
0.33	1.00	0.50	0.30	100	40.31	10.21	9.88	8.66	9.19	7.44
				250	14.46	4.10	3.87	3.49	3.74	3.01
				400	7.62	2.41	2.27	2.02	2.35	1.89
			0.50	100	42.19	10.01	8.31	8.25	8.03	5.93
				250	15.27	3.96	3.23	3.31	3.23	2.32
				400	8.23	2.40	1.97	1.98	2.04	1.46
1.00	1.50	0.60	0.30	100	50.69	9.00	8.79	8.38	8.60	7.45
				250	17.75	3.60	3.45	3.27	3.43	3.01
				400	9.28	2.23	2.15	2.03	2.26	2.00
			0.50	100	52.27	8.86	8.03	8.31	7.98	6.82
				250	18.74	3.59	3.20	3.39	3.16	2.70
				400	9.88	2.23	2.00	2.09	1.99	1.70
0.33	2.36	0.60	0.30	100	51.26	9.30	9.06	8.69	8.39	7.45
				250	18.06	3.74	3.56	3.52	3.44	3.04
				400	9.37	2.18	2.08	2.03	2.18	1.92
			0.50	100	53.08	9.06	7.80	8.64	7.57	6.19
				250	18.89	3.63	3.10	3.50	3.05	2.44
				400	10.02	2.19	1.87	2.09	1.94	1.56
1.00	2.34	0.70	0.30	100	63.06	7.49	7.32	7.47	7.10	6.63
				250	21.72	2.98	2.87	2.91	2.82	2.67
				400	11.15	1.84	1.78	1.79	1.86	1.77
			0.50	100	64.44	7.28	6.71	7.59	6.63	6.28
				250	22.64	2.95	2.69	3.10	2.64	2.49
				400	11.75	1.83	1.67	1.90	1.67	1.57
0.33	6.20	0.70	0.30	100	63.14	7.72	7.57	7.81	7.04	6.72
				250	21.87	3.10	2.98	3.16	2.89	2.75
				400	11.23	1.82	1.75	1.83	1.84	1.75
			0.50	100	64.82	7.54	6.78	8.04	6.53	5.87
				250	22.66	3.05	2.73	3.27	2.63	2.34
				400	11.83	1.84	1.64	1.96	1.69	1.51
					Coverage of 95% CI					
1.00	1.00	0.50	0.30	100	94.5	95.1	94.6	80.90	94.0	81.9
				250	95.2	94.8	94.6	60.60	94.6	61.6
				400	95.1	94.9	94.6	43.20	94.4	43.5
			0.50	100	94.6	95.3	93.9	56.70	94.1	56.3
				250	94.6	94.7	94.4	18.70	94.8	16.2
				400	95.2	94.8	94.1	5.50	94.8	3.6
0.33	1.00	0.50	0.30	100	94.1	94.3	93.5	75.70	94.3	77.1
				250	94.7	94.4	94.5	50.10	94.6	50.2
				400	95.2	95.5	95.3	30.20	94.6	29.5
			0.50	100	94.7	94.7	94.0	44.30	94.6	43.1
				250	95.1	94.7	94.5	9.20	95.0	4.9
				400	95.4	95.3	95.1	1.10	94.7	0.5
1.00	1.50	0.60	0.30	100	94.5	95.2	94.5	83.7	94.1	85.1
				250	95.1	94.8	94.6	67.2	94.6	67.8
				400	95.3	95.1	94.8	50.6	94.4	50.9
			0.50	100	94.7	95.1	93.9	63.9	94.0	64.9
				250	94.8	94.6	94.7	26.6	94.8	24.5

				400	95.1	94.8	94.0	9.7	94.7	8.1
0.33	2.36	0.60	0.30	100	94.2	94.4	93.5	81.1	94.6	82.7
				250	95.0	94.6	94.4	61.2	94.6	61.2
				400	95.2	95.4	95.4	43.6	94.6	41.7
			0.50	100	94.7	94.7	94.1	57.1	94.5	57.0
				250	94.9	94.5	94.2	18.7	94.9	13.9
				400	95.3	95.3	95.3	4.8	94.6	2.5
1.00	2.34	0.70	0.30	100	94.8	95.0	94.5	86.6	94.1	88.3
				250	95.2	94.6	94.5	74.0	95.0	74.2
				400	95.6	95.1	95.1	59.7	94.3	60.1
			0.50	100	94.9	95.1	94.0	72.0	94.5	73.7
				250	95.0	94.2	94.5	39.3	94.5	37.7
				400	95.3	94.6	94.3	17.4	94.3	16.3
0.33	6.20	0.70	0.30	100	94.5	94.1	94.2	86.4	94.5	88.1
				250	95.2	94.5	94.2	72.0	94.3	72.5
				400	95.2	95.1	95.4	57.6	94.5	58.1
			0.50	100	94.7	95.1	94.6	70.2	94.5	72.2
				250	95.0	94.1	94.3	35.0	94.9	33.1
				400	95.4	95.1	95.0	15.1	94.8	12.4
					Power for testing $H_0 : AUC_x = 0.5$					
1.00	1.00	0.50	0.30	100	5.5	4.9	5.4	19.1	6.0	18.1
				250	4.8	5.2	5.4	39.4	5.4	38.4
				400	4.9	5.1	5.4	56.8	5.6	56.5
			0.50	100	5.4	4.7	6.1	43.3	5.9	43.7
				250	5.4	5.3	5.6	81.3	5.2	83.8
				400	4.8	5.2	5.9	94.5	5.2	96.4
0.33	1.00	0.50	0.30	100	5.9	5.7	6.5	24.3	5.7	22.9
				250	5.3	5.6	5.5	49.9	5.4	49.8
				400	4.8	4.5	4.7	69.8	5.4	70.5
			0.50	100	5.3	5.3	6.0	55.7	5.4	56.9
				250	4.9	5.3	5.5	90.8	5.0	95.1
				400	4.6	4.7	4.9	98.9	5.3	99.5
1.00	1.50	0.60	0.30	100	17.2	63.7	66.6	33.0	66.3	31.5
				250	36.7	94.7	95.5	65.7	96.3	66.7
				400	61.0	99.5	99.7	84.5	99.7	85.1
			0.50	100	17.3	64.4	70.9	13.1	70.2	10.9
				250	35.1	94.8	97.3	25.2	97.5	22.9
				400	59.0	99.6	99.9	37.8	99.8	36.8
0.33	2.36	0.60	0.30	100	17.1	63.7	66.2	28.8	66.4	27.2
				250	37.1	94.9	95.7	58.9	96.1	59.5
				400	60.4	99.6	99.7	78.7	99.6	80.0
			0.50	100	16.6	63.8	71.6	10.5	71.9	6.1
				250	35.7	95.2	97.4	17.5	97.7	12.7
				400	57.8	99.5	99.8	24.6	99.9	21.6
1.00	2.34	0.70	0.30	100	40.2	99.7	99.8	98.2	99.9	99.3
				250	83.9	100	100	100	100	100
				400	98.5	100	100	100	100	100
			0.50	100	39.6	99.7	99.9	94.9	100	96.9
				250	82.3	100	100	100	100	100
				400	97.9	100	100	100	100	100
0.33	6.20	0.70	0.30	100	40.3	99.7	99.8	97.9	99.8	98.9
				250	83.5	100	100	100	100	100

	400	98.3	100	100	100	100	100
0.50	100	39.5	99.6	99.8	92.9	99.8	96.0
	250	81.9	100	100	100	100	100
	400	97.9	100	100	100	100	100

*finite-population stratified sampling

Table 6: Performance of various estimators of $\Delta AUC = AUC_y - AUC_x$ when the two markers have same shape parameter and same correlation with covariate W^* conditional on D, for the bi-gamma model described in Appendix H1. Disease prevalence is 0.1. Marker X and Y each follows gamma distribution conditional on D with shape parameter $\kappa_x = \kappa_y$. Scale parameter is 1 among controls for each marker and is σ_{Dx} for X and σ_{Dy} for Y. Here we have $\rho_{xw^*} = \rho_{yw^*} = 0.5$, and $\rho_{xy} = 0.5$. n_D and $n_{\bar{D}}$ indicate expected number of cases and controls sampled in phase two for Bernoulli sampling and exact number of cases and controls sampled for finite-population stratified sampling. Results are based on 5,000 Monte-Carlo Simulations.

							Bernoulli Sampling				*FPS sampling	
$\kappa_x = \kappa_y$	σ_{Dy}	σ_{Dx}	AUC_y	AUC_x	ΔAUC	$n_D = n_{\bar{D}}$	$\Delta\widetilde{AUC}(p)$	$\Delta\widehat{AUC}(p)$	$\Delta\widehat{AUC}(\hat{p}) = \Delta\widetilde{AUC}(\hat{p})$	$\Delta\widehat{AUC}^{em}$	$\Delta\widehat{AUC}(\hat{p})$	$\Delta\widehat{AUC}^{em}$
							Bias×100					
1.00	1.50	1.50	0.60	0.60	0.00	100	0.08	0.08	0.07	0.05	0.08	0.11
						250	0.00	0.01	0.01	0.02	0.03	0.05
						400	-0.00	-0.00	-0.00	0.01	0.03	0.04
0.33	2.36	2.36	0.60	0.60	0.00	100	0.08	0.08	0.08	0.07	0.12	0.15
						250	0.01	0.01	0.01	0.01	0.06	0.08
						400	0.01	0.01	0.01	0.01	0.04	0.06
1.00	2.34	1.50	0.70	0.60	0.10	100	0.07	0.09	0.07	1.16	0.07	1.21
						250	-0.01	0.02	0.01	1.13	0.03	1.15
						400	-0.02	0.01	-0.00	1.11	0.03	1.14
0.33	6.20	2.36	0.70	0.60	0.10	100	0.09	0.10	0.08	1.64	0.11	1.72
						250	-0.01	0.03	0.02	1.59	0.06	1.65
						400	-0.01	0.02	0.01	1.58	0.04	1.63
							Var×N					
1.00	1.50	1.50	0.60	0.60	0.00	100	8.67	8.60	8.79	8.19	8.55	8.17
						250	3.35	3.37	3.39	3.23	3.29	3.20
						400	2.12	2.13	2.14	2.05	2.09	2.04
0.33	2.36	2.36	0.60	0.60	0.00	100	7.27	7.30	7.43	7.16	7.58	7.26
						250	2.90	2.91	2.93	2.92	2.93	2.86
						400	1.86	1.87	1.88	1.84	1.85	1.82
1.00	2.34	1.50	0.70	0.60	0.10	100	9.09	8.05	8.18	8.00	7.91	7.92
						250	3.37	3.14	3.15	3.14	3.06	3.13
						400	2.10	1.97	1.98	1.98	1.94	1.99
0.33	6.20	2.36	0.70	0.60	0.10	100	7.91	7.04	7.08	7.22	7.17	7.30
						250	3.00	2.78	2.76	2.91	2.80	2.92
						400	1.87	1.77	1.76	1.83	1.75	1.84
							Coverage of 95% CI					
1.00	1.50	1.50	0.60	0.60	0.00	100	95.5	94.9	94.6	94.9	94.6	95.3
						250	95.7	95.3	95.3	95.4	95.3	95.2
						400	95.2	94.9	94.9	95.1	95.0	95.0

0.33	2.36	2.36	0.60	0.60	0.00	100	95.6	95.2	94.9	95.1	94.6	95.0
						250	95.9	95.6	95.3	94.9	94.7	95.2
						400	95.1	95.0	94.9	94.6	94.5	95.0
1.00	2.34	1.50	0.70	0.60	0.10	100	95.0	95.0	94.3	93.6	94.6	94.2
						250	95.5	95.3	95.2	92.6	95.2	92.6
						400	95.1	94.8	94.8	90.9	95.3	90.8
0.33	6.20	2.36	0.70	0.60	0.10	100	95.1	94.9	94.7	92.8	94.5	92.6
						250	95.6	95.3	95.3	89.8	94.7	89.5
						400	95.2	94.8	95.0	86.4	94.6	86.2
							Power for testing $H_0 : AUC_x = AUC_y$					
1.00	1.50	1.50	0.60	0.60	0.00	100	4.5	5.1	5.4	5.0	5.4	4.6
						250	4.3	4.7	4.7	4.5	4.7	4.7
						400	4.8	5.1	5.1	4.9	5.0	4.9
0.33	2.36	2.36	0.60	0.60	0.00	100	4.4	4.8	5.1	4.9	5.4	4.9
						250	4.1	4.4	4.7	5.0	5.3	4.7
						400	4.9	5.0	5.1	5.3	5.5	5.0
1.00	2.34	1.50	0.70	0.60	0.10	100	65.4	71.4	72.0	79.7	73.8	80.8
						250	97.8	98.1	98.0	99.4	98.4	99.4
						400	99.9	99.9	99.8	100	99.9	100
0.33	6.20	2.36	0.70	0.60	0.10	100	71.1	76.0	76.9	85.7	77.0	85.8
						250	98.4	98.9	98.8	99.8	98.8	99.8
						400	99.9	99.9	99.9	100	99.9	100

*finite-population stratified sampling

Table 7: Performance of different estimators of $\Delta AUC = AUC_y - AUC_x$ where the two markers have same shape parameter but different correlation with covariate W^* conditional on D, for the bi-gamma marker model described in Appendix H1. Disease prevalence is 0.1. Marker X and Y each follows gamma distribution conditional on D with shape parameter $\kappa_x = \kappa_y$. Scale parameter is 1 among controls for each marker and is σ_{Dx} for X and σ_{Dy} for Y. Here we have $\rho_{yw^*} = 0.5$ and $\rho_{xy} = 0.5$. n_D and $n_{\bar{D}}$ indicate expected number of cases and controls sampled in phase two for Bernoulli sampling and exact number of cases and controls sampled for finite-population stratified sampling. Results are based on 5,000 Monte-Carlo Simulations.

								Bernoulli Sampling				*FPS sampling	
$\kappa_x = \kappa_y$	σ_{Dy}	σ_{Dx}	AUC_y	AUC_x	ΔAUC	ρ_{xw^*}	$n_D = n_{\bar{D}}$	$\Delta\widetilde{AUC}(p)$	$\Delta\widehat{AUC}(p)$	$\Delta\widehat{AUC}(\hat{p}) = \Delta\widetilde{AUC}(\hat{p})$	$\Delta\widehat{AUC}^{em}$	$\Delta\widehat{AUC}(\hat{p})$	$\Delta\widehat{AUC}^{em}$
								Bias×100					
1.00	1.50	1.50	0.60	0.60	0.00	0.30	100	0.17	0.14	0.17	-2.52	0.06	-2.66
							250	0.04	0.03	0.05	-2.67	-0.01	-2.71
							400	0.01	-0.00	0.01	-2.70	-0.02	-2.73
						0.10	100	0.07	0.01	0.07	-5.21	0.01	-5.32
							250	-0.01	-0.04	-0.01	-5.32	0.04	-5.28
							400	-0.03	-0.05	-0.02	-5.34	0.03	-5.28

0.33	2.36	2.36	0.60	0.60	0.00	0.30	100	0.13	0.11	0.14	-2.85	0.06	-2.92
							250	0.01	-0.01	0.01	-2.99	0.02	-2.99
							400	-0.03	-0.04	-0.03	-3.03	0.02	-3.00
						0.10	100	0.14	0.06	0.13	-5.80	0.04	-5.87
							250	0.02	-0.01	0.03	-5.90	-0.01	-5.92
							400	-0.02	-0.04	-0.01	-5.93	-0.01	-5.92
1.00	2.34	1.50	0.70	0.60	0.10	0.30	100	0.17	0.15	0.17	-1.41	0.04	-1.56
							250	0.03	0.04	0.05	-1.56	-0.01	-1.61
							400	-0.01	0.00	0.01	-1.60	-0.02	-1.62
						0.10	100	0.07	0.02	0.07	-4.11	0.01	-4.20
							250	-0.03	-0.03	-0.01	-4.22	0.04	-4.16
							400	-0.05	-0.04	-0.03	-4.24	0.03	-4.17
0.33	6.20	2.36	0.70	0.60	0.10	0.30	100	0.15	0.14	0.16	-1.27	0.05	-1.36
							250	0.01	0.02	0.03	-1.41	0.02	-1.42
							400	-0.04	-0.03	-0.03	-1.47	0.02	-1.42
						0.10	100	0.16	0.10	0.15	-4.21	0.03	-4.28
							250	0.01	0.01	0.03	-4.32	-0.00	-4.34
							400	-0.04	-0.04	-0.02	-4.38	0.01	-4.35
										Var $\times N$			
1.00	1.50	1.50	0.60	0.60	0.00	0.30	100	8.64	8.59	8.54	8.14	8.29	7.51
							250	3.36	3.37	3.33	3.21	3.35	3.03
							400	2.14	2.15	2.12	2.04	2.17	1.98
						0.10	100	9.12	9.03	8.32	8.16	8.00	6.71
							250	3.50	3.49	3.19	3.14	3.19	2.62
							400	2.16	2.16	1.97	1.97	2.03	1.69
0.33	2.36	2.36	0.60	0.60	0.00	0.30	100	7.56	7.55	7.44	7.33	7.38	6.87
							250	2.92	2.92	2.85	2.85	2.92	2.71
							400	1.88	1.89	1.84	1.85	1.87	1.74
						0.10	100	7.95	8.01	7.17	7.45	6.76	5.46
							250	3.10	3.10	2.74	2.90	2.66	2.13
							400	1.99	1.99	1.75	1.84	1.70	1.38
1.00	2.34	1.50	0.70	0.60	0.10	0.30	100	9.25	7.92	7.99	7.77	7.82	7.35
							250	3.53	3.11	3.11	3.08	3.19	2.99
							400	2.23	2.00	1.99	1.97	2.07	1.95
						0.10	100	9.99	8.35	7.95	7.80	7.76	6.67
							250	3.85	3.23	3.06	3.02	3.09	2.62
							400	2.33	2.02	1.90	1.89	1.97	1.69
0.33	6.20	2.36	0.70	0.60	0.10	0.30	100	8.47	7.28	7.36	7.32	7.16	6.99
							250	3.21	2.81	2.81	2.84	2.85	2.75
							400	2.00	1.79	1.78	1.84	1.83	1.78
						0.10	100	9.22	7.58	7.19	7.32	6.88	5.71
							250	3.55	2.94	2.75	2.86	2.68	2.23
							400	2.20	1.89	1.76	1.82	1.71	1.44
										Coverage of 95% CI			
1.00	1.50	1.50	0.60	0.60	0.00	0.30	100	95.1	94.9	94.3	89.8	95.4	90.3
							250	95.1	95.0	94.9	81.9	95.0	82.0
							400	94.8	94.9	94.6	71.8	94.2	71.8
						0.10	100	94.6	94.5	94.3	73.3	94.9	74.1
							250	94.8	94.5	94.8	43.6	94.6	42.9
							400	94.8	94.6	94.8	22.6	95.0	22.2
0.33	2.36	2.36	0.60	0.60	0.00	0.30	100	95.7	95.1	94.9	88.2	94.6	88.1
							250	95.3	95.0	95.4	76.1	94.8	76.6

							400	95.2	95.1	95.1	63.5	94.4	64.7
						0.10	100	95.0	94.4	94.4	65.6	95.0	66.9
							250	94.5	94.5	94.5	29.7	94.7	26.6
							400	93.8	93.7	94.0	11.6	94.6	8.6
1.00	2.34	1.50	0.70	0.60	0.10	0.30	100	95.0	95.0	94.6	93.3	95.3	93.7
							250	95.4	95.1	95.0	90.5	94.9	90.3
							400	95.2	94.9	94.8	87.1	94.4	86.7
						0.10	100	94.4	94.6	94.4	80.9	94.9	81.7
							250	94.7	94.8	94.9	59.0	94.8	61.0
							400	94.9	94.8	94.8	41.3	94.8	41.2
0.33	6.20	2.36	0.70	0.60	0.10	0.30	100	94.9	95.2	94.8	93.7	94.4	93.6
							250	95.4	95.4	95.4	90.9	94.7	90.7
							400	95.1	95.0	95.0	86.7	94.5	88.0
						0.10	100	94.5	94.7	94.4	78.7	95.0	81.2
							250	94.4	94.7	94.6	54.2	94.8	55.9
							400	94.2	94.1	94.4	35.1	94.8	34.2
								Power for testing $H_0 : AUC_x = AUC_y$					
1.00	1.50	1.50	0.60	0.60	0.00	0.30	100	4.9	5.1	5.7	10.1	4.6	9.6
							250	4.9	5.0	5.1	18.0	5.0	17.9
							400	5.2	5.1	5.4	28.1	5.8	28.0
						0.10	100	5.4	5.5	5.7	26.4	5.1	25.6
							250	5.2	5.5	5.2	56.2	5.4	56.9
							400	5.2	5.4	5.2	77.4	5.0	77.8
0.33	2.36	2.36	0.60	0.60	0.00	0.30	100	4.3	4.9	5.1	11.7	5.4	11.6
							250	4.7	5.0	4.6	23.7	5.2	23.2
							400	4.8	4.9	4.9	36.4	5.6	35.2
						0.10	100	5.0	5.6	5.6	34.1	5.0	32.7
							250	5.5	5.5	5.5	70.1	5.3	73.2
							400	6.2	6.3	6.0	88.4	5.4	91.4
1.00	2.34	1.50	0.70	0.60	0.10	0.30	100	65.3	73.2	73.6	59.3	72.2	58.1
							250	97.6	98.4	98.5	92.7	98.1	92.9
							400	99.8	99.9	99.9	98.9	99.9	99.0
						0.10	100	60.4	70.0	73.3	32.9	73.5	31.8
							250	96.4	97.7	98.3	66.0	98.6	68.0
							400	99.8	99.8	99.9	85.0	99.9	87.4
0.33	6.20	2.36	0.70	0.60	0.10	0.30	100	69.1	76.3	76.9	62.6	76.2	62.4
							250	98.2	98.7	98.8	94.8	98.8	95.0
							400	99.9	99.9	100	99.4	100	99.5
						0.10	100	64.8	74.4	78.2	33.9	78.2	30.7
							250	97.3	98.6	99.1	66.1	99.2	68.2
							400	99.9	99.9	99.9	84.4	100	87.4

*finite-population stratified sampling

Table 8: Performance of different estimators of $\Delta AUC = AUC_y - AUC_x$ when the two markers have different shape parameter conditional on D, for the bi-gamma model described in Appendix H1, with $\kappa_y = 3$, $\kappa_x = 1/3$. Disease prevalence is 0.1. Here we have $\rho_{yw^*} = 0.5$, $\rho_{xy} = 0.5$. n_D and $n_{\bar{D}}$ indicate expected number of cases and controls sampled in phase two for Bernoulli sampling and exact number of cases and controls sampled for finite-population stratified sampling. Results are based on 5,000 Monte-Carlo Simulations.

							Bernoulli Sampling				*FPS sampling	
σ_{Dy}	σ_{Dx}	AUC_y	AUC_x	ΔAUC	ρ_{xw^*}	$n_D = n_{\bar{D}}$	$\Delta\widetilde{AUC}(p)$	$\Delta\widehat{AUC}(p)$	$\Delta\widehat{AUC}(\hat{p}) = \Delta\widetilde{AUC}(\hat{p})$	$\Delta\widehat{AUC}^{em}$	$\Delta\widehat{AUC}(\hat{p})$	$\Delta\widehat{AUC}^{em}$
							Bias×100					
1.24	2.36	0.60	0.60	-0.00	0.50	100	0.04	0.05	0.05	1.04	0.02	1.01
						250	-0.03	-0.02	-0.03	0.96	0.01	1.00
						400	-0.03	-0.03	-0.03	0.96	-0.01	0.98
					0.10	100	0.12	0.06	0.11	-4.78	0.06	-4.87
						250	0.02	-0.00	0.03	-4.88	0.01	-4.91
						400	-0.00	-0.02	0.00	-4.92	0.01	-4.92
1.56	2.36	0.70	0.60	0.10	0.50	100	0.05	0.07	0.06	1.90	0.02	1.86
						250	-0.04	-0.01	-0.02	1.82	0.01	1.86
						400	-0.05	-0.03	-0.03	1.81	-0.00	1.84
					0.10	100	0.11	0.07	0.11	-3.92	0.05	-4.01
						250	0.01	0.01	0.03	-4.02	0.02	-4.04
						400	-0.02	-0.01	0.01	-4.06	0.01	-4.05
							Var×N					
1.24	2.36	0.60	0.60	-0.00	0.50	100	7.97	7.93	8.10	7.68	7.72	7.62
						250	3.09	3.10	3.11	3.02	3.05	3.00
						400	1.93	1.94	1.94	1.90	1.95	1.91
					0.10	100	7.94	7.94	7.54	7.32	7.22	6.15
						250	3.06	3.06	2.83	2.85	2.82	2.43
						400	1.95	1.95	1.79	1.82	1.80	1.56
1.56	2.36	0.70	0.60	0.10	0.50	100	8.21	7.36	7.42	7.43	7.10	7.36
						250	3.12	2.86	2.85	2.93	2.81	2.88
						400	1.90	1.79	1.77	1.84	1.79	1.84
					0.10	100	8.87	7.32	7.13	6.93	6.93	6.01
						250	3.40	2.82	2.67	2.70	2.72	2.38
						400	2.12	1.82	1.71	1.75	1.74	1.54
							Coverage of 95% CI					
1.24	2.36	0.60	0.60	-0.00	0.50	100	95.1	95.0	94.5	93.9	94.3	93.8
						250	95.1	94.7	94.7	93.4	94.9	92.7
						400	95.0	95.0	94.8	91.6	94.0	91.5
					0.10	100	95.4	95.5	94.6	74.2	95.1	75.8
						250	95.3	95.1	95.1	45.5	95.1	45.4
						400	95.0	94.9	95.1	26.3	95.0	24.3
1.56	2.36	0.70	0.60	0.10	0.50	100	95.1	94.8	94.5	92.1	94.5	92.2
						250	94.9	94.9	94.7	88.2	95.2	87.5
						400	94.9	95.0	94.9	83.5	94.1	84.2
					0.10	100	95.0	95.5	95.0	80.2	94.9	81.7
						250	95.2	95.1	95.4	58.5	94.7	59.2
						400	95.0	95.1	95.3	40.8	94.9	40.7

							Power for testing $H_0 : AUC_x = AUC_y$					
1.24	2.36	0.60	0.60	-0.00	0.50	100	4.9	5.1	5.4	5.9	5.6	6.0
						250	4.9	5.3	5.2	6.4	5.1	7.2
						400	5.0	5.0	5.1	8.2	6.0	8.3
					0.10	100	4.6	4.5	5.3	25.8	5.0	24.1
						250	4.8	4.8	4.9	54.7	4.9	54.8
						400	5.0	5.1	4.9	74.2	5.0	76.1
1.56	2.36	0.70	0.60	0.10	0.50	100	70.6	75.9	76.3	87.7	76.5	88.0
						250	98.5	98.8	98.7	99.9	99.0	99.9
						400	99.9	99.9	99.9	100	100	100
					0.10	100	65.6	75.0	77.9	37.8	77.6	36.1
						250	98.1	98.8	99.2	72.8	99.2	73.8
						400	99.9	99.9	99.9	89.5	100	90.9

*finite-population stratified sampling

References

Breslow, N. E. and Wellner, J. A. (2007). Weighted likelihood for semiparametric models and two-phase stratified samples, with application to cox regression. *Scandinavian Journal of Statistics* **34**(1), 86–102.

Dorfman, Donald D, Berbaum, Kevin S, Metz, Charles E, Lenth, Russell V, Hanley, James A and Dagga, Hatem Abu. (1997). Proper receiver operating characteristic analysis: the bigamma model. *Academic radiology* **4**(2), 138–149.

Nelsen, Roger B. (2013). *An introduction to copulas*, Volume 139. Springer Science & Business Media.

Van der Vaart, AW and Wellner, J.A. (1996). *Weak convergence and empirical processes: with applications to statistics*. Springer Verlag.

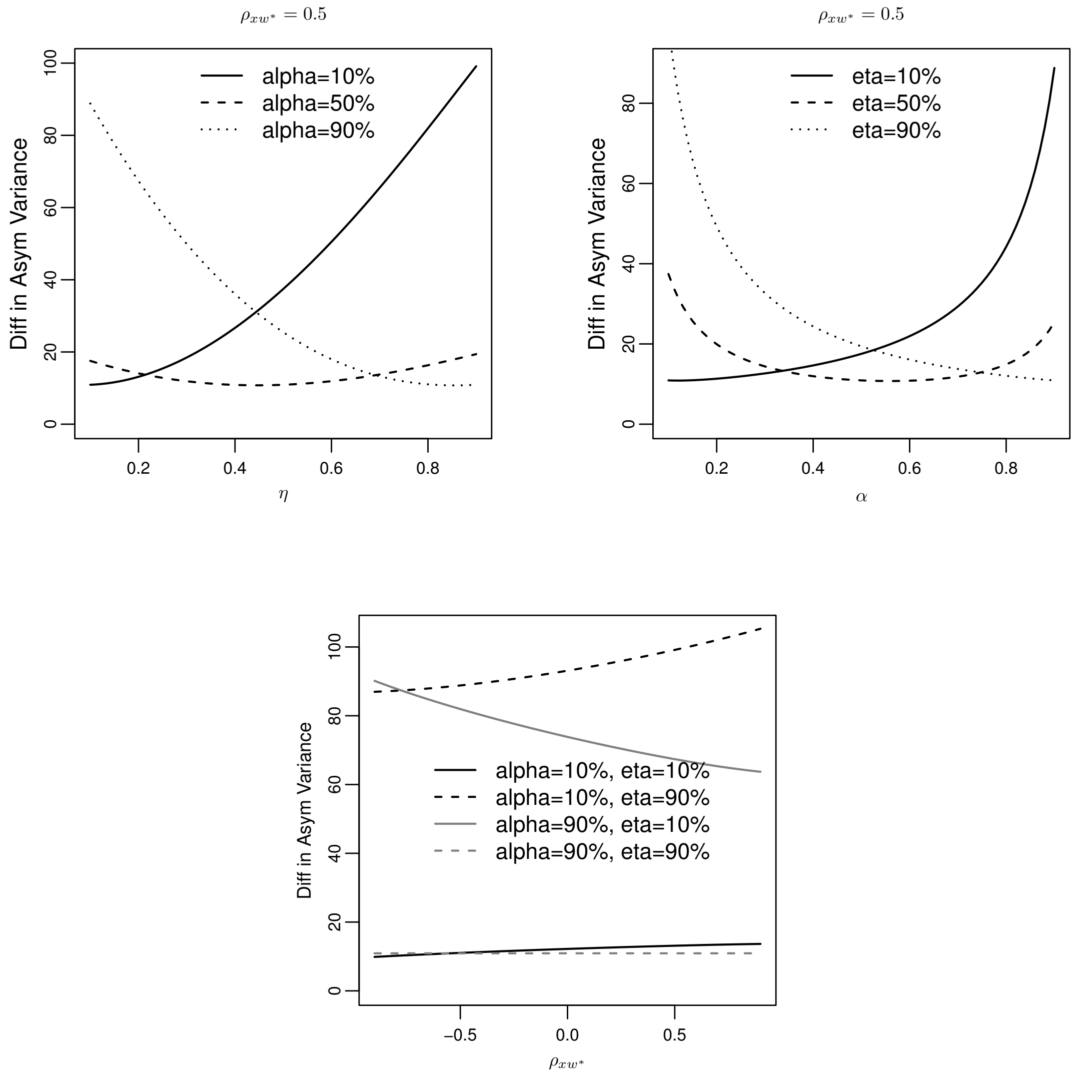

Fig. 1. Difference between asymptotic variance of $\sqrt{N}\left\{\widetilde{AUC}_x(p) - AUC_x\right\}$ and $\sqrt{N}\left\{\widehat{AUC}_x(p) - AUC_x\right\}$ for the variance component that is due to variability in controls, as a function of η, α, and $\rho_{xw^\star}$, for the setting described in Appendix E1. The value presented in Y-axis is the value in (0.6), assuming $AUC_x = 0.76$. Here $\eta = P(W = 1|D = 0)$, $\alpha = P(W = 1|D = 0, \text{Sampled in phase two})$, and $\rho_{xw^*} = cor(X, W^*|D = 0)$.

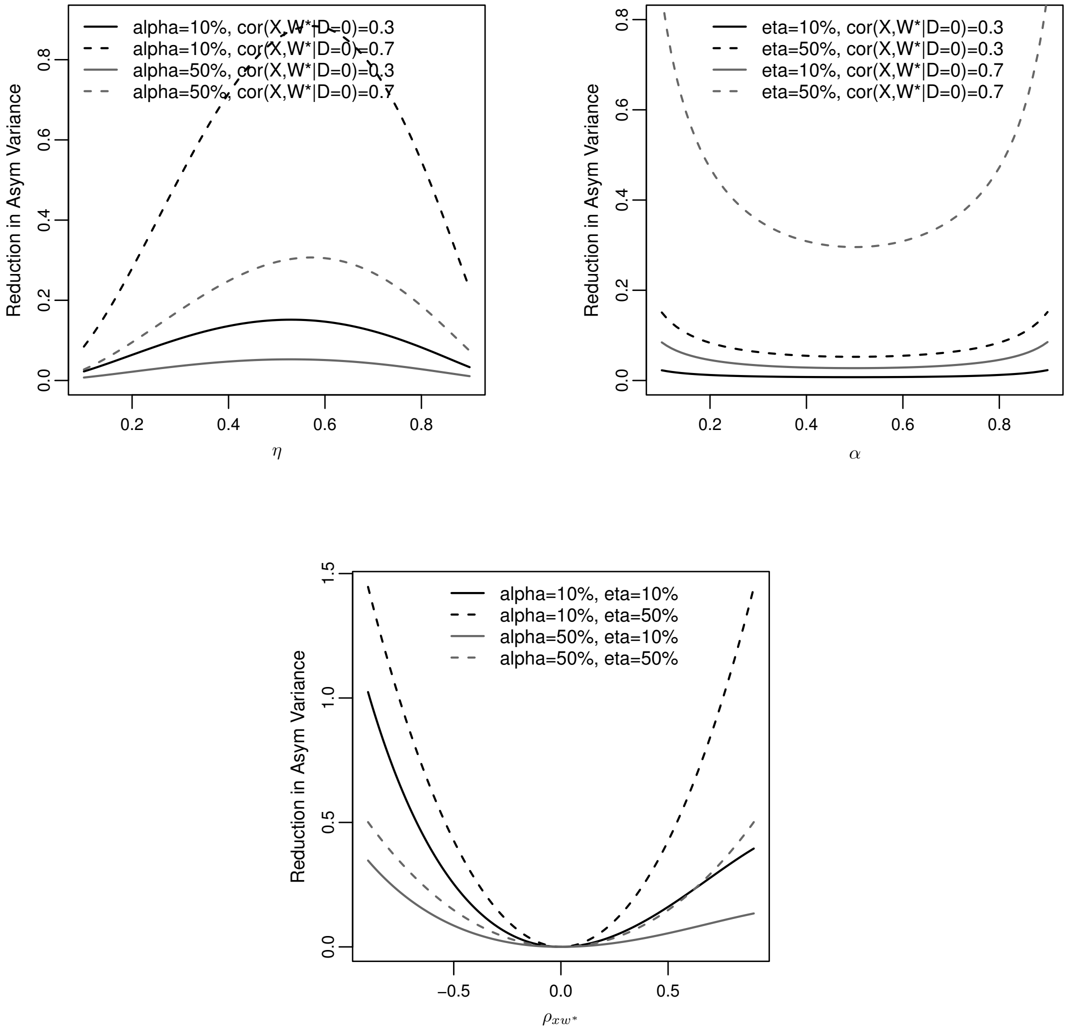

Fig. 2. Difference in asymptotic variance comparing $\sqrt{N}\left\{\widehat{AUC}_x(p) - AUC_x\right\}$ and $\sqrt{N}\left\{\widehat{AUC}_x(\hat{p}) - AUC_x\right\}$ for the variance component that is due to variability in controls, as a function of η, α, and $\rho_{xw^\star}$, for the setting described in Appendix E1. The value presented in Y-axis is the value in (0.8) assuming $AUC_x = 0.76$. Here Here $\eta = P(W = 1|D = 0)$, $\alpha = P(W = 1|D = 0, \text{Sampled in phase two})$, and $\rho_{xw^*} = cor(X, W^*|D = 0)$.

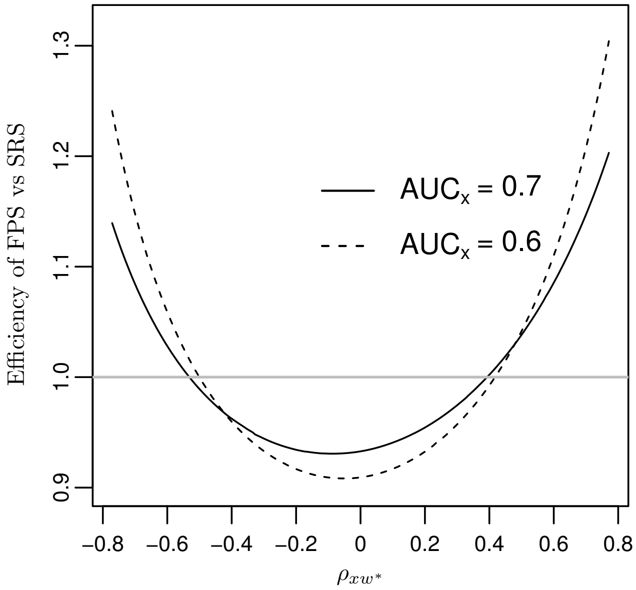

Fig. 3. Efficiency of $\widehat{AUC}(\hat{p})$ in finite-population stratified sampling (FPS) of controls relative to the empirical AUC estimator in simple random sampling without replacement (SRS) of controls, for biomarker following bi-gamma model (Appendix H2). Efficiency = asymptotic variance of AUC estimator in SRS / asymptotic variance of AUC estimator in FPS.
